# Synthesis of phosphiranes *via* organoiron-catalyzed phosphinidene transfer to electron-deficient olefins†

**DOI:** 10.1039/d2sc05011k

**Published:** 2022-10-14

**Authors:** Tiansi Xin, Michael B. Geeson, Hui Zhu, Zheng-Wang Qu, Stefan Grimme, Christopher C. Cummins

**Affiliations:** Department of Chemistry, Massachusetts Institute of Technology Cambridge MA 02139 USA ccummins@mit.edu; Mulliken Center for Theoretical Chemistry, University of Bonn Beringstr. 4 53115 Bonn Germany qu@thch.uni-bonn.de

## Abstract

Herein is reported the structural characterization and scalable preparation of the elusive iron–phosphido complex FpP(^*t*^Bu)(F) (2-F, Fp = (Fe(η^5^-C_5_H_5_)(CO)_2_)) and its precursor FpP(^*t*^Bu)(Cl) (2-Cl) in 51% and 71% yields, respectively. These phosphide complexes are proposed to be relevant to an organoiron catalytic cycle for phosphinidene transfer to electron-deficient alkenes. Examination of their properties led to the discovery of a more efficient catalytic system involving the simple, commercially available organoiron catalyst Fp_2_. This improved catalysis also enabled the preparation of new phosphiranes with high yields (^*t*^BuPCH_2_CHR; R = CO_2_Me, 41%; R = CN, 83%; R = 4-biphenyl, 73%; R = SO_2_Ph, 71%; R = POPh_2_, 70%; R = 4-pyridyl, 82%; R = 2-pyridyl, 67%; R = PPh_3_^+^, 64%) and good diastereoselectivity, demonstrating the feasibility of the phosphinidene group-transfer strategy in synthetic chemistry. Experimental and theoretical studies suggest that the original catalysis involves 2-X as the nucleophile, while for the new Fp_2_-catalyzed reaction they implicate a diiron–phosphido complex Fp_2_(P^*t*^Bu), 4, as the nucleophile which attacks the electron-deficient olefin in the key first P–C bond-forming step. In both systems, the initial nucleophilic attack may be accompanied by favorable five-membered ring formation involving a carbonyl ligand, a (reversible) pathway competitive with formation of the three-membered ring found in the phosphirane product. A novel radical mechanism is suggested for the new Fp_2_-catalyzed system.

## Abstract 2

The iconic cyclopentadienyliron dicarbonyl dimer ([CpFe(CO)_2_]_2_ or Fp_2_) catalyzes phosphinidene group transfer from anthracene to activated alkenes, forming new phosphiranes diastereoselectively and with good isolated yields.

## Graphical abstract

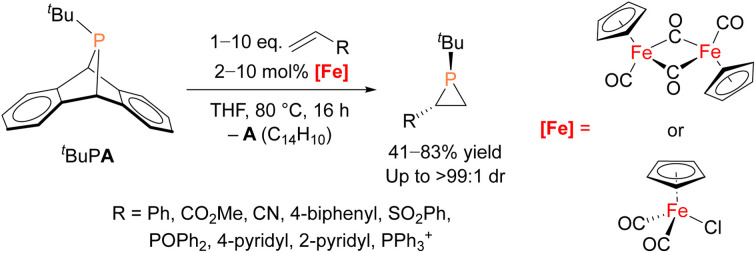

Phosphiranes, the phosphorus analogues of aziridines, have been used as ligands,^1–8^ as polymer precursors,^9,10^ and more recently as precursors to *P*,*N*-bidentate ligands *via* ring-opening reactions using amide nucleophiles.^11^ Given the possibility of chirality at both phosphorus and carbon in the three-membered ring,^7,12^ such ring-opening reactions have the potential to enable preparation of enantiomerically pure ligands from phosphiranes.

In contrast to the well-established alkene aziridination reactions that are facilitated by transition-metal catalysts,^13,14^ only a handful of transition-metal promoted phosphirane syntheses, namely “phosphiranation” reactions, have been reported,^15–19^ among which there are only two examples of catalytic alkene phosphiranation processes yielding free (unprotected) phosphiranes.^20,21^ We recently reported a catalytic method for preparing phosphiranes using a phosphinidene group-transfer strategy, mirroring the analogous aziridination reactions.^20^ The system consisted of dibenzo-7-phosphanorbornadienes (RPA, A = C_14_H_10_, anthracene), a class of compounds capable of transferring phosphinidene groups to unsaturated molecules upon loss of an aromatic moiety,^22–30^ as the phosphinidene source and styrene as the receptor (Fig. 1A). In addition, both a source of the cyclopentadienyliron dicarbonyl cation ([CpFe(CO)_2_]^+^, Fp^+^) and the fluoride anion were required as co-catalysts for phosphinidene group transfer. The reaction was postulated to proceed *via* an iron–phosphido intermediate (FpP(^*t*^Bu)(F), 2-F, Fig. 1A) based on evidence provided by stoichiometric reactions, deuterium labeling studies, and a Hammett analysis. However, the intermediate 2-F eluded isolation and full characterization due to its instability and high solubility in organic solvents.

**Fig. 1:**
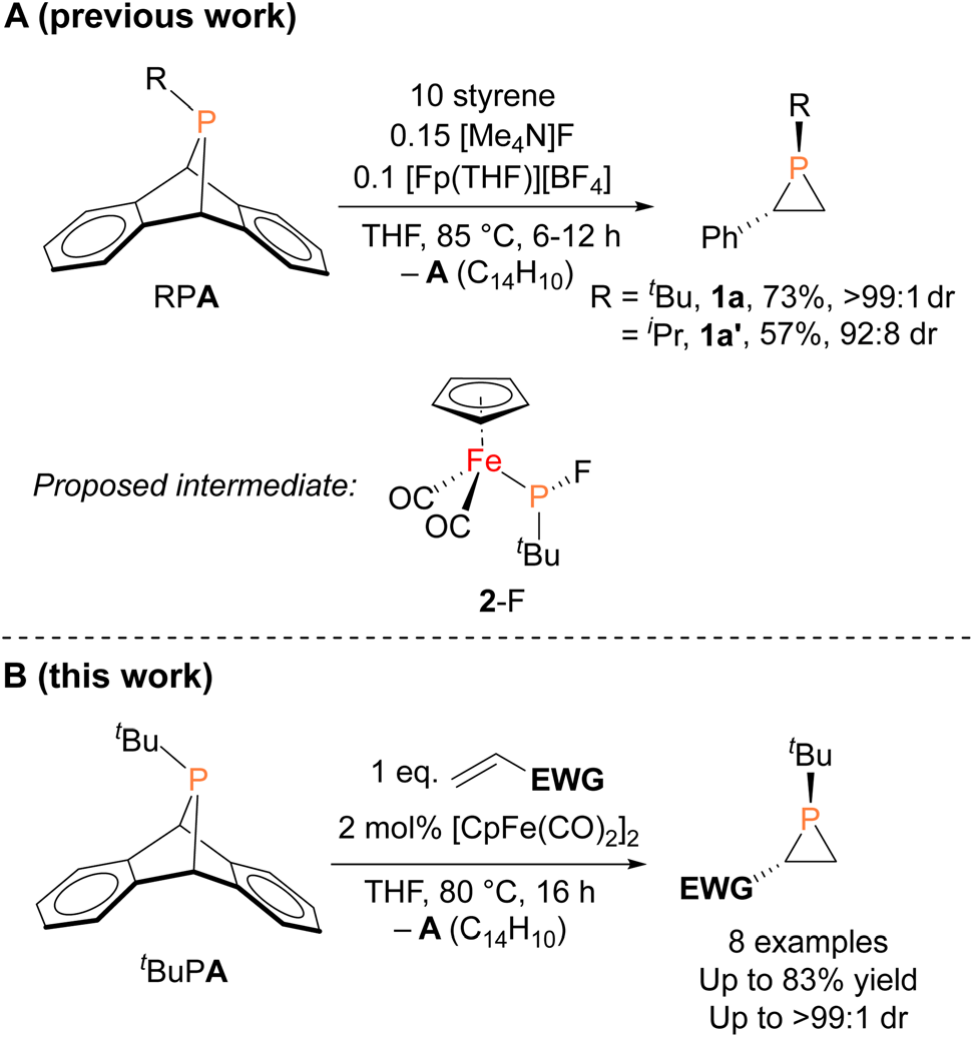
(A) Organoiron- and fluoride-catalyzed synthesis of phosphiranes by phosphinidene transfer and the proposed intermediate. (B) This work: organoiron-catalyzed phosphinidene transfer to electron-deficient olefins (EWG = electron-withdrawing group).

Herein, we report the isolation and structural characterization of 2-F, thus providing a more complete picture of the catalytic cycle. In addition, we report the discovery of a more efficient catalytic system involving a simple, commercially available organoiron catalyst, the iconic cyclopentadienyliron dicarbonyl dimer ([CpFe(CO)_2_]_2_ or Fp_2_) for this phosphiranation reaction (Fig. 1B). This improved system was also used to prepare new phosphiranes bearing electron-withdrawing substituents, expanding the list of chemically accessible phosphiranes that may serve as useful ligands and synthetic building blocks.

The iron–phosphido complex 2-F was identified in our previous work as a plausible intermediate in the catalytic cycle,^20^ and therefore became the target of an independent synthesis. This species was previously generated *in situ* by treating [Fp(^*t*^BuPA)][BF_4_] with a source of fluoride to elicit anthracene elimination, though at the time it eluded purification and complete characterization. A new, scalable preparation of 2-F was achieved by utilizing the readily available ^*t*^BuPCl_2_ and K[Fp]^31^ which, when combined in an equimolar ratio in THF, afforded 2-Cl in 71% yield (Scheme 1). Such halide displacement reactions at phosphorus were previously used to prepare analogous metal–phosphido complexes.^32–34^ In a subsequent step, halogen exchange with tetramethylammonium fluoride ([Me_4_N]F) in CH_2_Cl_2_ led to the isolation of 2-F in 51% yield (Scheme 2). In addition to characterization by NMR spectroscopy, both 2-Cl and 2-F were structurally characterized by X-ray crystallography (Fig. 2). Both compounds exhibit a pyramidal geometry at the phosphorus atom, with the sum of bond angles being 316° and 314° for 2-Cl and 2-F, respectively. The Fe–P distances in 2-Cl and 2-F are in the range of a Fe–P single bond, similar to other reported Fp–phosphido complexes.^33,35,36^

**Scheme 1:**
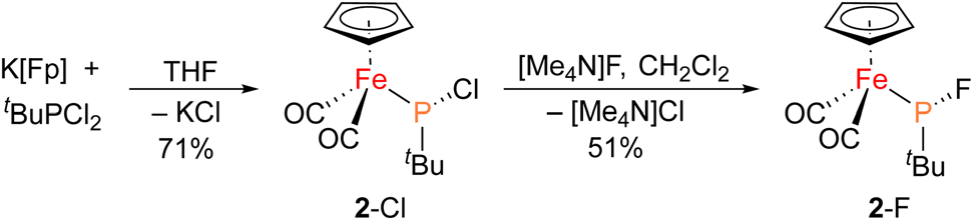
Synthesis of 2-Cl and 2-F starting from K[Fp] and ^*t*^BuPCl_2_.

**Scheme 2:**
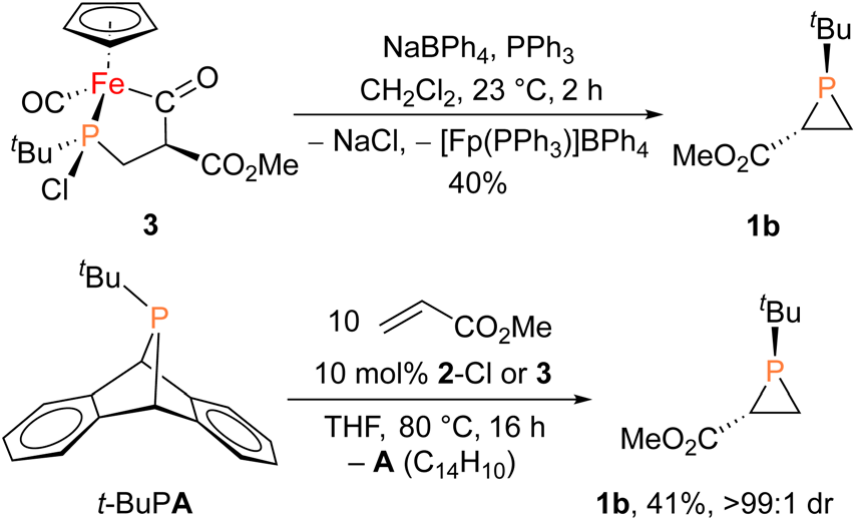
Stoichiometric (top) and catalytic (bottom) synthesis of 1b.

**Fig. 2:**
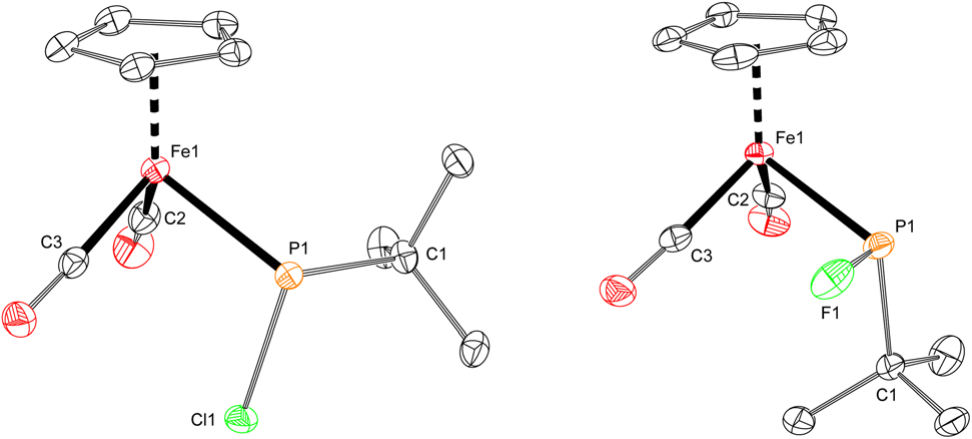
Solid-state structures of 2-Cl (left) and 2-F (right), with thermal ellipsoids shown at the 50% probability level and hydrogen atoms omitted for clarity. Selected bond lengths (Å) for 2-Cl: Fe1–P1: 2.2935(3); P1–Cl1: 2.1315(3). 2-F: Fe1–P1: 2.2898(4); P1–F1: 1.6455(11).

With proposed catalytic intermediate 2-F in hand, it was tested as a catalyst in the phosphiranation reaction of ^*t*^BuPA with styrene resulting in isolation of 1a with a yield similar to that originally reported, supporting the relevance of 2-F to the catalytic cycle. The chloride analogue 2-Cl was also found to catalyze the phosphiranation reaction, albeit with a lower yield (Table 1, entry 4). In fact, similar Fp–phosphido complexes have been reported as being nucleophilic at phosphorus; Fp*P(^*t*^Bu)(Cl) (Fp* = Cp*Fe(CO)_2_) was reported to react with methyl iodide to give the phosphonium iodide [Fp*P(^*t*^Bu)(Me)(Cl)][I],^33^ while FpP(Ph)_2_ could catalyze the isomerization of dimethyl maleate to dimethyl fumarate *via* reversible nucleophilic addition to the double bond.^37^ Similar reactions have also been reported for an iridium phosphido complex.^38^ When 2-Cl was treated with the electron-deficient olefin methyl acrylate, clean conversion to a single new compound 3 was observed (Fig. 3 top). Compound 3 features a five-membered organometallic ring, resulting from addition of methyl acrylate across the nucleophilic phosphorus center and into one carbonyl ligand of the Fp group. Two isomers were identified by NMR spectroscopy, and the major isomer was characterized by X-ray crystallography (Fig. 3 bottom). The formation of 3 from methyl acrylate raises the possibility that an analogous species may play a role in the catalytic cycle of the reaction employing styrene. Heating 2-Cl or 2-F in the presence of excess styrene, however, did not lead to any similar species, although it cannot be ruled out as a short-lived intermediate under the conditions of catalysis.

**Table 1:** Selected catalyst screening for styrene phosphiranation^a^

Entry	Catalyst	1a yield (%)
1	[Fp(THF)][BF_4_]/1.5[Me_4_N]F	91(73^b^)
2	[Fp(THF)][BF_4_]/1.5[^*n*^Bu_4_N]Cl	82
3	2-F	90(75^b^)
4	2-Cl	72
5	FpCl	80
6	FpI	13
7	FpOTf	4
8	FpPPh_2_	90
9	FpPCy_2_	92(76^b^)
10	Fp_2_^c^	99(78^b^)
11	Fp_2_^d^	98
12	Fe_2_(CO)_9_	63

a Conditions: ^*t*^BuPA (0.1 mmol), styrene (1 mmol), catalyst (10 mol%), THF (1 ml), 80 °C, 16 h. Yields were determined by ^31^P NMR analysis.

b Isolated yield after vacuum distillation.

c 2 mol%.

d 0.5 mol%, 6 h reaction time.

**Fig. 3:**
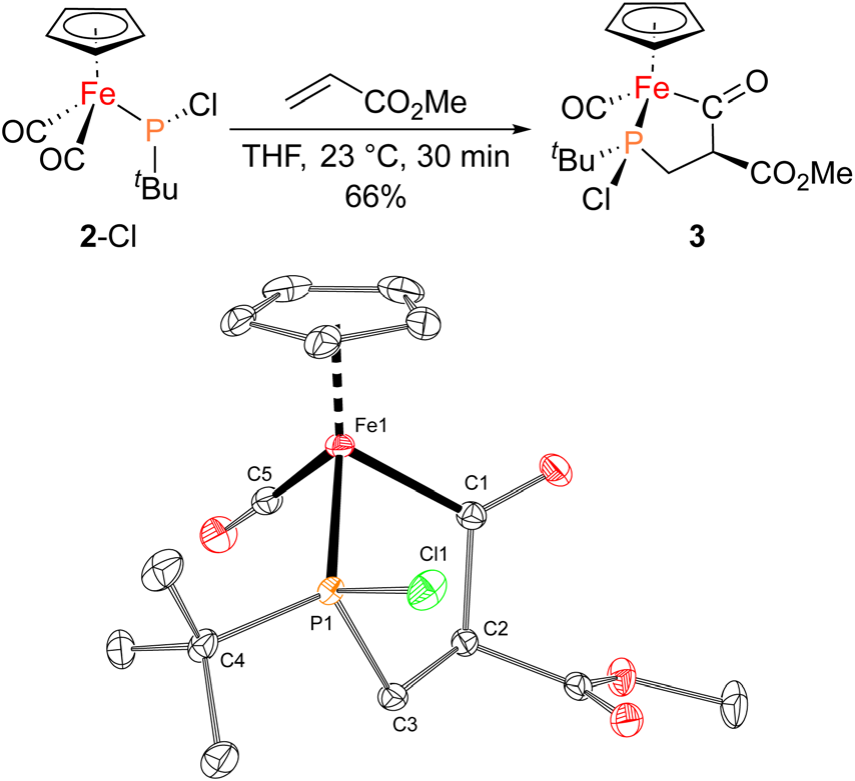
(Top) Synthesis of 3 from 2-Cl and methyl acrylate. (Bottom) Solid-state structure of 3 with thermal ellipsoids at the 50% probability level and hydrogen atoms omitted for clarity. Selected bond lengths (Å): Fe1–C1: 1.9625(8); C1–C2: 1.5960(10); C2–C3: 1.5316(12); P1–C3: 1.8277(8); Fe1–C5: 1.7393(8); Fe1–P1: 2.1335(2).

Given the potential relevance of 3 to the catalytic cycle, stoichiometric reactions to effect phosphirane release were attempted. Treating isolated 3 with NaBPh_4_ and PPh_3_ in CH_2_Cl_2_ resulted in the formation of the corresponding phosphirane 1b in *ca.* 40% conversion within 2 h (Scheme 2), further supporting the proposed catalytic cycle. Employing a catalytic amount of 2-Cl, 1b can be prepared from ^*t*^BuPA and methyl acrylate in 41% isolated yield as a single diastereomer, while a control experiment without any catalyst led to only minor amounts of phosphirane 1b.

These findings clearly revealed that fluoride is not essential for the catalytic phosphiranation reaction. We therefore set out to screen more catalysts (or precatalysts) and conditions with a view to optimizing and simplifying the reaction system (Table 1 and S1†). We first replaced the fluoride source ([Me_4_N]F) with a chloride source ([^*n*^Bu_4_N]Cl), resulting in a slightly lower yield. Similarly, isolated FpCl gave a yield of *ca.* 80%. In contrast, FpI and FpOTf exhibited little catalytic reactivity, an observation explicable in terms of the poor nucleophilicity of the anions. Interestingly, Fp-phosphido complexes FpPPh_2_ and FpPCy_2_ were also found to be efficient (pre)catalysts. A closer inspection of these catalyst systems suggested that Fp_2_, likely generated *in situ* from Fp–phosphido compounds at 80 °C, was the actual species in play. Phosphiranation proceeded smoothly with Fp_2_ even at catalyst loading as low as 0.5 mol%. Moreover, Fp_2_ is readily available from commercial sources, making it user-friendly and attractive from a practical perspective.

With the Fp_2_ catalyst system having been identified as optimal, we set out to expand the substrate scope (Table 2). Aryl-substituted olefins such as 4-vinylbiphenyl and vinylpyridines worked well in the phosphiranation reaction, giving the corresponding phosphiranes as a single diastereomer. 2-Pyridyl phosphiranes exemplified by 1h have the potential to function as chiral *P*,*N*-bidentate ligands in coordination chemistry.^39–41^ Olefins with electron-withdrawing groups such as sulfonyl, phosphine oxide, and phosphonium also gave acceptable yields with excellent diastereoselectivity. The Fp_2_ catalyst was essential for phosphirane formation, with the exception of 1c which was formed from ^*t*^BuPA and acrylonitrile in the absence of any catalyst, albeit with diminished diastereoselectivity (91 : 9) compared to other substrates.

**Table 2:** Scope of olefin phosphiranation^a^

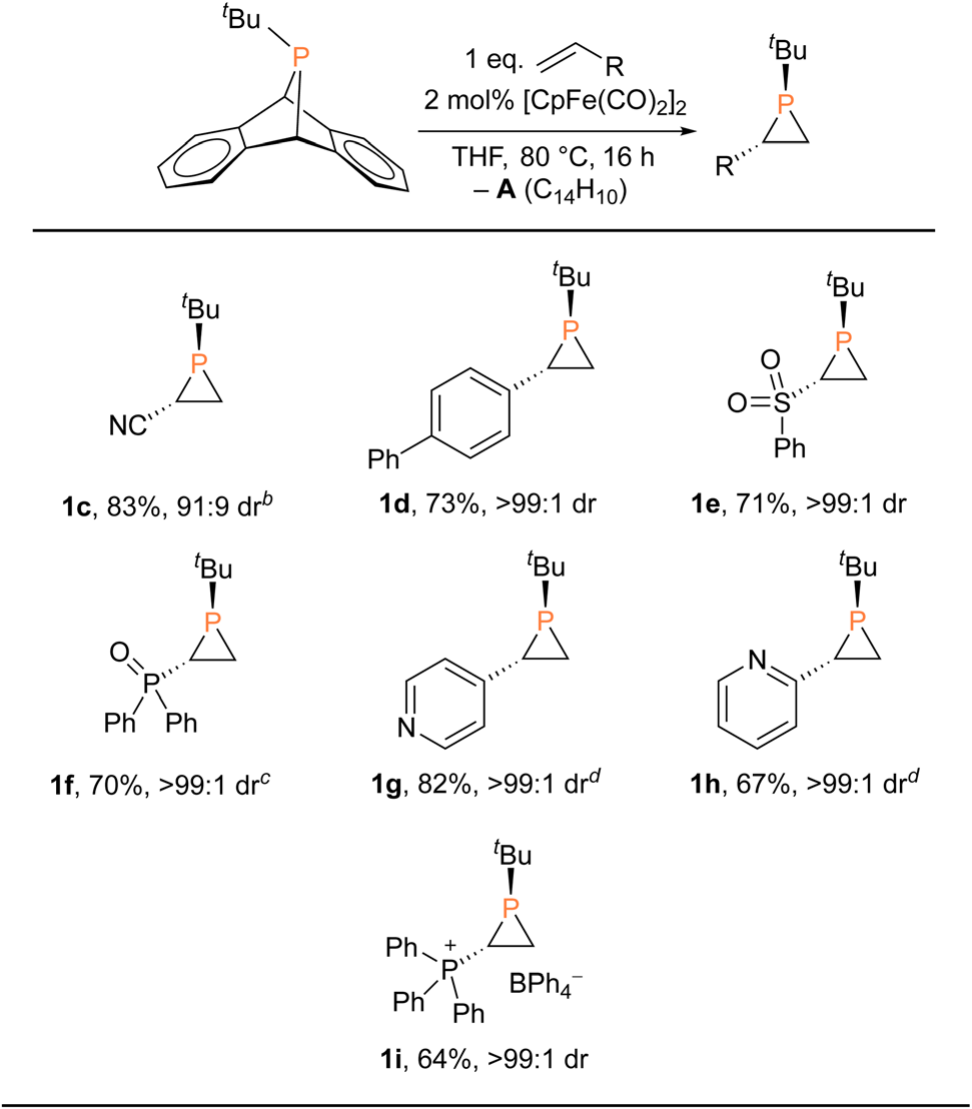

a Reactions were conducted at 1.0 mmol scale. Isolated yields.

b 10 equiv. of olefin, no catalyst employed.

c 72 h.

d Isolated as the BPh_3_ adduct.

In order to shed light on a plausible mechanism for the new Fp_2_ catalyst system, some possible intermediates and other relevant species were isolated and characterized (Fig. 4). Treatment of ^*t*^BuPA with Fp_2_ at 80 °C in the absence of an olefin trap led to the formation of anthracene and three new Fe–P containing species. Compound 4 (^31^P *δ* 166 ppm) corresponds to phosphinidene insertion into Fp_2_ and compound 5 (^31^P *δ* 623 ppm) results from decarbonylation of 4, while 6 (^31^P *δ* 55 ppm) corresponds to two phosphinidne units inserted into Fp_2_. Complexes 4–6 were initially assigned by comparing their chemical shifts to similar known compounds,^42,43^ and in the case of 4 and 6 verified by independent synthesis. Compound 4 was prepared using salt metathesis upon treatment of ^*t*^BuPCl_2_ with K[Fp], or by treating ^*t*^BuPH_2_ with benzylpotassium (KCH_2_Ph) followed by [Fp]I. Compound 6 was synthesized from [Fp(^*t*^BuPH_2_)][BF_4_] and 2-Cl in the presence of DBU. Interestingly, when treated with styrene at 80 °C, only 4 was found to afford phosphirane 1a, while 5 remained unreacted and 6 yielded the tetraphosphetane (P_4_(^*t*^Bu)_4_) as the major product. These findings suggest that 4 could be a key intermediate in the catalytic alkene phosphiranation reaction. In addition, 4 was found to readily convert to 5*via* decarbonylation under reduced pressure or light exposure at room temperature, unlike the known phenyl derivative.^42^ Unfortunately, we were unable to obtain analytically pure 4 free of the Fp_2_ and 5 impurities due to their very similar solubilities, and we were also unable to obtain a crystal structure of 4 due to its high solubility and instability.

**Fig. 4:**
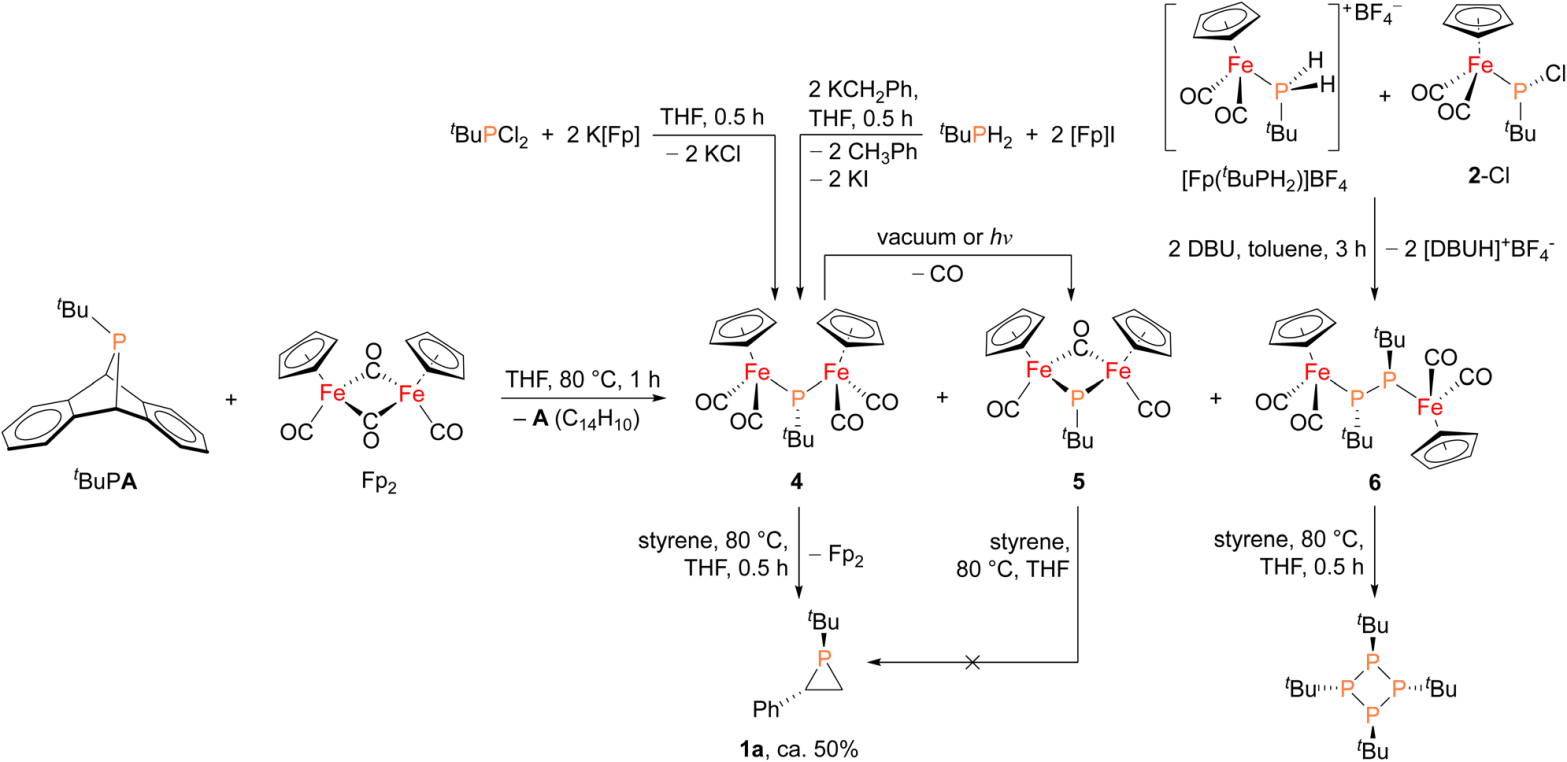
Synthesis of possible reaction intermediates 4, 5 and 6, and their reactivity studies towards styrene.

Possible pathways for the catalytic formation of phosphirane were investigated using DFT calculations at the PW6B95-D3/def2-QZVP + COSMO-RS level of theory using TPSS-D3/def2-TZVP + COSMO optimized geometries in THF solution^44–52^ that has been well tested in recent mechanistic studies.^53,54^ We were able to locate many of the intermediates and transition states along the potential energy surface. The free energy change for the net phosphiranation reaction (^*t*^BuPA + styrene → 1a + anthracene) was calculated to be −10.9 kcal mol^−1^.

For the 2-Cl (or FpCl) catalyzed reaction, as shown in Fig. 5A, the Cl^−^/^*t*^BuPA ligand exchange at the iron center of mononuclear complex FpCl is 3.5 kcal mol^−1^ endergonic to form the cationic complex [Fp(^*t*^BuPA)]^+^, from which nucleophilic Cl^−^ attack at the phosphorus center may lead to compound 2-Cl with loss of anthracene. Such formal phosphinidene transfer from ^*t*^BuPA to FpCl is −10.7 kcal mol^−1^ exergonic over a free energy barrier of 19.1 kcal mol^−1^. Using styrene as the olefin substrate, the frustrated Lewis pair (FLP)-like alkene addition to 2-Cl across the Lewis basic phosphorus atom and a Lewis acidic CO ligand is −1.6 kcal mol^−1^ exergonic over a sizable barrier of 28.2 kcal mol^−1^ (*via*TS2) to form the five-membered-ring adduct 3a, a result in agreement with the requirement of moderate heating at 80 °C under experimental conditions. Further ring-contraction of 3a is 1.4 kcal mol^−1^ endergonic over a 2.8 kcal mol^−1^ lower barrier of 25.4 kcal mol^−1^ (*via*TS3 through transient ionic [Fp(1a)]^+^ and Cl^−^ species) to release the phosphirane product 1a along with regenerated FpCl, consistent with the absence of 3a as an observable product despite its considerable kinetic stability. In contrast, a more electron-deficient olefin, methyl acrylate, turns out to be much more reactive due to more facile electrophilic alkene addition to 2-Cl, an addition now −5.6 kcal mol^−1^ exergonic over a low barrier of 17.2 kcal mol^−1^ (*via*bTS2; see ESI Fig. S52†), in good agreement with the formation of 3 observed experimentally at room temperature. Due to a higher barrier of 20.9 kcal mol^−1^ (*via* less stable 1b and bTS3), the regeneration of 2-Cl from FpCl and ^*t*^BuPA becomes the rate-limiting step.

**Fig. 5:**
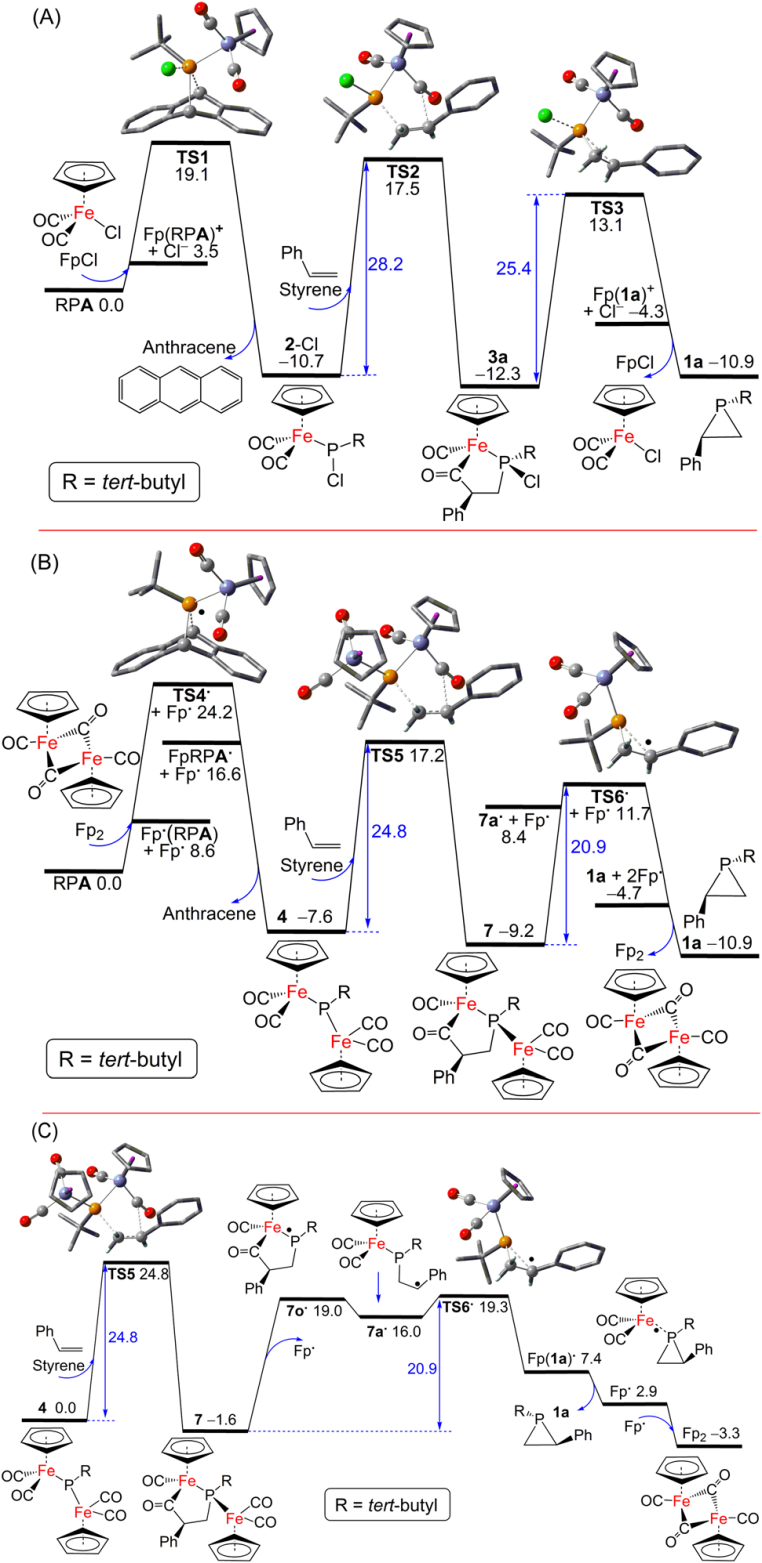
DFT computed free energy paths (in kcal mol^−1^, at 298 K and 1 M in THF solution) for (A) 2-Cl (or FpCl) and (B) 4 (or Fp_2_) catalyzed phosphinidene transfer from ^*t*^BuPA to styrene, and (C) the key P–C bond formation steps from 4. Crucial C, O, P, Cl and Fe atoms in the transition state ball-and-stick models are highlighted as grey, red, orange, green and blue balls, respectively, with most H-atoms omitted for clarity.

Interestingly, as shown in Fig. 5B, a [Fp]˙ radical is accessible from the Fp_2_ dimer complex to induce facile anthracene release from ^*t*^BuPA, a microscopic step that is −7.6 kcal mol^−1^ exergonic over a barrier of 24.2 kcal mol^−1^ (*via*TS4˙ and Fp(^*t*^BuPA)˙ radical) to form the diiron–phosphido complex 4. The phosphine adducts of the [Fp]˙ radical, formally 19-electron complexes, have been proposed in established experimental studies.^55^ Such a radical mechanism is also supported by the experimental observation of complex 6 resulting from radical–radical coupling of transient radical [FpP(^*t*^Bu)]˙ intermediate. Subsequent addition of styrene to 4 in an FLP-like process is still −1.6 kcal mol^−1^ exergonic over a slightly higher barrier of 24.8 kcal mol^−1^ (*via*TS5) to form the five-membered-ring adduct 7. The second rate-limiting barrier (TS5) is 3.4 kcal mol^−1^ lower than the first rate-limiting barrier (TS2), consistent with the evidently higher catalytic activity of 4 (or Fp_2_) than 2-Cl (or FpCl). Dissociation of the [Fp]˙ radical from 7 may induce the cyclic C–C bond cleavage of the transient P-centered radical 7o˙ to form the acyclic benzyl radical 7a˙, which is 17.6 kcal mol^−1^ endergonic without transition state (Fig. 5C). The final P–C bond formation proceeds *via*TS6˙ and [Fp(1a)]˙, the phosphirane adduct of [Fp]˙, to produce phosphirane 1a and a second [Fp]˙ radical which recombines with the first to regenerate the closed-shell Fp_2_ catalyst, making the overall ring-contraction step 1.7 kcal mol^−1^ exergonic over a moderate barrier of 20.9 kcal mol^−1^. Alternatively, the reactive [FpP(^*t*^Bu)]˙ radical could also be formed from 4*via* [Fp]˙ elimination and then react with styrene to afford 1a, which however encounters a high barrier of 36.2 kcal mol^−1^ (*via*TS7 and TS7c; see ESI†). The decarbonylation of 4 is endergonic by 6.3 kcal mol^−1^ to form the complex 5 with a barrier of approximately 15 kcal mol^−1^, making 4 prone to decomposition under reduced pressure or light exposure. Much higher barriers are found for other conceivable ionic pathways for the first and the third catalytic steps, which are thus kinetically unfavorable.

In conclusion, we have achieved a scalable preparation and characterization of an elusive iron–phosphido complex 2-F, which was previously proposed as a key intermediate in the organoiron- and fluoride-catalyzed styrene phosphiranation reaction, from its precursor 2-Cl. Examination of the properties of 2-X (X = F, Cl) led to the isolation of another potential intermediate in the catalytic cycle, as well as the discovery of a more efficient catalytic system consisting of a simple, commercially available organoiron catalyst Fp_2_, RPA, and an electron-deficient olefin. In the new system, the catalyst loading could be lowered to 2 mol%, and only stoichiometric amounts of alkene substrate were required. The new and improved catalyst system also enabled the preparation of several new phosphiranes bearing electron-withdrawing groups with satisfying yields and excellent diastereoselectivity. Unlike the original catalytic system which is understood to proceed through 2-X and 3a*via* fully ionic pathways, this new reaction is postulated to proceed through a more reactive diiron–phosphido intermediate 4 and a five-membered iron–phosphorus–carbon ring intermediate 7*via* a novel radical mechanism involving [Fp]˙. The present findings enhance the expanding library of known phosphiranes, while further highlighting the feasibility of the transition-metal catalyzed phosphinidene group-transfer strategy in synthetic chemistry.

## Data availability

The experimental details, characterization data, NMR spectra, crystallography and computational details associated with this article are provided in the ESI.† Crystallographic data for compound 2-Cl, 2-F and 3 has also been deposited at the CCDC under 2190120—2190122.

## Author contributions

T. X. and M. B. G. conducted experiments and wrote the manuscript. C. C. C. supervised the work and finalized the manuscript. H. Z., Z.-W. Q. and S. G. conducted DFT mechanistic study and partially wrote the manuscript.

## Conflicts of interest

There are no conflicts to declare.

## Supplementary Material

SC-013-D2SC05011K-s001

SC-013-D2SC05011K-s002

SC-013-D2SC05011K-s003

SC-013-D2SC05011K-s004

SC-013-D2SC05011K-s005

SC-013-D2SC05011K-s006

SC-013-D2SC05011K-s007

SC-013-D2SC05011K-s008

SC-013-D2SC05011K-s009

SC-013-D2SC05011K-s010

SC-013-D2SC05011K-s011

SC-013-D2SC05011K-s012

SC-013-D2SC05011K-s013

SC-013-D2SC05011K-s014

SC-013-D2SC05011K-s015

SC-013-D2SC05011K-s016

SC-013-D2SC05011K-s017

SC-013-D2SC05011K-s018

SC-013-D2SC05011K-s019

SC-013-D2SC05011K-s020

SC-013-D2SC05011K-s021

SC-013-D2SC05011K-s022

SC-013-D2SC05011K-s023

SC-013-D2SC05011K-s024

SC-013-D2SC05011K-s025

SC-013-D2SC05011K-s026

SC-013-D2SC05011K-s027

SC-013-D2SC05011K-s028

SC-013-D2SC05011K-s029

SC-013-D2SC05011K-s030

SC-013-D2SC05011K-s031

SC-013-D2SC05011K-s032

SC-013-D2SC05011K-s033

SC-013-D2SC05011K-s034

SC-013-D2SC05011K-s035

SC-013-D2SC05011K-s036

SC-013-D2SC05011K-s037

SC-013-D2SC05011K-s038

SC-013-D2SC05011K-s039

SC-013-D2SC05011K-s040

SC-013-D2SC05011K-s041

SC-013-D2SC05011K-s042

SC-013-D2SC05011K-s043

SC-013-D2SC05011K-s044

SC-013-D2SC05011K-s045

SC-013-D2SC05011K-s046

SC-013-D2SC05011K-s047

SC-013-D2SC05011K-s048

SC-013-D2SC05011K-s049

SC-013-D2SC05011K-s050

SC-013-D2SC05011K-s051

SC-013-D2SC05011K-s052

SC-013-D2SC05011K-s053

SC-013-D2SC05011K-s054

SC-013-D2SC05011K-s055

SC-013-D2SC05011K-s056

SC-013-D2SC05011K-s057

SC-013-D2SC05011K-s058

SC-013-D2SC05011K-s059

SC-013-D2SC05011K-s060

SC-013-D2SC05011K-s061

SC-013-D2SC05011K-s062
