# Supplementary material for: Synthesis of phosphiranes via organoiron-catalyzed phosphinidene transfer to electron-deficient olefins
Source: Chem Sci. 2022 Oct 14;13(43):12696–702. doi: 10.1039/d2sc05011k (PMC9645374; doi:10.1039/d2sc05011k)
Supplement: SC-013-D2SC05011K-s001 [file SC-013-D2SC05011K-s001.pdf]

Electronic Supplementary Material (ESI) for Chemical Science.
This journal is © The Royal Society of Chemistry 2022

Supporting Information: Synthesis of phosphiranes via organoiron-catalyzed phosphinidene transfer to electron-deficient olefins

Tiansi Xin,† Michael B. Geeson,† Hui Zhu,‡ Zheng-wang Qu,*,‡ Stefan Grimme,‡ and Christopher C. Cummins*,†

†*Department of Chemistry, Massachusetts Institute of Technology, Cambridge, MA 02139, USA*

‡*Mulliken Center for Theoretical Chemistry, University of Bonn, Beringstr. 4, 53115 Bonn, Germany*

E-mail: qu@thch.uni-bonn.de; ccummins@mit.edu

Contents

S1 Synthetic details and characterization data **S4**

S1.1 General considerations S4

S1.2 Synthesis and reactivity study of reaction intermediates S5

S1.2.1 Synthesis of **2**-Cl S5

S1.2.2 Synthesis of **2**-F S6

S1.2.3 Synthesis of **3** S6

S1.2.4 Phosphirane release from **3** S7

S1.2.5 Generation of **4**, **5** and **6** from ^{t}BuP**A** and Fp_2 . . . . S8

S1.2.6 Synthesis of **4** S8

S1.2.7 Synthesis of **6** S9

S1.2.8 Treatment of **4** with styrene S10

S1.2.9 Treatment of **6** with styrene S10

S1.3 Catalyst screening S39

S1.4 Synthesis of phosphiranes S41

S1.4.1 1-(*Tert*-butyl)-2-phenylphosphirane, **1a** S41

S1.4.2 Methyl 1-(*tert*-butyl)phosphirane-2-carboxylate, **1b** S41

S1.4.3 1-(*Tert*-butyl)phosphirane-2-carbonitrile, **1c** S42

S1.4.4 2-([1,1'-Biphenyl]-4-yl)-1-(*tert*-butyl)phosphirane, **1d** S43

S1.4.5 1-(*Tert*-butyl)-2-(phenylsulfonyl)phosphirane, **1e** S44

S1.4.6 (1-(*tert*-butyl)phosphiran-2-yl)diphenylphosphine oxide, **1f** S45

S1.4.7 4-(1-(*tert*-butyl)phosphiran-2-yl)pyridine, **1g** S45

S1.4.8 2-(1-(*tert*-butyl)phosphiran-2-yl)pyridine, **1h** S46

S1.4.9 (1-(*tert*-butyl)phosphiran-2-yl)triphenylphosphonium tetraphenylborate, **1i** S47

S2 X-ray crystallography S73

S2.1 General considerations S73

S2.2 X-ray structure of **2**-Cl S73

S2.3 X-ray structure of **2**-F S73

S2.4 X-ray structure of **3** S74

S3 Computational Details S78

S4 References S109

S1 Synthetic details and characterization data

S1.1 General considerations

All manipulations were performed in a Vacuum Atmospheres model MO-40M glovebox under an inert atmosphere of purified N_2 or using standard Schlenk techniques. All solvents were obtained anhydrous and oxygen-free by bubble degassing (argon) and purified by passing through columns of alumina and Q5.[S1] Once collected, solvents were stored over activated 4 Å molecular sieves (20 wt%) inside the glovebox.[S2] All glassware was oven-dried for at least 6 h prior to use, at temperatures greater than 150 °C. Deuterated solvents were purchased from Cambridge Isotope Labs and were degassed three times by the freeze-pump-thaw method and stored over activated 4 Å molecular sieves for 48 h in the glovebox prior to use. Diatomaceous earth (Celite 545, Millipore-Sigma), 4 Å molecular sieves (Millipore-Sigma), Activated Charcoal Norit CA1 (Millipore-Sigma) and Florisil (Millipore-Sigma) were dried by heating at 200 °C under dynamic vacuum for at least 48 h prior to use.

^{t}BuP**A**,[S3] K[Fp],[S4] [Me_4N]F,[S5] KBn,[S6] FpCl,[S7] FpI,[S8] [Fp(THF)][BF_4][S9,S10] and vinyldiphenylphosphine oxide[S11] were prepared according to literature procedures. Styrene, methyl acrylate, acrylonitrile, 4-vinylpyridine and 2-vinylpyridine were purchased from Millipore-Sigma and degassed by the freeze-pump-thaw method prior to use. Unless otherwise noted, all other chemicals were purchased commercially and used as received.

NMR spectra were obtained on Bruker Avance 400 and Bruker Neo 500 spectrometers. ^{1}H and ^{13}C NMR spectra were referenced to residual CD_2Cl_2 (^{1}H = 5.32 ppm, ^{13}C = 54.0 ppm), C_6D_6 (^{1}H = 7.16 ppm, ^{13}C = 128.06 ppm) or $CDCl_3$ (^{1}H = 7.26 ppm, ^{13}C = 77.16 ppm). ^{31}P NMR spectra were referenced externally to 85% H_3PO_4 (0 ppm).

^{19}F NMR spectra were referenced externally to hexafluorobenzene in benzene-d_6 (0.2 M, −164.9 ppm). Elemental combustion analyses were performed by Midwest Micro Laboratories (Indianapolis, IN, USA). Infrared spectra were collected using a Bruker ATR-IR Tensor 37. Samples were removed from the glovebox in sealed vials and briefly handled in air prior to data collection.

S1.2 Synthesis and reactivity study of reaction intermediates

S1.2.1 Synthesis of 2-Cl

To a thawing suspension of K[Fp] (864 mg, 4.0 mmol, 1.0 equiv) in THF (15 mL) was added a thawing solution of ^{t}BuPCl$_2$ (636 mg, 4.0 mmol, 1.0 equiv) in THF (15 mL). The mixture was stirred as it warmed to room temperature and for an additional 30 min. All volatile materials were removed under reduced pressure. The resulting residue was extracted with pentane and filtered through Celite. The residue was washed with more pentane until the filtrate became colorless. The combined filtrate was concentrated under reduced pressure to ca. 2 mL and left standing at –35 °C overnight. The formed red crystals were collected by decanting, rinsing with cold pentane and drying under vacuum to yield **2**-Cl as red crystals (871 mg, 2.9 mmol, 72%). Crystals suitable for X-ray crystallography were grown from pentane at –35 °C. ^{1}H NMR (400 MHz, benzene-d_6, Figure S1) δ 4.16 (d, J = 2.2 Hz, 5H), 1.43 (d, J = 11.9 Hz, 9H) ppm. ^{13}C NMR (126 MHz, chloroform-d, Figure S2) δ 214.91 (d, J = 4.3 Hz), 213.80 (d, J = 2.4 Hz), 87.69 (d, J = 3.7 Hz), 29.56 (d, J = 6.5 Hz), 28.68 (d, J = 19.3 Hz) ppm. ^{31}P{^{1}H} NMR (162 MHz, benzene-d_6, Figure S3) δ 279.76 (s) ppm. ATR IR (Figure S4): 2043, 1990 [ν(C≡O)$_{\text{terminal}}$] cm^{-1}.

S1.2.2 Synthesis of 2-F

All manipulations were carried out in the dark or with the reaction vessel covered by aluminum foil to protect **2**-F from being exposed to light. To a thawing solution of **2**-Cl (601 mg, 2.0 mmol, 1.0 equiv) in DCM (15 mL) was added a thawing solution of $[Me_4N]F$ (186 mg, 2.0 mmol, 1.0 equiv) in DCM (5 mL). The mixture was stirred as it warmed to room temperature and for an additional 20 min, during which time a white precipitate formed. The precipitate was removed by filtration, and volatile materials were removed from the filtrate under reduced pressure. The residue was dissolved in minimal pentane (ca. 2 mL) and the solution was left standing at –35 °C overnight. The formed dark crystals were collected by decanting, rinsing with cold pentane and drying under vacuum to yield **2**-F as dark red crystals (287 mg, 1.0 mmol, 51%). Crystals suitable for X-ray crystallography were grown from pentane at –35 °C. ^{1}H NMR (400 MHz, Methylene Chloride-d_2, Figure S5) δ 5.01 (d, J = 2.2 Hz, 5H), 1.26 (dd, J = 11.4, 1.8 Hz, 9H) ppm. ^{13}C NMR (126 MHz, Methylene Chloride-d_2, Figure S6) δ 216.04, 214.57, 87.66 (d, J = 3.7 Hz), 27.63 (d, J = 18.1 Hz) ppm. $^{19}F\{^{1}H\}$ NMR (471 MHz, Methylene Chloride-d_2, Figure S7) δ –202.57 (d, J = 823.4 Hz) ppm. $^{31}P\{^{1}H\}$ NMR (162 MHz, Methylene Chloride-d_2, Figure S8) δ 370.30 (d, J = 822.9 Hz) ppm. ATR IR (Figure S9): 2022, 1971 [$\nu(C{\equiv}O)_{terminal}$] cm^{-1}.

S1.2.3 Synthesis of 3

A solution of methyl acrylate (129 mg, 1.5 mmol, 1.0 equiv) in THF (7 mL) was added to a solution of **2**-Cl (451 mg, 1.5 mmol, 1.0 equiv) in THF (3 mL). The resulting solution was stirred at room temperature for 30 min before volatile materials were removed under reduced pressure. The residue was taken up in Et_2O (15 mL) and filtered. The filtrate

was concentrated to ca. 3 mL and left standing at –35 °C overnight. The formed crystals were collected by decanting, rinsing with cold pentane and drying under vacuum to yield **3** as yellow to orange crystals (384 mg, 1.0 mmol, 66%). Crystals suitable for X-ray crystallography were grown from diethyl ether at –35 °C. ^{1}H NMR (400 MHz, benzene-d_6, Figure S10) δ 4.31 (s, 5H), 3.55 – 3.48 (m, 1H), 3.47 (s, 3H), 2.25 (ddd, J = 14.0, 11.7, 5.4 Hz, 1H), 2.07 (ddd, J = 14.0, 12.4, 7.5 Hz, 1H), 0.97 (d, J = 16.9 Hz, 9H) ppm. ^{13}C NMR (126 MHz, chloroform-d, Figure S11) δ 215.22 (d, J = 21.8 Hz), 169.87 (d, J = 23.6 Hz), 86.56, 69.04 (d, J = 15.3 Hz), 52.42, 41.87 (d, J = 19.4 Hz), 32.39 (d, J = 14.1 Hz), 26.29 (d, J = 5.0 Hz) ppm. ^{31}P{^{1}H} NMR (162 MHz, benzene-d_6, Figure S12) δ 245.04 (s) ppm. Minor isomer: ^{1}H NMR (400 MHz, benzene-d_6, Figure S10) δ 4.36 (s, 5H), 3.51 (s, 3H), 2.68 (ddd, J = 15.3, 13.1, 6.8 Hz, 1H), 1.75 (ddd, J = 15.0, 11.3, 3.3 Hz, 1H), 0.97 (d, J = 16.9 Hz, 9H) ppm. ^{13}C NMR (126 MHz, chloroform-d, Figure S11) δ 87.44, 70.83 (d, J = 16.5 Hz), 52.24, 31.36 (d, J = 14.0 Hz), 26.12 (d, J = 5.0 Hz) ppm. ^{31}P{^{1}H} NMR (162 MHz, benzene-d_6, Figure S12) δ 244.64 (s) ppm. ATR IR (Figure S13): 1949 [$\nu(C\equiv O)_{terminal}$], 1729 [$\nu(C{=}O)_{ester}$], 1635 [$\nu(C{=}O)_{ring}$] cm^{-1}.

S1.2.4 Phosphirane release from 3

To a mixture of **3** (39 mg, 0.1 mmol, 1.0 equiv), triphenylphosphine (26 mg, 0.1 mmol, 1.0 equiv) and sodium tetraphenylborate (34 mg, 0.1 mmol, 1.0 equiv) was added DCM (3 mL). The suspension was vigorously stirred for 2 h at room temperature. The white precipitate was removed by filtration, and an aliquot of the filtrate was analyzed by ^{31}P NMR spectroscopy, showing ca. 40% conversion to **1b** (Figure S14). Volatile material was removed from the filtrate under reduced pressure, and the residue was extracted with pentane (3×5 mL). Removal of pentane in vacuo yielded **1b** (10 mg) containing ca. 20 mol% triphenylphosphine (Figure S15 and S16).

S1.2.5 Generation of 4, 5 and 6 from ^{t}BuPA and Fp_2

A solution of ^{t}BuP**A** (26 mg, 0.1 mmol, 2.0 equiv) and Fp_2 (18 mg, 0.05 mmol, 1.0 equiv) in THF (1 mL) was heated in a J-Young tube (containing a sealed capillary tube of PPh_3 solution) at 80 °C for 1 h. After cooling, the solution was analyzed by ^{31}P NMR spectroscopy (Figure S17), showing $P_3(^t\text{Bu})_3$ as the major product along with the formation of **4** (δ 166 ppm), the decarbonylation product **5** (δ 623 ppm) and **6**.

S1.2.6 Synthesis of 4

Method I: from ^{t}BuPCl$_2$ and K[Fp]. To a thawing slurry of K[Fp] (432 mg, 2.0 mmol, 2.0 equiv) in THF (8 mL) was added a thawing solution of ^{t}BuPCl$_2$ in THF (8 mL) dropwise over 2 min. The resulting mixture was stirred in the dark as it warmed to room temperature and for an additional 30 min. Volatile materials were removed under reduced pressure, and the dark red residue was extracted with pentane and filtered through Celite until the filtrate became colorless. Volatile materials were removed from the combined filtrate and the residue was recrystallized in pentane (ca. 6 mL) at –35 °C. The formed red solid was collected by decanting, rinsing with cold pentane and briefly drying in vacuo (<5 min) to yield a red solid (239 mg) containing approximately 80% of **4**. Major impurities include Fp_2 and the decarbonylation product **5**.

Method II: from ^{t}BuPH$_2$ and FpI. To a thawing solution of ^{t}BuPH$_2$ (90 mg, 1.0 mmol, 1.0 equiv) in THF (8 mL) was added KBn (260 mg, 2.0 mmol, 2.0 equiv) in one portion. The resulting mixture was stirred as it warmed to room temperature and for an additional 30 min. The resulting yellow suspension was frozen again and, upon thawing, was added dropwise to a thawing solution of FpI (608 mg, 2.0 mmol, 2.0 equiv) in THF (10 mL) over 3 min. The resulting mixture was stirred in the dark as it warmed to room temperature

and for an additional 45 min. Volatile materials were removed under reduced pressure, and the dark red residue was extracted with pentane and filtered through Celite until the filtrate became colorless. Volatile materials were removed from the combined filtrate and the residue was recrystallized twice in pentane (ca. 5 mL each time) at –35 °C. The formed red solid was collected by decanting, rinsing with cold pentane and briefly drying in vacuo (<5 min) to yield a red solid (120 mg) containing approximately 93% of **4**. Major impurities were Fp_2 (1 mol%) and the decarbonylation product **5** (6 mol%). **4**: ^{1}H NMR (400 MHz, benzene-d_6, Figure S18) δ 4.36 (d, J = 2.5 Hz, 10H), 1.52 (d, J = 9.8 Hz, 9H) ppm. ^{31}P{^{1}H} NMR (162 MHz, benzene-d_6, Figure S19) δ 165.82 (br) ppm. ATR IR (Figure S20): 2042, 2011, 1991, 1958 [ν(C≡O)$_{\text{terminal}}$] cm^{-1}. **5**: ^{1}H NMR (400 MHz, benzene-d_6, Figure S18) δ 4.25 (d, J = 1.8 Hz, 10H), 1.83 (d, J = 10.4 Hz, 9H) ppm. ^{31}P{^{1}H} NMR (162 MHz, benzene-d_6, Figure S19) δ 624.01 (s) ppm. ATR IR (Figure S20): 1976 [ν(C≡O)$_{\text{terminal}}$], 1783 [ν(C≡O)$_{\text{bridging}}$] cm^{-1}.

S1.2.7 Synthesis of 6

To a pre-cooled (–35 °C) solution of [Fp(THF)][BF_4] (168 mg, 0.50 mmol, 1.0 equiv) in DCM (8 mL) was added dropwise a solution of ^{t}BuPH_2 (50 mg, 0.55 mmol, 1.1 equiv) in hexanes (0.7 mL). The resulting solution was stirred in the dark at room temperature for 1 h, during which time the color of the solution turned from red to yellow. Volatile materials were removed under reduced pressure to yield [Fp(^{t}BuPH_2)][BF_4] (177 mg, 100%) as a yellow solid, which was used in the next step without any purification. ^{1}H NMR (400 MHz, chloroform-d, Figure S21) δ 5.52 (d, J = 2.0 Hz, 5H), 5.23 (d, J = 379.2 Hz, 2H), 1.38 (d, J = 18.6 Hz, 9H) ppm. ^{31}P{^{1}H} NMR (162 MHz, chloroform-d, Figure S22) δ 3.13 (s) ppm.

To a 20 mL scintillation vial charged with [Fp(^{t}BuPH_2)][BF_4] (35 mg, 0.10 mmol,

1.0 equiv), **2**-Cl (30 mg, 0.10 mmol, 1.0 equiv) and a magnetic stir bar was added 5 mL of toluene. With stirring, to the resulting suspension was added DBU (45 mg, 0.30 mmol, 3.0 equiv) dropwise. The mixture was stirred in the dark for 3 h. Volatile materials were removed under reduced pressure, and the residue was rinsed with 3×3 mL of pentane, then extracted with 2×5 mL of diethyl ether. Volatile materials were removed from the combined diethyl ether extract to yield **6** as a dark red oil (22 mg, 0.042 mmol, 42%). ^{1}H NMR (400 MHz, chloroform-*d*, Figure S23) δ 4.86 (s, 10H), 1.33 (t, J = 6.2 Hz, 18H) ppm. ^{31}P{^{1}H} NMR (162 MHz, chloroform-*d*, Figure S24) δ 55.15 (s) ppm. ATR IR (Figure S25): 2012, 1960 [$\nu(C{\equiv}O)_{terminal}$].

S1.2.8 Treatment of 4 with styrene

A solution of isolated **4** (containing 6 mol% of **5** and 1 mol% of Fp_2) (9 mg, 0.02 mmol, 1.0 equiv) and styrene (21 mg, 0.2 mmol, 10 equiv) in THF (0.7 mL) was heated in a J-Young tube covered by aluminum foil at 80 °C for 0.5 h. After cooling, the solution was analyzed by ^{31}P NMR spectroscopy (Figure S26), showing formation of **1a** (δ –165 ppm, ca. 50%), the decarbonylation product **5** (δ 623 ppm, ca. 20%) and a new species that is tentatively assigned as **7** (δ 135 ppm, ca. 30%). Prolonged heating of the solution led to depletion of **7** and more formation of both **1a** and **5**.

S1.2.9 Treatment of 6 with styrene

A solution of isolated **6** (11 mg, 0.02 mmol, 1.0 equiv) and styrene (21 mg, 0.2 mmol, 10 equiv) in THF (0.7 mL) was heated in a J-Young tube covered by aluminum foil at 80 °C for 0.5 h. After cooling, the solution was analyzed by ^{31}P NMR spectroscopy (Figure S27), showing $P_4(^tBu)_4$ as the major product (δ –58 ppm, ca. 70%) along with some $P_3(^tBu)_3$ (δ –71, –110 ppm, ca. 20%) and the decarbonylation product **5** (δ 623

ppm, ca. 10%). No **1a** was detected.

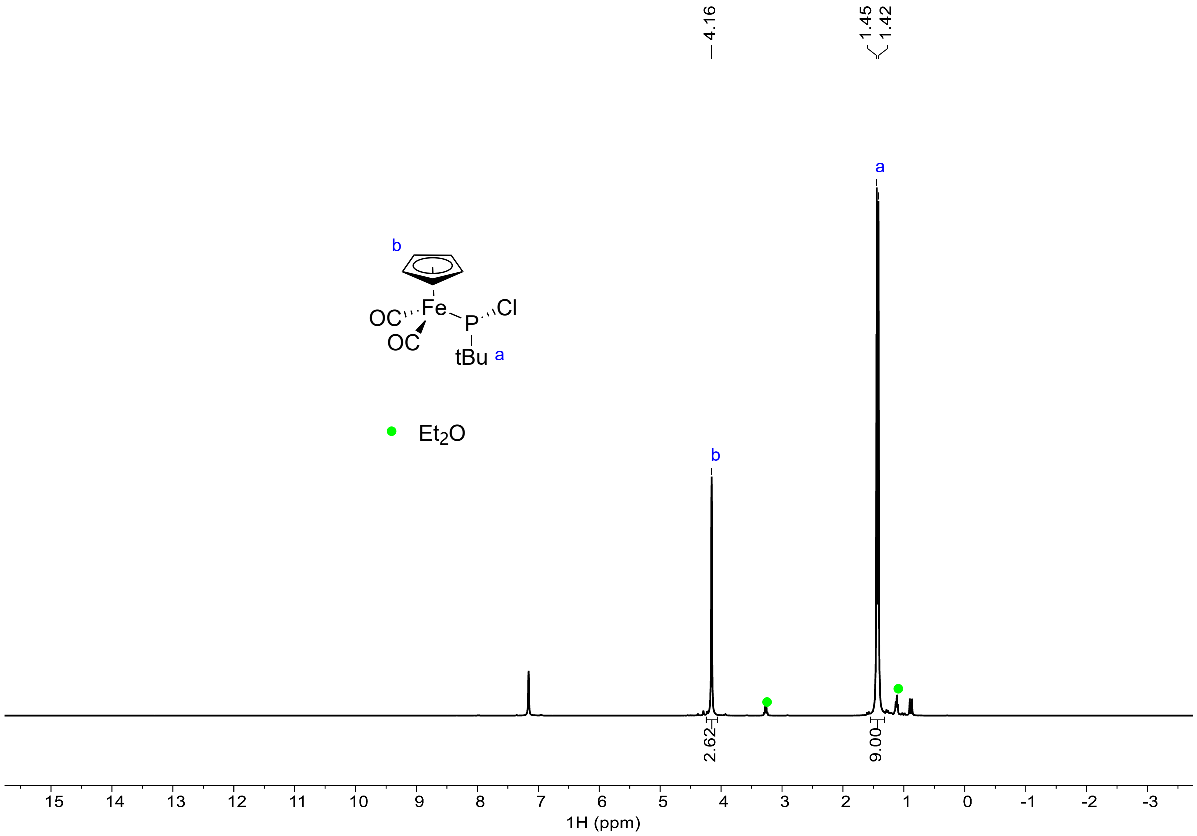

Figure S1: ^{1}H NMR spectrum of **2**-Cl in C_6D_6 at 25 °C, recorded at 400 MHz.

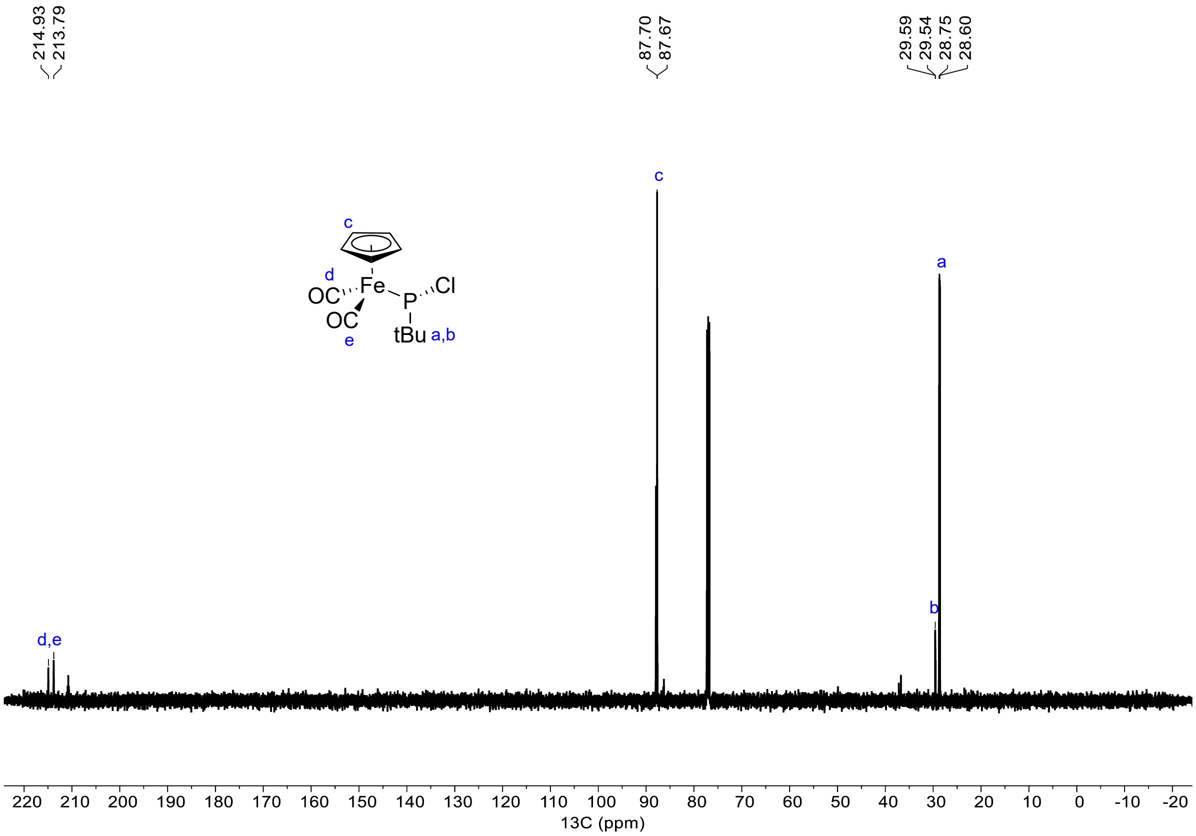

Figure S2: ^{13}C NMR spectrum of **2**-Cl in $CDCl_3$ at 25 °C, recorded at 126 MHz.

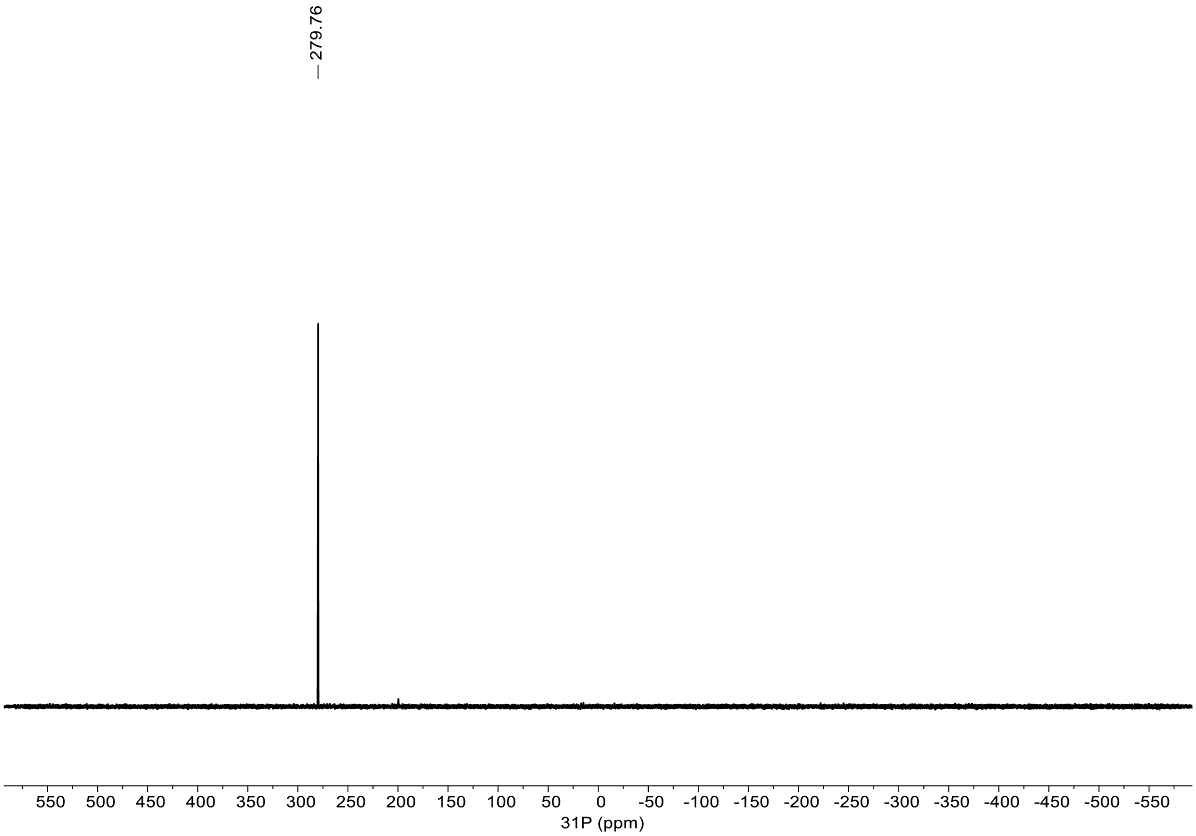

Figure S3: $^{31}P\{^{1}H\}$ NMR spectrum of **2**-Cl in C_6D_6 at 25 °C, recorded at 162 MHz.

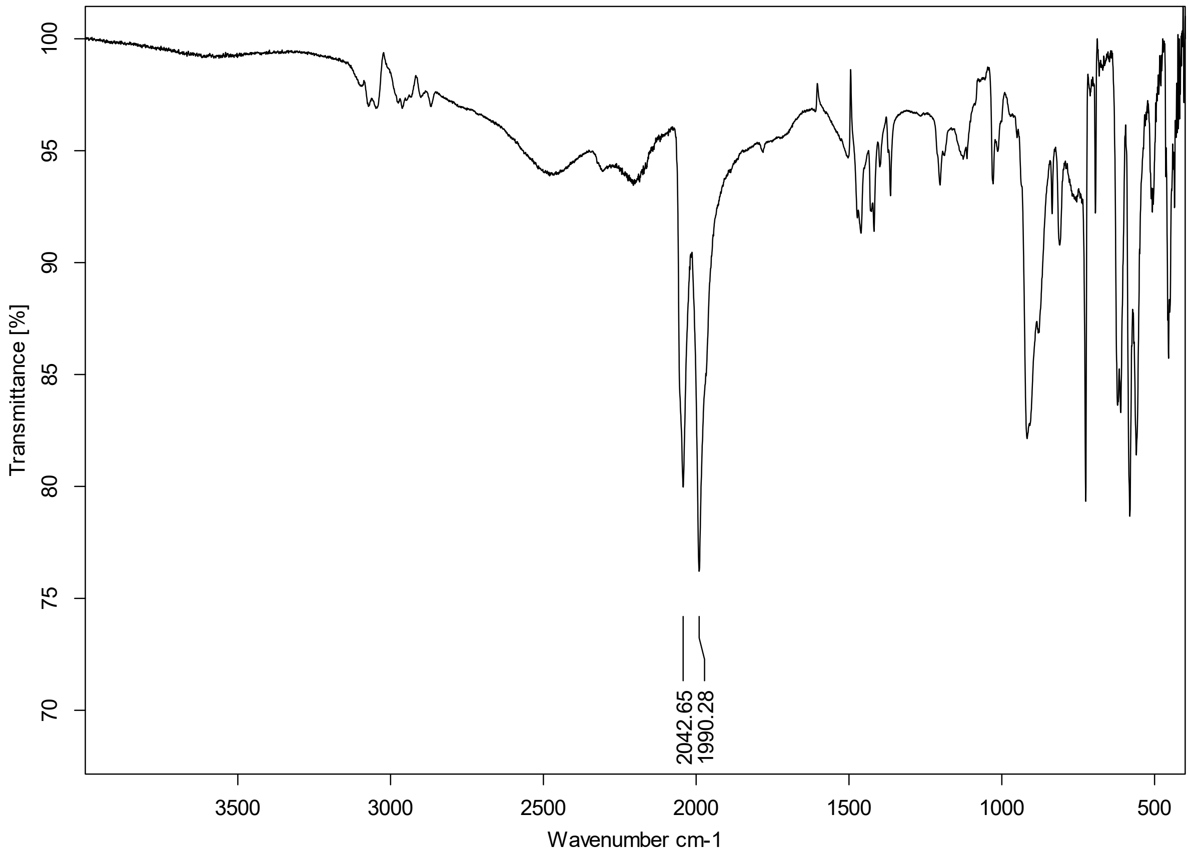

Figure S4: ATR IR spectrum of **2**-Cl.

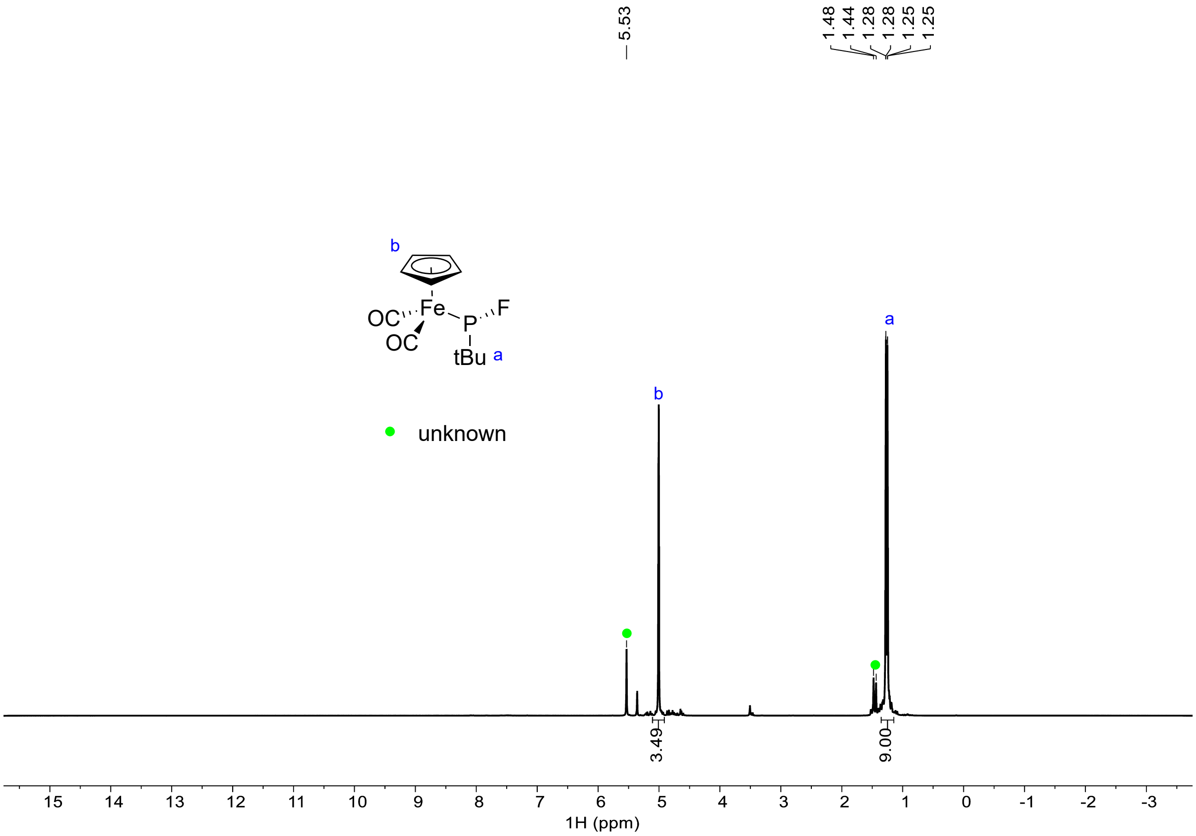

Figure S5: ^{1}H NMR spectrum of **2**-F in CD_2Cl_2 at 25 °C, recorded at 400 MHz.

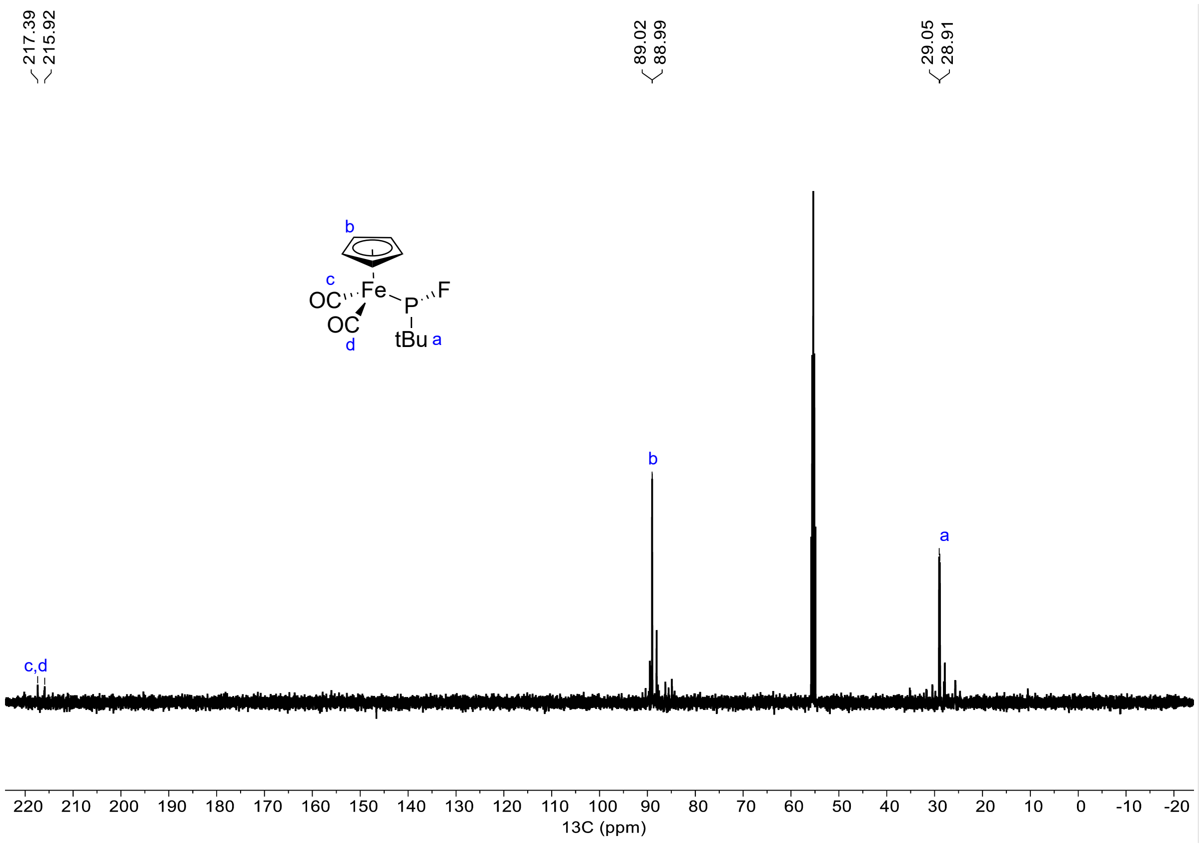

Figure S6: ^{13}C NMR spectrum of **2**-F in CD_2Cl_2 at 25 °C, recorded at 126 MHz.

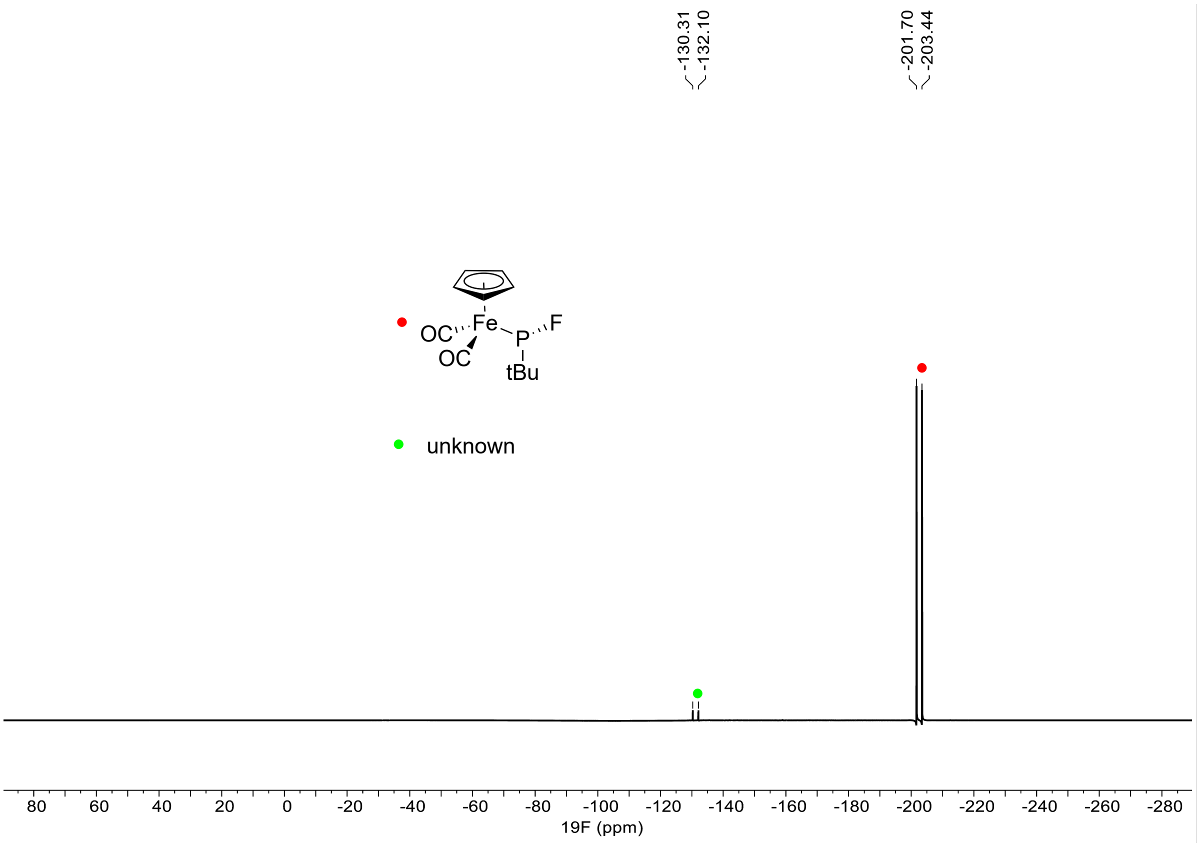

Figure S7: $^{19}F\{^{1}H\}$ NMR spectrum of **2**-F in CD_2Cl_2 at 25 °C, recorded at 471 MHz.

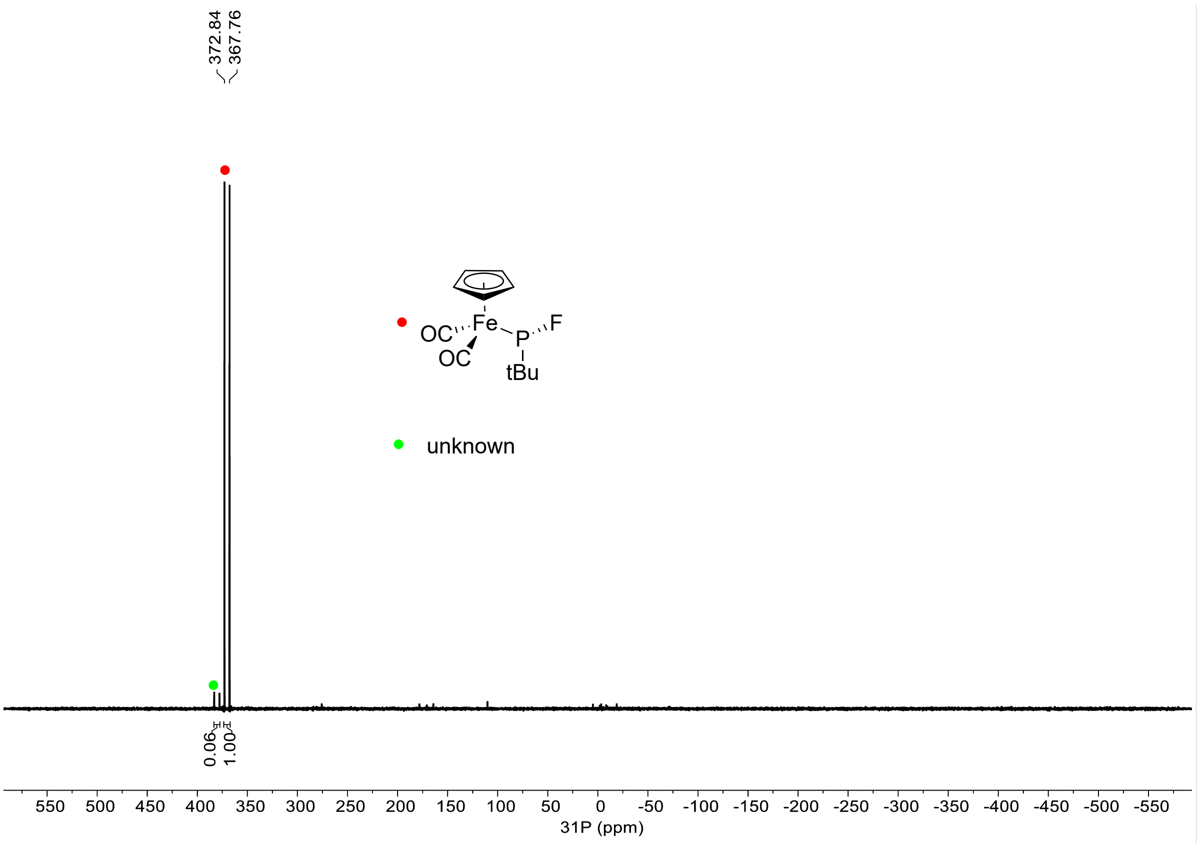

Figure S8: $^{31}P\{^{1}H\}$ NMR spectrum of **2**-F in CD_2Cl_2 at 25 °C, recorded at 162 MHz.

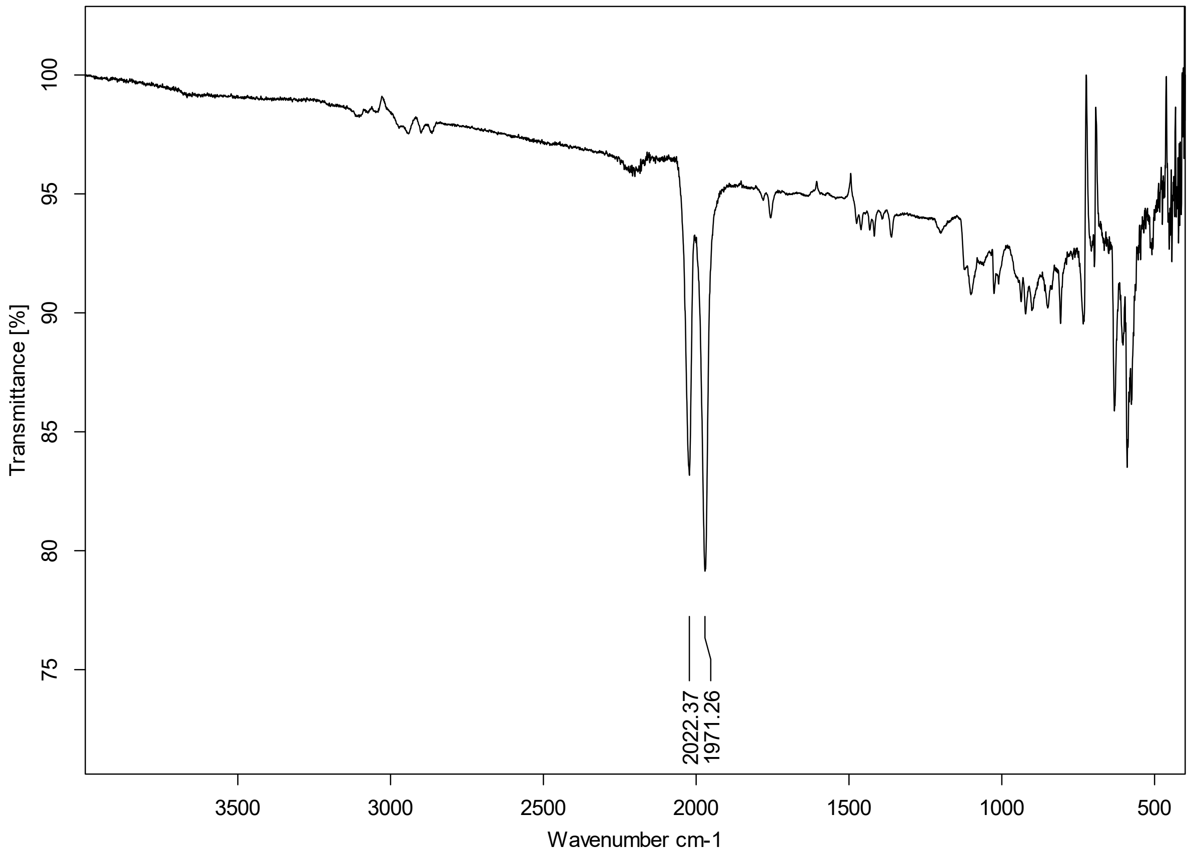

Figure S9: ATR IR spectrum of **2**-F.

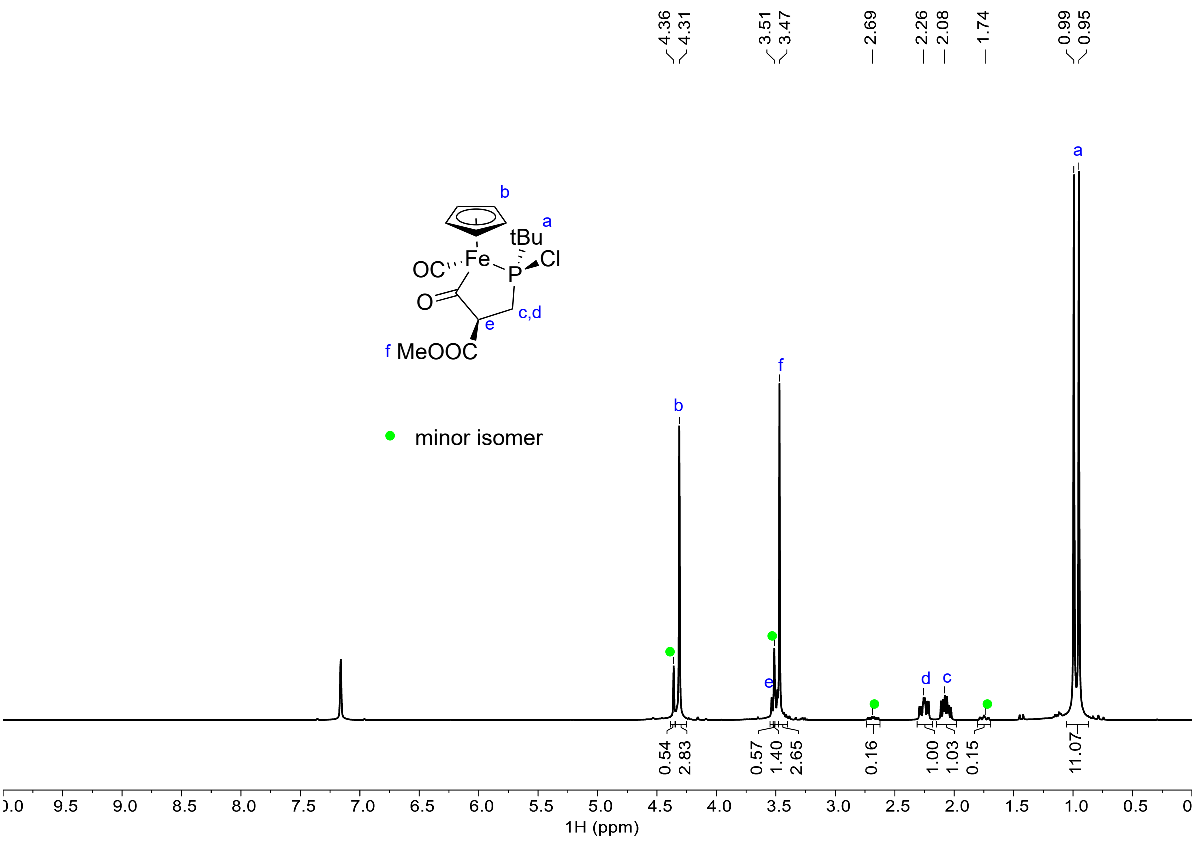

Figure S10: ^{1}H NMR spectrum of **3** in C_6D_6 at 25 °C, recorded at 400 MHz.

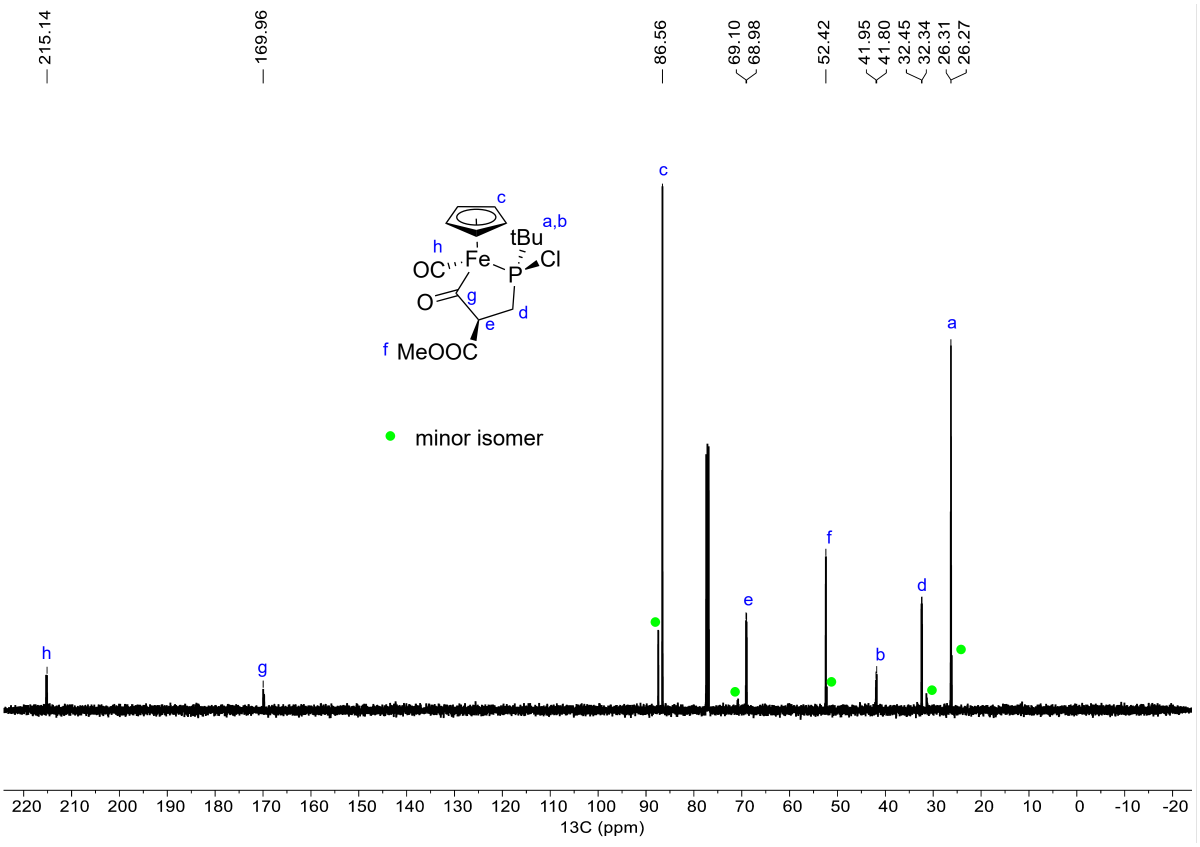

Figure S11: ^{13}C NMR spectrum of **3** in $CDCl_3$ at 25 °C, recorded at 126 MHz.

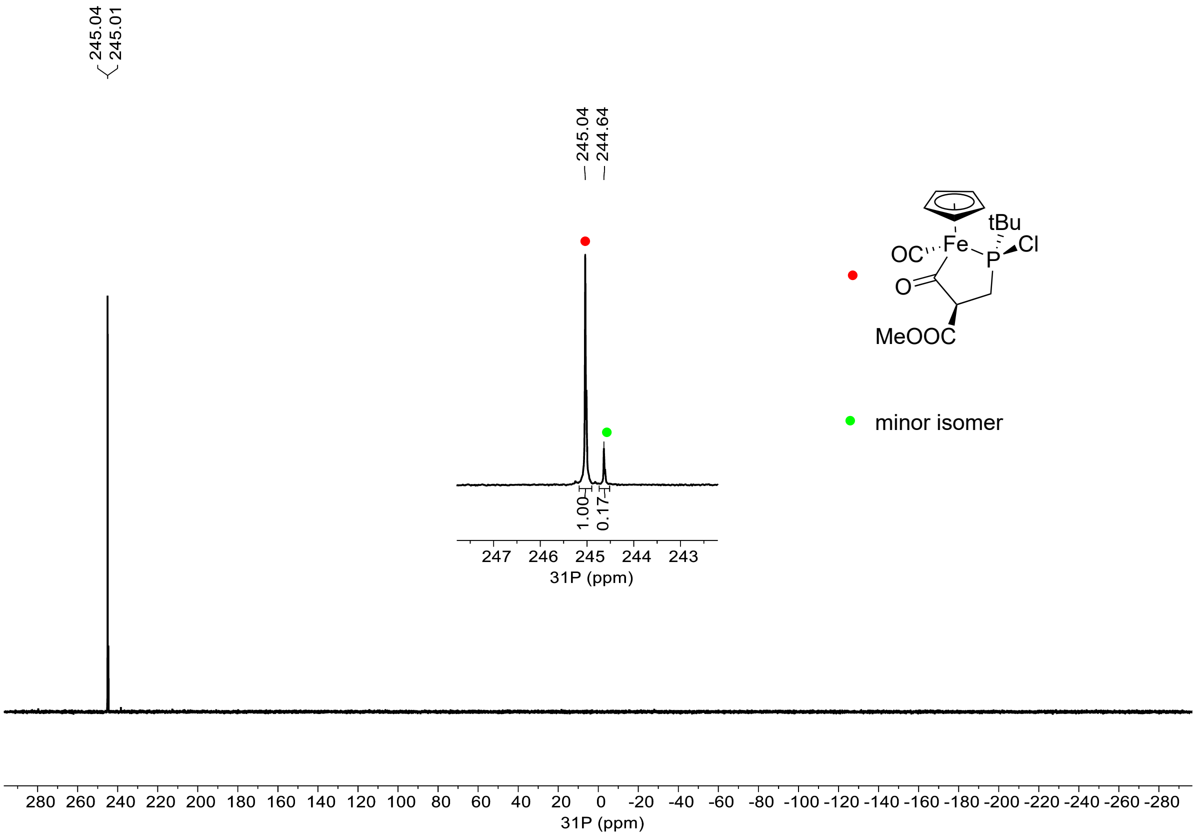

Figure S12: $^{31}P\{^{1}H\}$ NMR spectrum of **3** in C_6D_6 at 25 °C, recorded at 162 MHz.

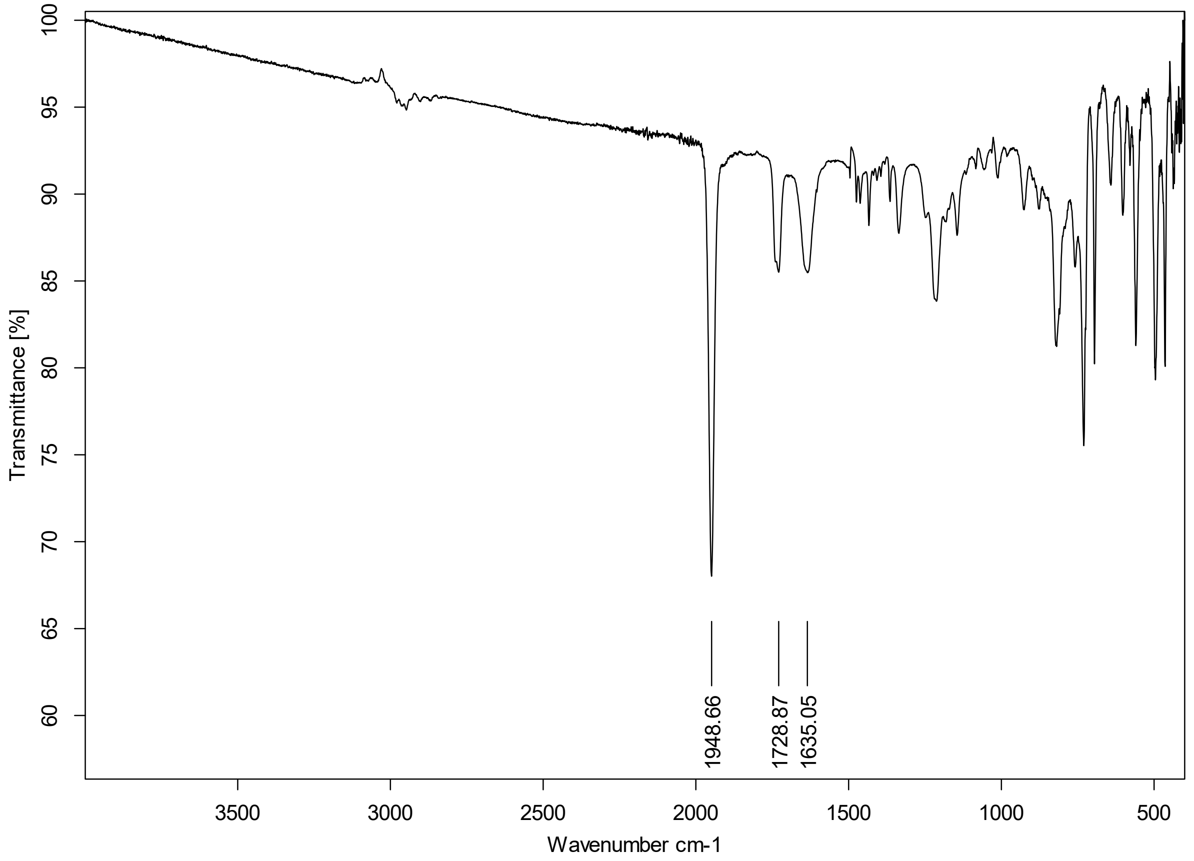

Figure S13: ATR IR spectrum of **3**.

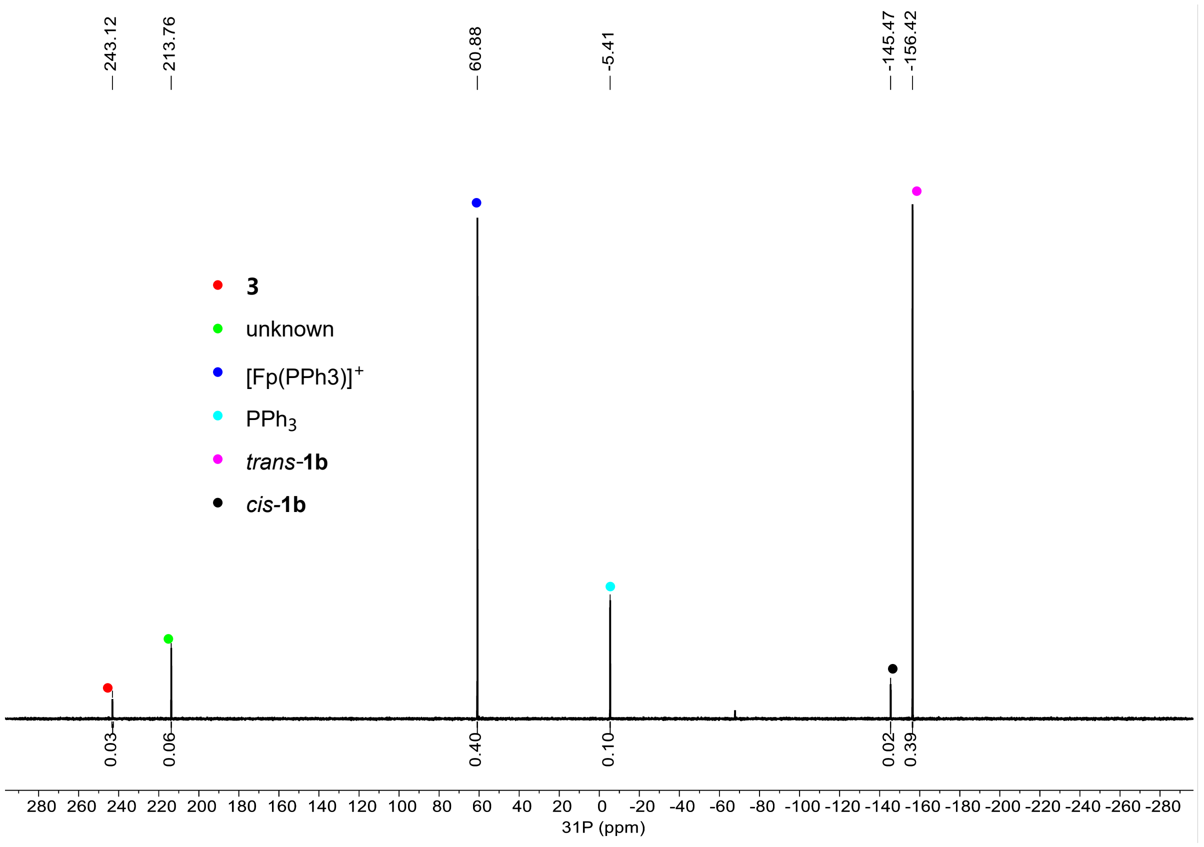

Figure S14: $^{31}P\{^{1}H\}$ NMR spectrum of the reaction mixture of **3**, PPh_3 and $NaBPh_4$ after stirring in DCM overnight at 23 °C, recorded at 162 MHz.

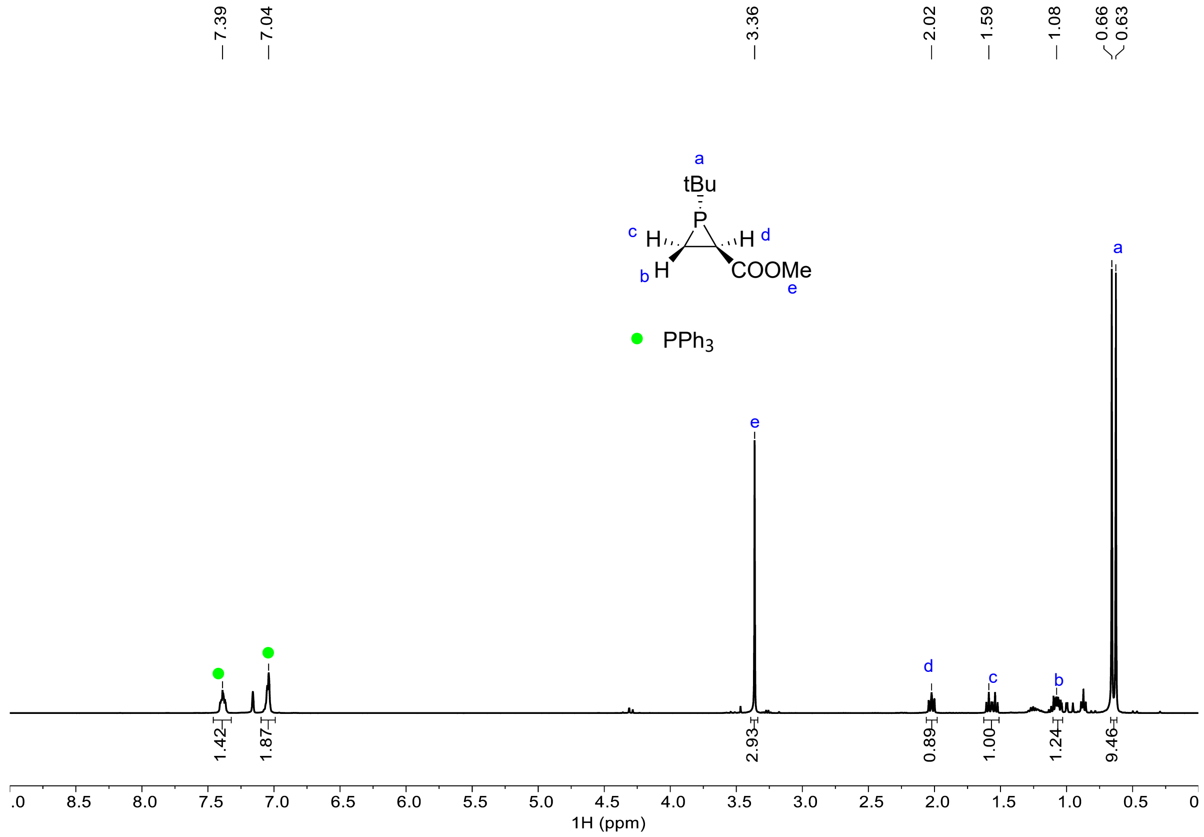

Figure S15: ^{1}H NMR spectrum of the crude **1b** isolated from the reaction mixture, recorded at 400 MHz.

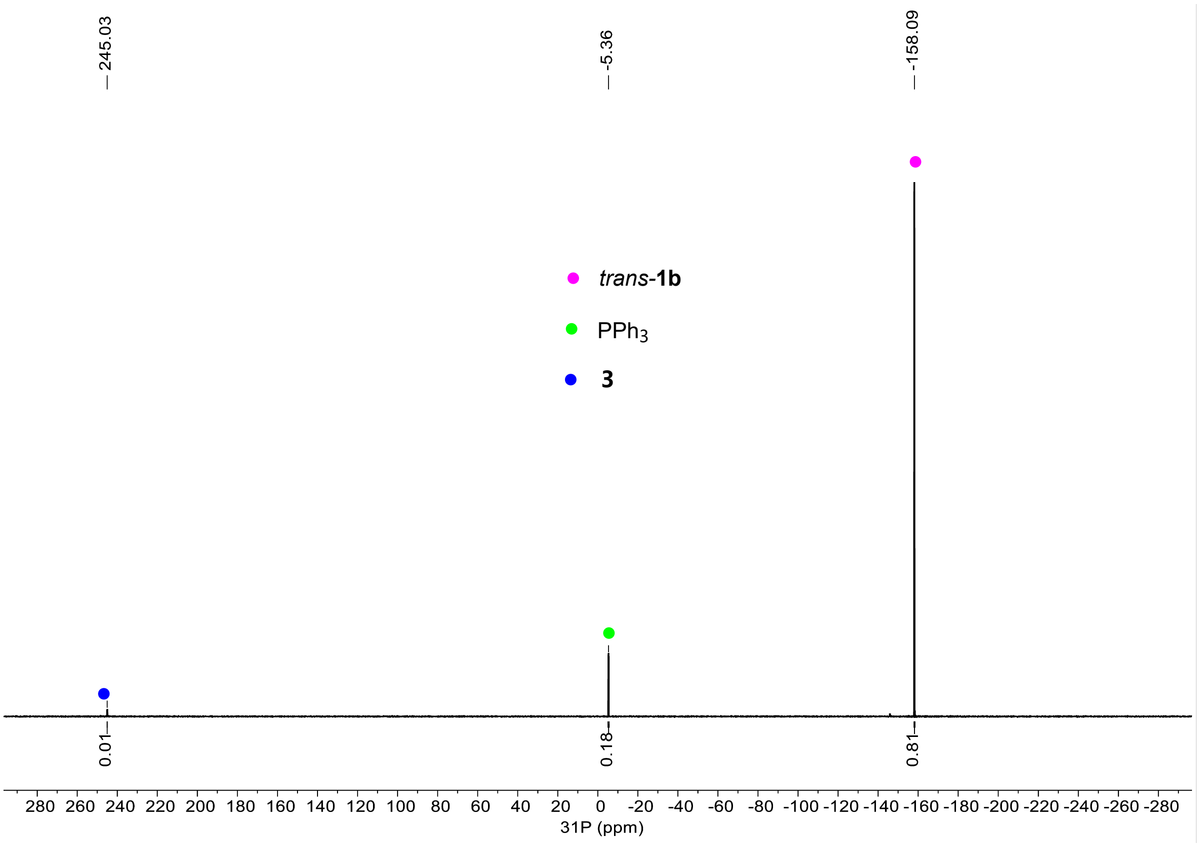

Figure S16: $^{31}P\{^{1}H\}$ NMR spectrum of the crude **1b** isolated from the reaction mixture, recorded at 162 MHz.

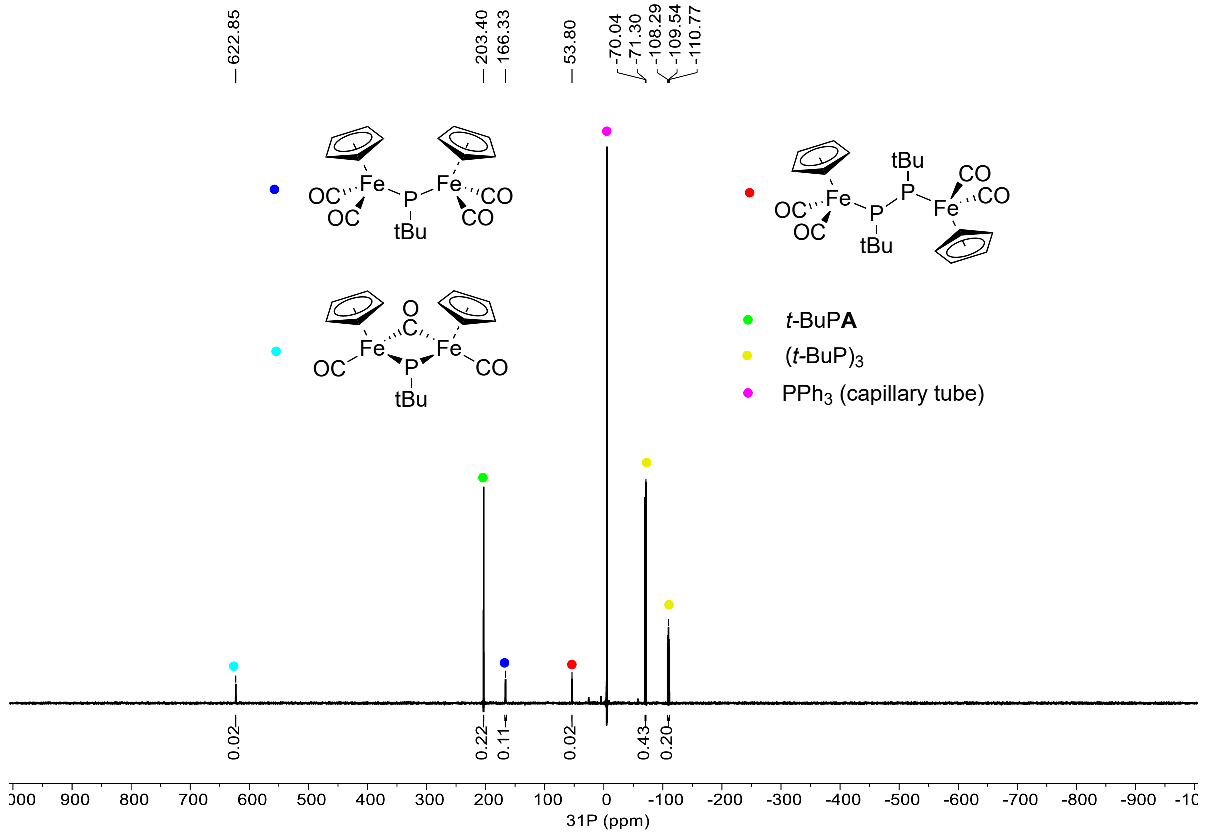

Figure S17: $^{31}P\{^{1}H\}$ NMR spectrum of the reaction mixture of Fp_2 and ^{t}BuP**A** in THF after 1 h at 80 °C, recorded at 162 MHz.

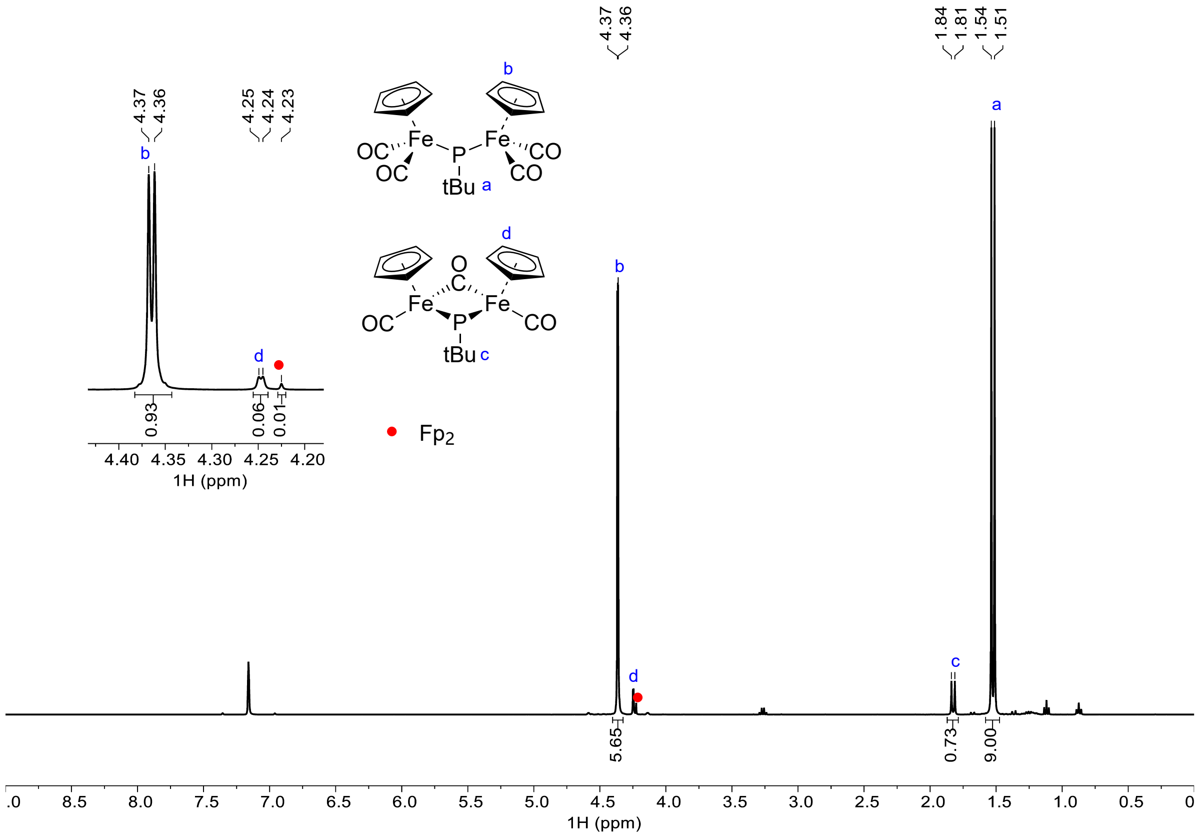

Figure S18: ^{1}H NMR spectrum of **4** isolated by recrystallization twice, recorded at 400 MHz.

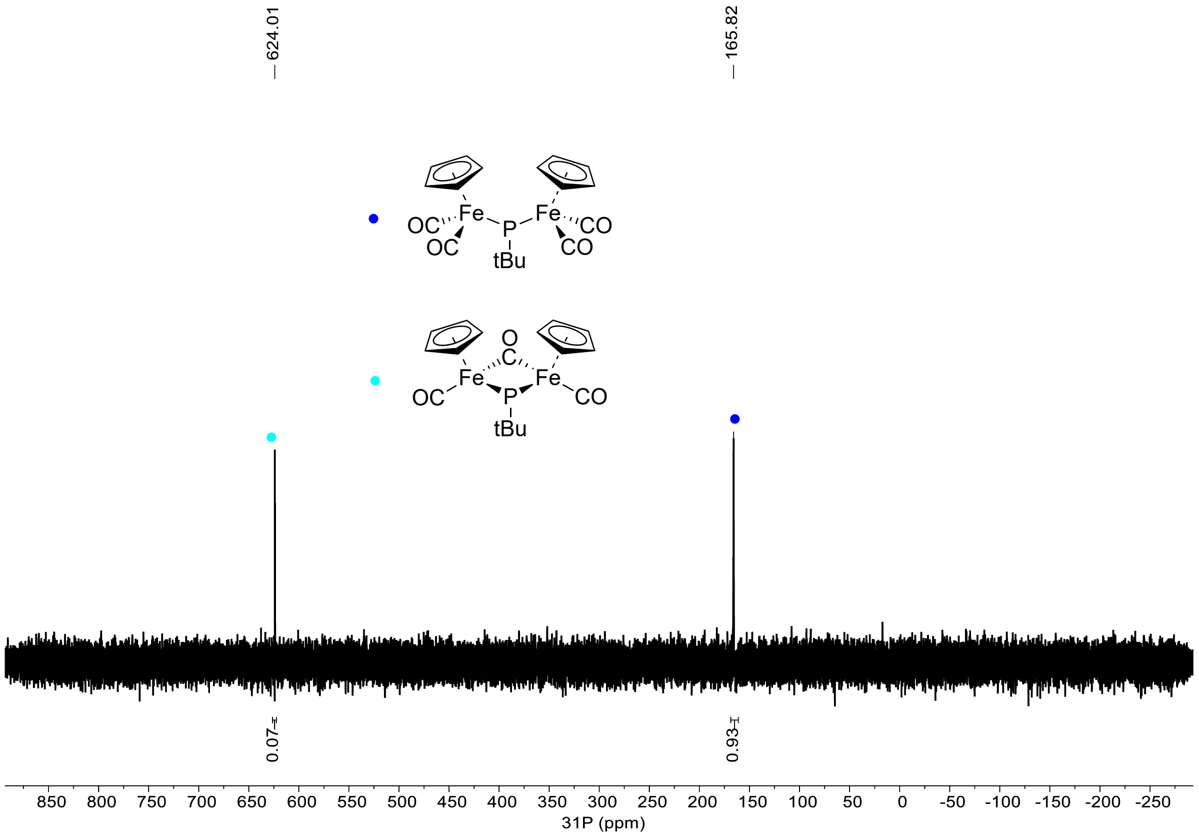

Figure S19: $^{31}P\{^{1}H\}$ NMR spectrum of **4** isolated by recrystallization twice, recorded at 162 MHz.

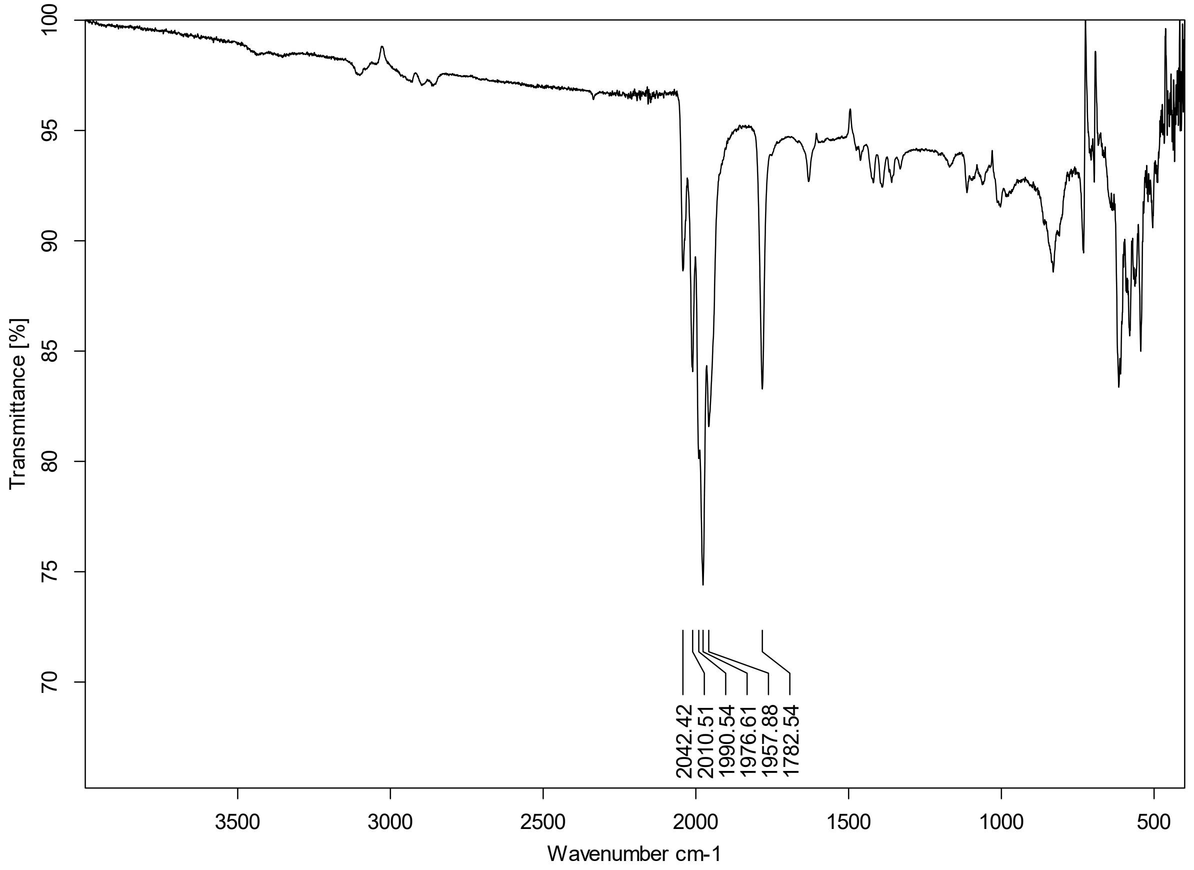

Figure S20: ATR IR spectrum of **4** isolated by recrystallization twice, with **5** as impurities.

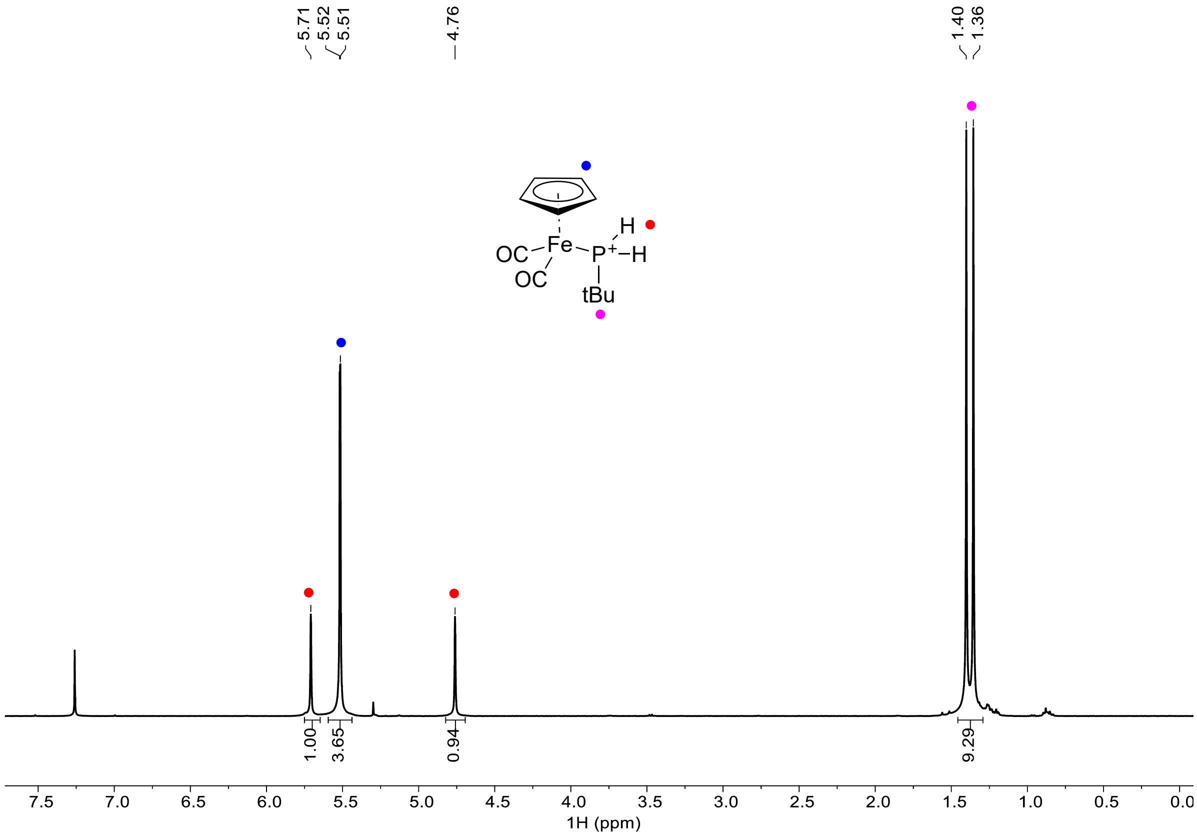

Figure S21: ^{1}H NMR spectrum of [Fp(^{t}BuPH$_2$)][BF$_4$] in CDCl$_3$, recorded at 400 MHz.

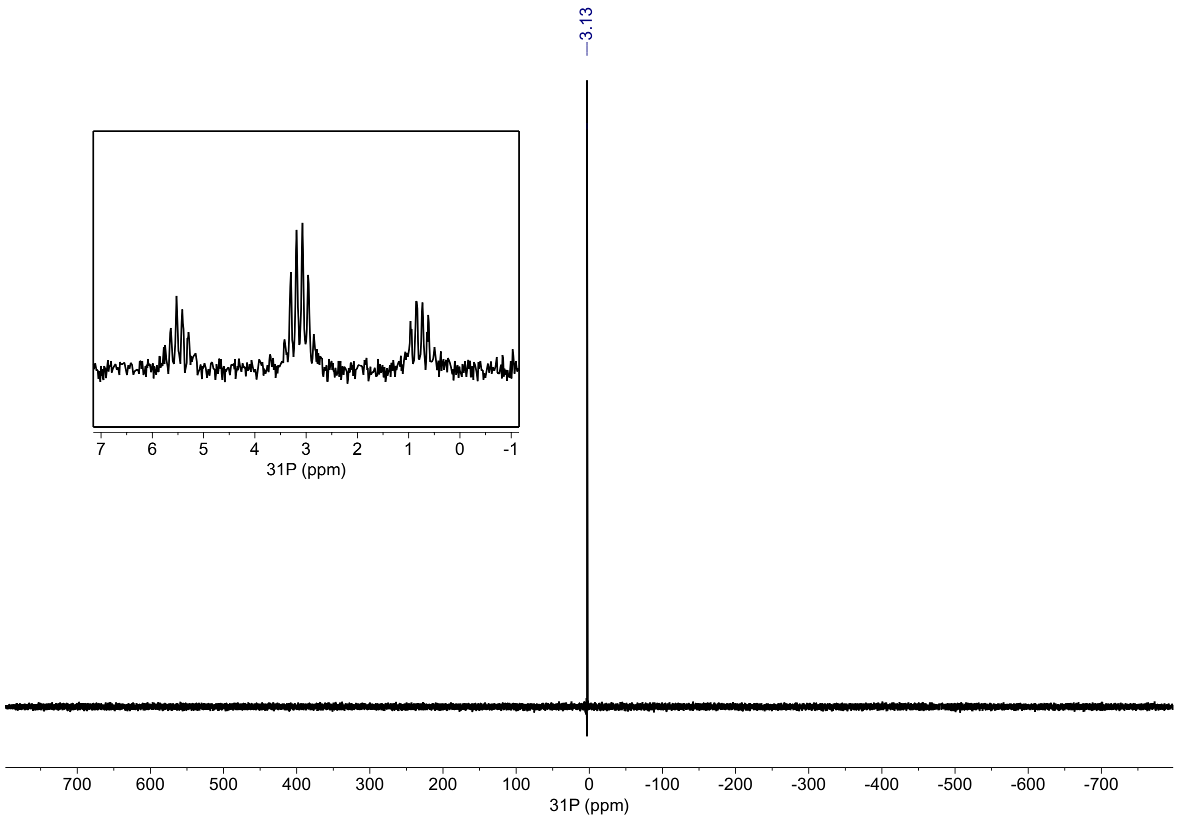

Figure S22: $^{31}P\{^{1}H\}$ NMR spectrum of $[Fp(^{t}BuPH_2)][BF_4]$ in $CDCl_3$ (inset: ^{31}P NMR spectrum), recorded at 400 MHz.

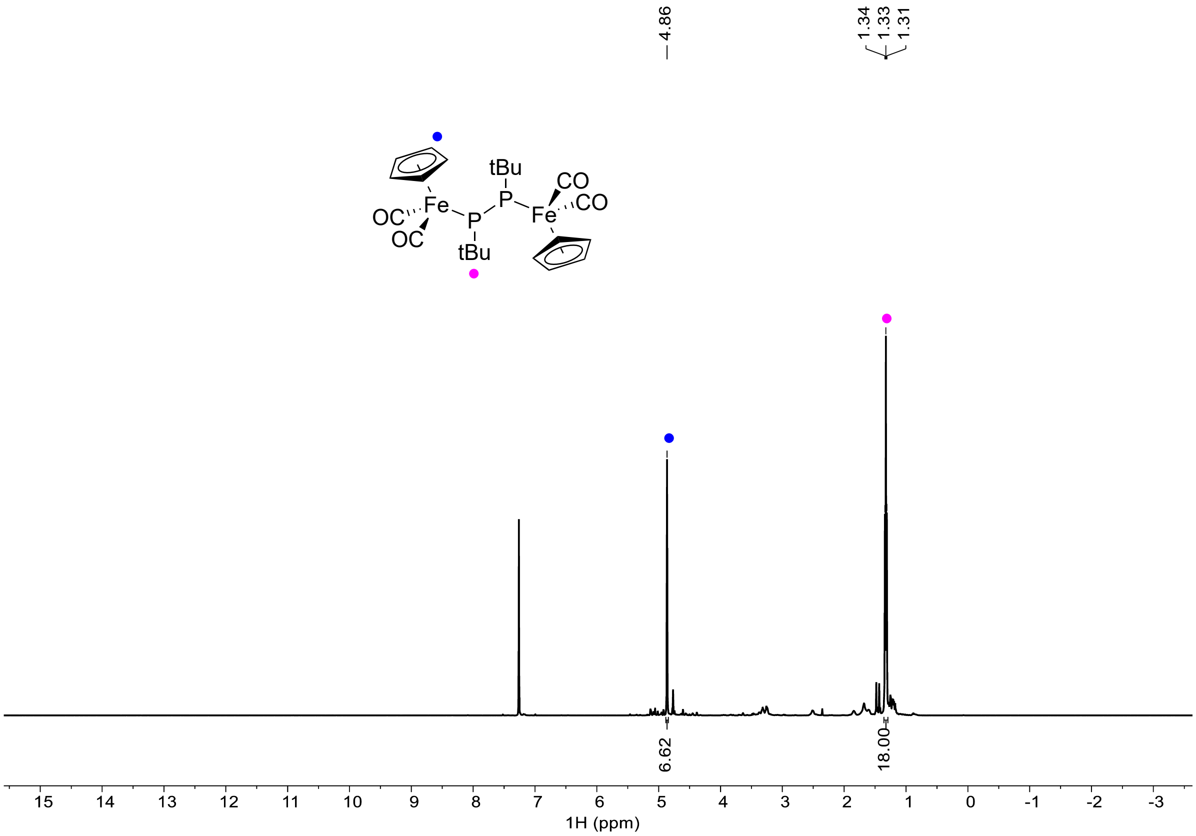

Figure S23: ^{1}H NMR spectrum of **6** in $CDCl_3$, recorded at 400 MHz.

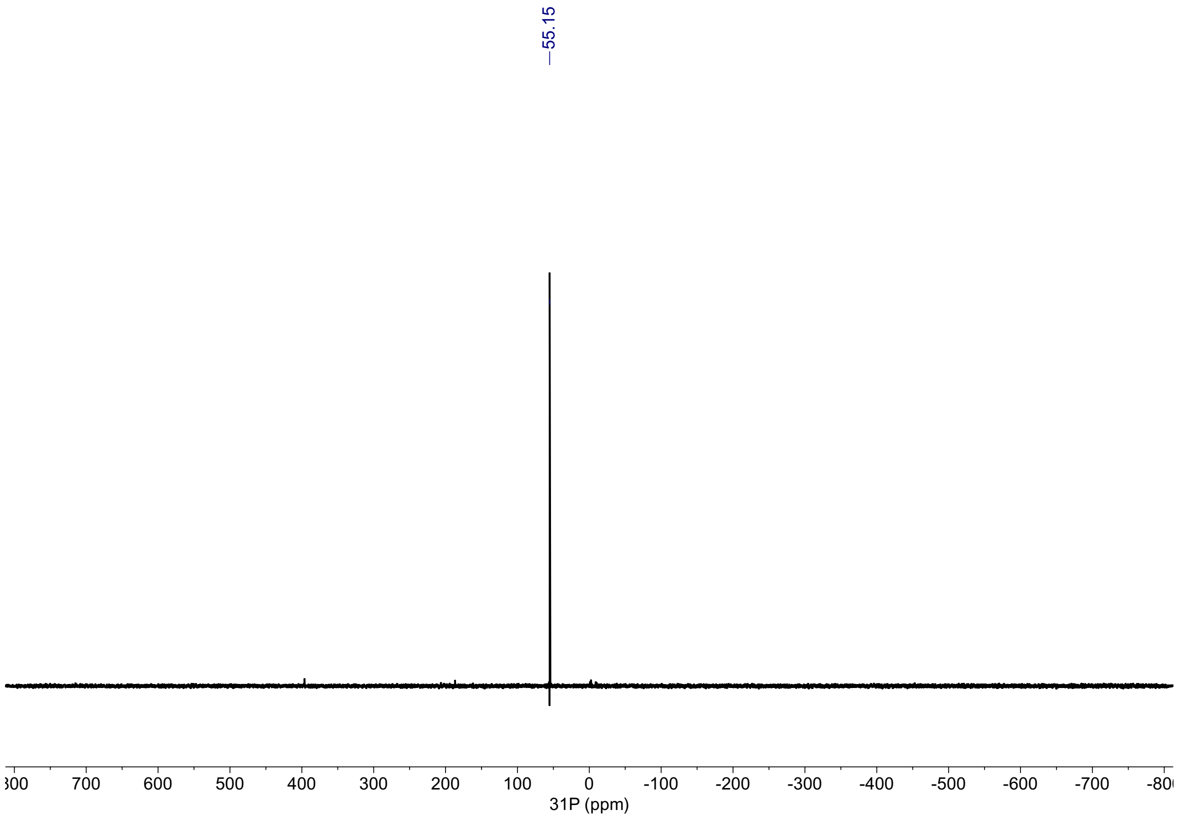

Figure S24: $^{31}P\{^{1}H\}$ NMR spectrum of **6** in $CDCl_3$, recorded at 162 MHz.

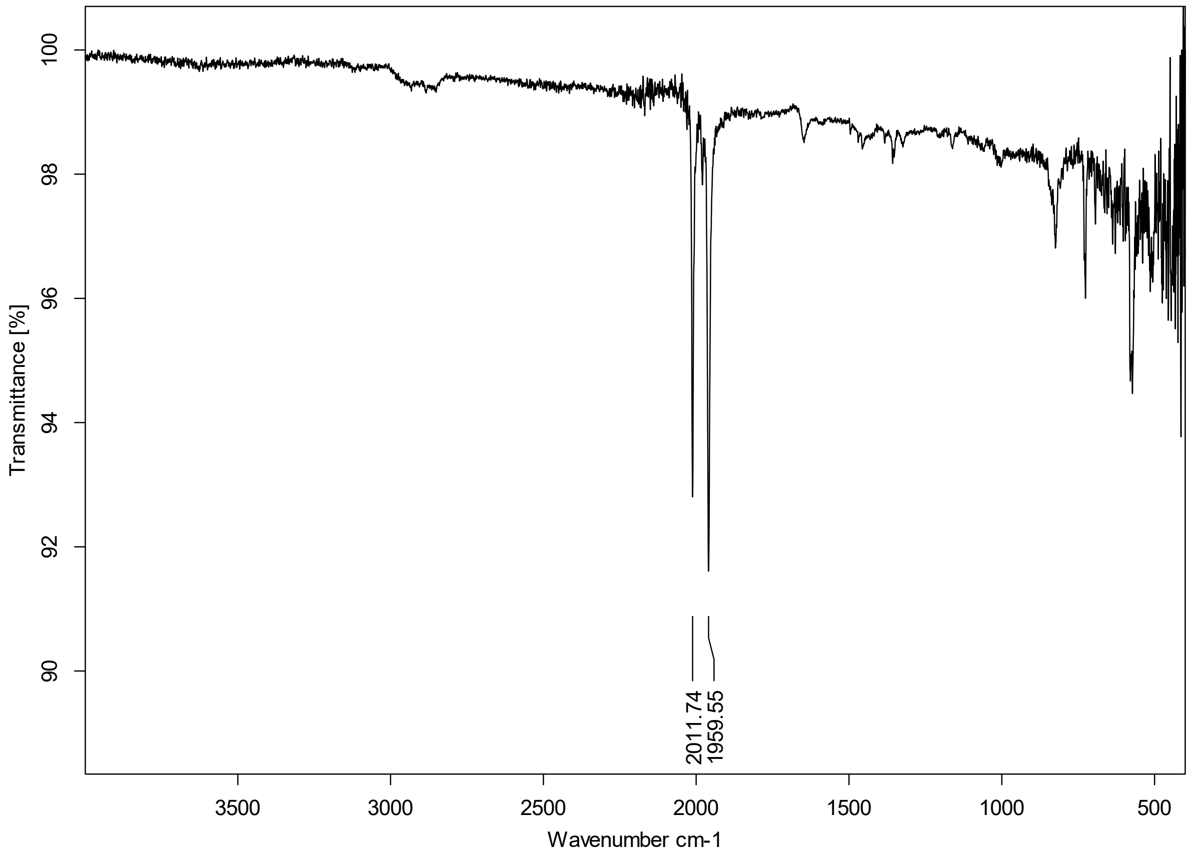

Figure S25: ATR IR spectrum of **6**.

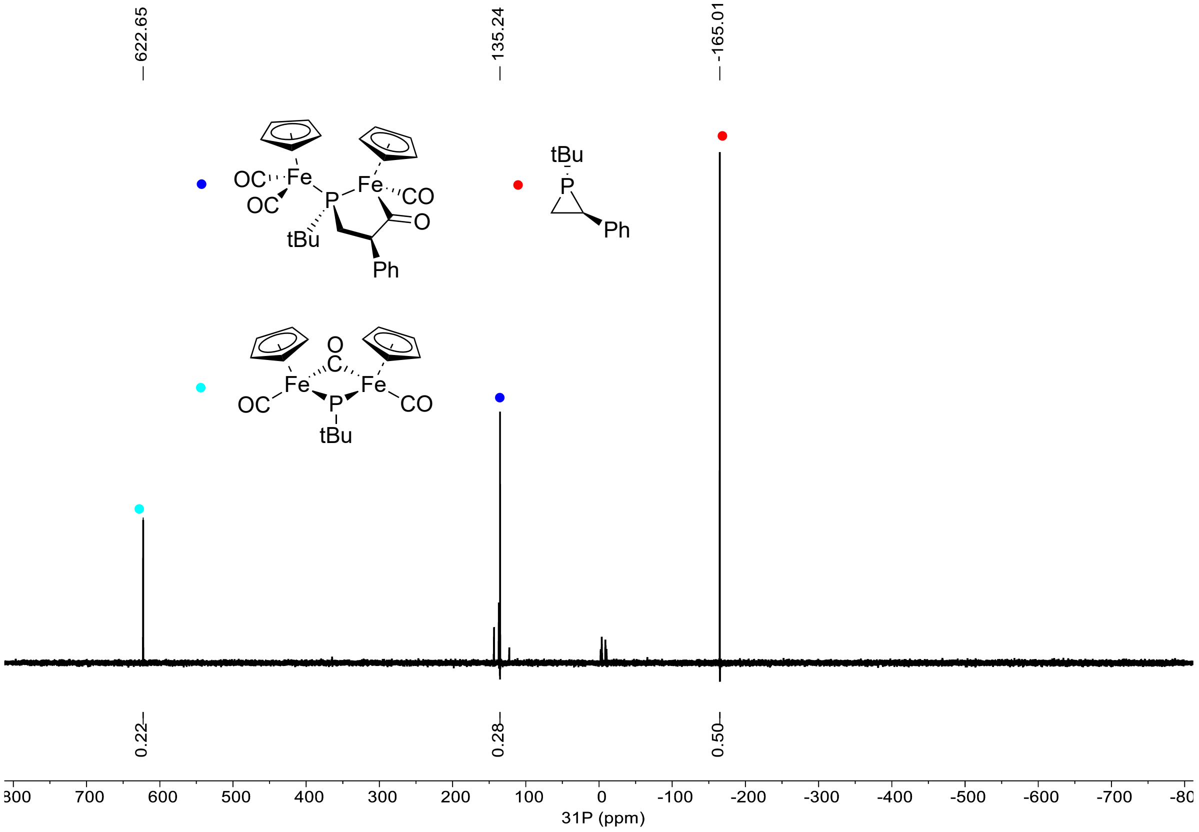

Figure S26: $^{31}P\{^{1}H\}$ NMR spectrum of the reaction mixture of **4** and styrene in THF after 0.5 h at 80 °C, recorded at 162 MHz.

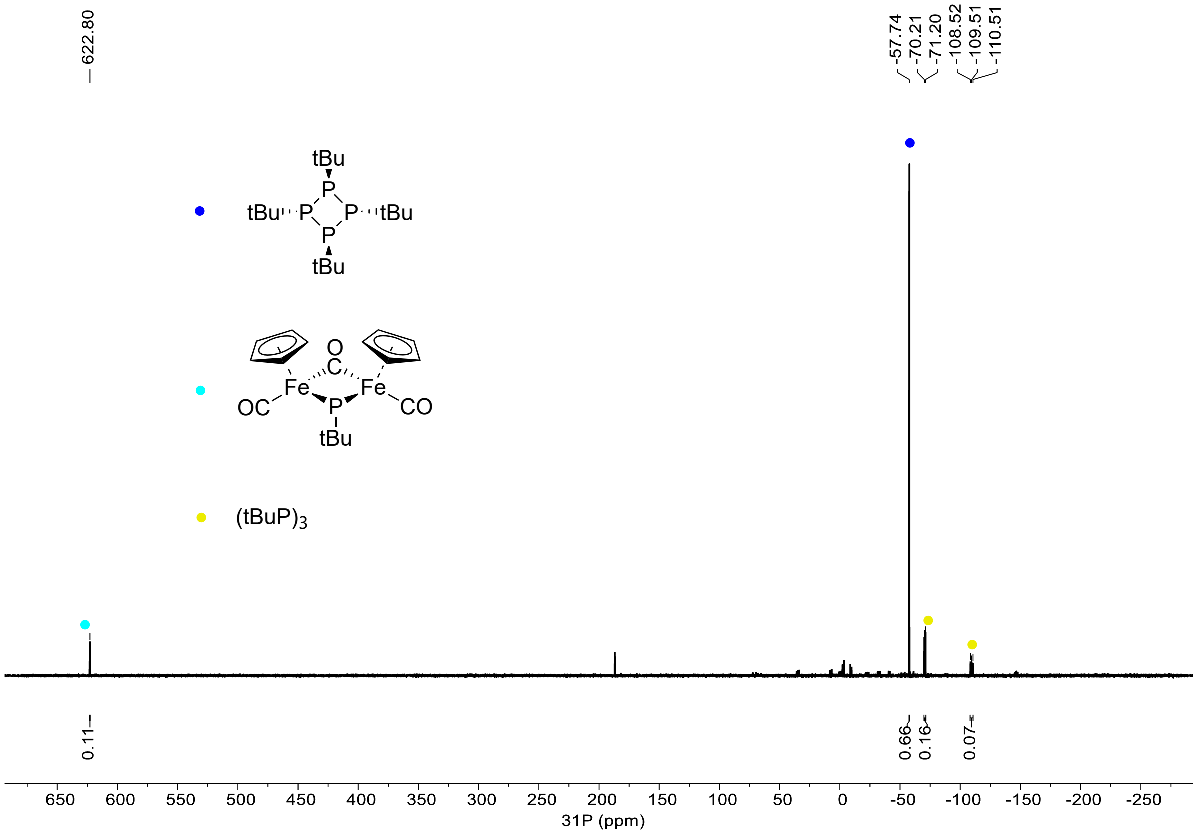

Figure S27: $^{31}P\{^{1}H\}$ NMR spectrum of the reaction mixture of **6** and styrene in THF after 0.5 h at 80 °C, recorded at 203 MHz.

S1.3 Catalyst screening

A list of compounds tested as catalyst for styrene phosphiranation is summarized in Table S1. A typical setup involves heating a solution of ^{t}BuP**A** (26 mg, 0.1 mmol, 1.0 equiv), styrene (104 mg, 1.0 mmol, 10 equiv) and the catalyst in THF (1 mL) in a J-Young tube covered by aluminum foil at 80 °C for 16 h. After cooling, the tube was analyzed by $^{31}P\{^{1}H\}$ NMR spectroscopy. The yield of **1a** was estimated from the ratio of integration of the **1a** signal to all phosphorus signals.

Table S1: Catalyst screened for styrene phosphiranation

entry	catalyst	catalyst loading	**1a** yield (%)
1	$[Fp(THF)][BF_4]/1.5\ [Me_4N]F$	10 mol%	91(73[a])
2	$[Fp(THF)][BF_4]/1.5\ [n Bu_4N]Cl$	10 mol%	82
3	$[Fp(THF)][BF_4]$	10 mol%	4
4	TMAF	10 mol%	5
5	**2**-F	10 mol%	90(75[a])
6	**2**-F[b]	10 mol%	90
7	**2**-Cl	10 mol%	72
8	FpCl	10 mol%	80
9	FpI	10 mol%	13
10	FpOTf	10 mol%	4
11	$FpPPh_2$	10 mol%	90
12	$FpPCy_2$	10 mol%	92(76[a])
13	Fp_2	5 mol%	99
14	Fp_2	2 mol%	99(78[a])
15	Fp_2	0.5 mol%	98[c]
16	Fp_2[b]	5 mol%	99
16	$Co_2(CO)_8$	5 mol%	22
17	$Fe_2(CO)_9$	5 mol%	63
18	$Mn_2(CO)_{10}$	5 mol%	0
19	$[CpMo(CO)_3]_2$	5 mol%	8

[a] Isolated yield. [b] With 20 mol % of TEMPO added. [c] Within 6 h.

S1.4 Synthesis of phosphiranes

S1.4.1 1-(*Tert*-butyl)-2-phenylphosphirane, 1a

A solution of ^{t}BuP**A** (1.06 g, 4.0 mmol, 1.0 equiv), Fp_2 (28 mg, 0.08 mmol, 2 mol%) and styrene (4.16 g, 40 mmol, 10 equiv) in THF (30 mL) was heated at 80 °C with stirring in a sealed Schlenk tube (100 mL) covered by aluminum foil for 16 h. After cooling to room temperature, the solution was concentrated to ca. 5 mL, and 30 mL of pentane was added, resulting in a white precipitate. The suspension was filtered through Celite in a frit (30 mL, medium porosity), and volatile materials were removed from the filtrate under reduced pressure. The residue was taken up in Et_2O (30 mL) and passed through an activated charcoal plug (ca. 5 cm thick) in a frit (30 mL, medium porosity). Additional Et_2O (3×20 mL) was used to wash the charcoal plug, and volatile materials were removed from the combined filtrate under reduced pressure. The red residue was transferred to a vacuum distillation apparatus with the help of ca. 6 mL Et_2O. The apparatus was then brought out of the glovebox and cycled onto the Schlenk line and Et_2O was removed in vacuo. The remaining material was vacuum distilled under static vacuum at 70–80 °C to the receiving side (–78 °C) to yield **1a** as a pale yellow liquid (598 mg, 3.1 mmol, 78%). All characterization data matched previously reported values.[S10]

S1.4.2 Methyl 1-(*tert*-butyl)phosphirane-2-carboxylate, 1b

A solution of ^{t}BuP**A** (1.06 g, 4.0 mmol, 1.0 equiv), **2**-Cl (121 mg, 0.40 mmol, 10 mol%) and methyl acrylate (3.44 g, 40 mmol, 10 equiv) in THF (30 mL) was heated at 80 °C with stirring in a sealed Schlenk tube (100 mL) covered by aluminum foil for 16 h. After cooling to room temperature, volatile materials were removed under reduced pressure. The residue was taken up in Et_2O (20 mL) and passed through an activated charcoal plug

(ca. 5 cm thick) in a frit (30 mL, medium porosity). Additional Et_2O (3×15 mL) was used to wash the charcoal plug, and volatile materials were removed from the combined filtrate under reduced pressure. The red residue was transferred to a vacuum distillation apparatus with the help of ca. 6 mL Et_2O. The apparatus was then brought out of the glovebox and cycled onto the Schlenk line and Et_2O was removed in vacuo. The remaining material was vacuum distilled under static vacuum at 70–80 °C to the receiving side (–78 °C) to yield **1b** as a pale yellow liquid (286 mg, 1.6 mmol, 41%). ^{1}H NMR (500 MHz, chloroform-*d*, Figure S28) δ 3.66 (s, 3H), 2.06 (td, J = 8.0, 1.2 Hz, 1H), 1.48 – 1.38 (m, 2H), 0.96 (d, J = 12.5 Hz, 9H) ppm. ^{13}C NMR (126 MHz, chloroform-*d*, Figure S29) δ 174.28 (d, J = 7.8 Hz), 51.94, 28.28 (d, J = 16.1 Hz), 26.37 (d, J = 33.7 Hz), 19.11 (d, J = 42.6 Hz), 11.00 (d, J = 49.3 Hz) ppm. $^{31}P\{^{1}H\}$ NMR (203 MHz, chloroform-*d*, Figure S30) δ –156.09 (s) ppm. No satisfactory elemental analysis data could be obtained due to the compound slowly decomposing at room temperature.

S1.4.3 1-(*Tert*-butyl)phosphirane-2-carbonitrile, 1c

A solution of ^{t}BuP**A** (800 mg, 3.0 mmol, 1.0 equiv) and acrylonitrile (796 mg, 15 mmol, 5.0 equiv) in THF (20 mL) was heated at 80 °C with stirring in a sealed Schlenk tube covered by aluminum foil for 16 h. After cooling to room temperature, volatile materials were removed under reduced pressure. The residue was taken up in Et_2O (20 mL) and passed through an activated charcoal plug (ca. 5 cm thick) in a frit (30 mL, medium porosity). Additional Et_2O (3×15 mL) was used to wash the charcoal plug, and volatile materials were removed from the combined filtrate under reduced pressure to yield **1c** as a colorless liquid (350 mg, 2.5 mmol, 83%, 91:9 dr). Anal. Calcd for $C_7H_{12}NP$: C, 59.56; H, 8.57; N, 9.92. Found: C, 57.87; H, 8.43; N, 9.89. **1c**-*trans* (major isomer): ^{1}H NMR (500 MHz, chloroform-*d*, Figure S31) δ 1.68 (dd, J = 10.3, 7.4 Hz, 1H), 1.52 (ddd, J =

10.3, 8.4, 4.1 Hz, 1H), 1.27 (ddd, J = 19.8, 8.4, 7.3 Hz, 1H), 0.98 (d, J = 12.9 Hz, 9H) ppm. ^{13}C NMR (126 MHz, chloroform-d, Figure S32) δ 121.43 (d, J = 11.5 Hz), 27.81 (d, J = 16.0 Hz), 26.55 (d, J = 34.1 Hz), 10.76 (d, J = 47.1 Hz), 0.83 (d, J = 40.3 Hz) ppm. ^{31}P{^{1}H} NMR (203 MHz, chloroform-d, Figure S33) δ –162.50 (s) ppm. **1c**-*cis* (minor isomer): ^{1}H NMR (500 MHz, chloroform-d, Figure S31) δ 1.71 (td, J = 8.0, 4.9 Hz, 1H), 1.58 (ddd, J = 13.5, 10.1, 8.0 Hz, 1H), 1.36 (ddd, J = 18.5, 10.1, 8.3 Hz, 1H), 1.20 (d, J = 13.5 Hz, 9H) ppm. ^{13}C NMR (126 MHz, chloroform-d, Figure S32) δ 28.54 (d, J = 16.7 Hz), 10.84 (d, J = 46.2 Hz) ppm. ^{31}P{^{1}H} NMR (203 MHz, chloroform-d, Figure S33) δ –159.60 (s) ppm.

S1.4.4 2-([1,1'-Biphenyl]-4-yl)-1-(*tert*-butyl)phosphirane, 1d

A solution of ^{t}BuP**A** (266 mg, 1.0 mmol, 1.0 equiv), Fp_2 (7 mg, 0.02 mmol, 2 mol%) and 4-vinylbiphenyl (180 mg, 1.0 mmol, 1.0 equiv) in THF (15 mL) was heated at 80 °C with stirring in a sealed Schlenk tube covered by aluminum foil for 16 h. After cooling to room temperature, volatile materials were removed under reduced pressure. The residue was taken up in Et_2O (20 mL) and passed through an activated charcoal plug (ca. 5 cm thick) in a frit (15 mL, medium porosity). Additional Et_2O (6×10 mL) was used to wash the charcoal plug, and volatile materials were removed from the combined filtrate under reduced pressure. The crude product was recrystallized in minimal pentane (ca. 1.5 mL) at –35 °C, collected by decanting, rinsing with minimal cold pentane and drying under vacuum to yield **1d** as a white crystalline solid (197 mg, 0.73 mmol, 73%). Anal. Calcd for $C_{18}H_{21}P$: C, 80.57; H, 7.89. Found: C, 79.65; H, 7.83. ^{1}H NMR (400 MHz, chloroform-d, Figure S34) δ 7.58 (dd, J = 8.3, 1.4 Hz, 2H), 7.49 (d, J = 8.3 Hz, 2H), 7.44 (t, J = 7.7 Hz, 2H), 7.37 – 7.30 (m, 1H), 7.14 (d, J = 8.3 Hz, 2H), 2.53 (ddd, J = 10.1, 7.6, 2.1 Hz, 1H), 1.62 (ddd, J = 10.1, 8.0, 1.8 Hz, 1H), 1.33 (dt, J = 18.7, 7.9 Hz, 1H), 1.07 (d, J =

12.1 Hz, 9H) ppm.^{13}C NMR (126 MHz, chloroform-*d*, Figure S35) δ 142.43 (d, J = 8.5 Hz), 141.09, 138.34, 128.71, 127.09, 126.95, 126.93, 126.63 (d, J = 5.1 Hz), 28.76 (d, J = 15.6 Hz), 26.41 (d, J = 33.7 Hz), 22.62 (d, J = 41.0 Hz), 13.53 (d, J = 45.6 Hz) ppm. ^{31}P{^{1}H} NMR (162 MHz, chloroform-*d*, Figure S36) δ –164.78f (s) ppm.

S1.4.5 1-(*Tert*-butyl)-2-(phenylsulfonyl)phosphirane, 1e

A solution of ^{t}BuP**A** (400 mg, 1.5 mmol, 1.0 equiv), Fp_2 (10 mg, 0.03 mmol, 2 mol%) and phenyl vinyl sulfone (180 mg, 1.0 mmol, 1.0 equiv) in THF (15 mL) was heated at 80 °C with stirring in a sealed Schlenk tube covered by aluminum foil for 16 h. After cooling to room temperature, volatile materials were removed under reduced pressure. The residue was taken up in Et_2O (20 mL) and passed through an activated charcoal plug (ca. 5 cm thick) in a frit (15 mL, medium porosity). Additional Et_2O (6×10 mL) was used to wash the charcoal plug, and volatile materials were removed from the combined filtrate under reduced pressure. The crude product was recrystallized in minimal pentane (ca. 1.5 mL) at –35 °C, collected by decanting, rinsing with minimal cold pentane and drying under vacuum to yield **1e** as a white crystalline solid (269 mg, 0.71 mmol, 71%). Anal. Calcd for $C_{12}H_{17}O_2PS$: C, 56.24; H, 6.69. Found: C, 55.79; H, 6.69. ^{1}H NMR (500 MHz, chloroform-*d*, Figure S37) δ 7.94 (d, J = 7.4 Hz, 1H), 7.63 (t, J = 7.4 Hz, 1H), 7.56 (t, J = 7.7 Hz, 2H), 2.78 (t, J = 8.5 Hz, 1H), 1.64 – 1.55 (m, 2H), 0.90 (d, J = 12.9 Hz, 9H) ppm. ^{13}C NMR (126 MHz, chloroform-*d*, Figure S38) δ 140.97, 133.29, 129.09, 127.81, 41.25 (d, J = 48.6 Hz), 28.09 (d, J = 16.2 Hz), 26.18 (d, J = 33.4 Hz), 10.55 (d, J = 46.8 Hz) ppm. ^{31}P{^{1}H} NMR (162 MHz, chloroform-*d*, Figure S39) δ –164.98 (s) ppm.

S1.4.6 (1-(*tert*-butyl)phosphiran-2-yl)diphenylphosphine oxide, 1f

A solution of ^{t}BuP**A** (266 mg, 1.0 mmol, 1.0 equiv), Fp_2 (7 mg, 0.02 mmol, 2 mol%) and vinyldiphenylphosphine oxide (217 mg, 0.95 mmol, 0.95 equiv) in THF (15 mL) was heated at 80 °C with stirring in a sealed Schlenk tube covered by aluminum foil for 72 h. After cooling to room temperature, volatile materials were removed under reduced pressure. The residue was taken up in Et_2O (25 mL) and passed through an activated charcoal plug (ca. 5 cm thick) in a frit (15 mL, medium porosity). Additional Et_2O (2×25 mL) was used to wash the charcoal plug, and volatile materials were removed from the combined filtrate under reduced pressure. The crude product was recrystallized in minimal Et_2O (ca. 2 mL) at –35 °C, collected by decanting, rinsing with minimal cold Et_2O and drying under vacuum to yield **1f** as a white crystalline solid (222 mg, 0.70 mmol, 70%). Anal. Calcd for $C_{12}H_{22}OP_2$: C, 68.35; H, 7.01. Found: C, 67.35; H, 7.06. ^{1}H NMR (500 MHz, chloroform-*d*, Figure S40) δ 7.92 – 7.72 (m, 4H), 7.58 – 7.37 (m, 6H), 1.70 (ddd, J = 10.6, 8.4, 4.7 Hz, 1H), 1.48 (tdd, J = 10.9, 7.6, 3.7 Hz, 1H), 1.28 (tt, J = 17.0, 8.1 Hz, 1H), 0.95 (d, J = 12.5 Hz, 9H) ppm. ^{13}C NMR (126 MHz, chloroform-*d*, Figure S41) δ 133.48 (dd, J = 103.6, 82.8 Hz), 131.68 (d, J = 2.6 Hz), 131.48 – 131.29 (m), 128.35 (dd, J = 19.8, 11.8 Hz), 28.34 (d, J = 16.2 Hz), 25.96 (d, J = 38.9 Hz), 15.16 (dd, J = 96.2, 49.5 Hz), 7.44 (d, J = 46.1 Hz) ppm. ^{31}P{^{1}H} NMR (162 MHz, chloroform-*d*, Figure S42) δ 30.88 (d, J = 28.6 Hz), –179.04 (d, J = 28.7 Hz) ppm.

S1.4.7 4-(1-(*tert*-butyl)phosphiran-2-yl)pyridine, 1g

The title compound was isolated as a borane adduct due to its instability on activated charcoal plug. A solution of ^{t}BuP**A** (266 mg, 1.0 mmol, 1.0 equiv), Fp_2 (7 mg, 0.02 mmol, 2 mol%) and 4-vinylpyridine (105 mg, 1.0 mmol, 1.0 equiv) in THF (15 mL) was heated

at 80 °C with stirring in a sealed Schlenk tube covered by aluminum foil for 16 h. After cooling to room temperature, volatile materials were removed under reduced pressure. The residue was dissolved in Et_2O (40 mL) and triphenylborane (242 mg, 1.0 mmol, 1.0 equiv) was added. The solution was stirred for 30 min and passed through an activated charcoal plug (ca. 5 cm thick) in a frit (15 mL, medium porosity). Additional Et_2O (2×50 mL) was used to wash the charcoal plug, and volatile materials were removed from the combined filtrate under reduced pressure. The crude product was recrystallized in minimal Et_2O (ca. 4 mL) at –35 °C, collected by decanting, rinsing with minimal cold Et_2O and drying under vacuum to yield **1g**-BPh_3 adduct as a white solid (358 mg, 0.82 mmol, 82%). Anal. Calcd for $C_{29}H_{31}BNP$: C, 80.01; H, 7.18; N, 3.22. Found: C, 79.56; H, 7.13; N, 3.36. ^{1}H NMR (400 MHz, chloroform-*d*, Figure S43) δ 8.29 (d, J = 6.9 Hz, 2H), 7.25 – 7.13 (m, 15H), 7.04 (d, J = 6.4 Hz, 2H), 2.49 – 2.43 (m, 1H), 1.82 (td, J = 9.4, 3.8 Hz, 1H), 1.42 (dt, J = 19.8, 8.0 Hz, 1H), 1.05 (d, J = 12.7 Hz, 9H) ppm. ^{13}C NMR (126 MHz, chloroform-*d*, Figure S44) δ 146.95, 134.75, 126.98, 125.06, 121.66 (d, J = 4.5 Hz), 28.40 (d, J = 15.5 Hz), 22.64 (d, J = 41.6 Hz), 15.29 (d, J = 48.7 Hz) ppm. ^{31}P{^{1}H} NMR (162 MHz, chloroform-*d*, Figure S45) δ –141.86 (s) ppm.

S1.4.8 2-(1-(*tert*-butyl)phosphiran-2-yl)pyridine, 1h

The title compound was isolated as a borane adduct due to its instability on activated charcoal plug. A solution of ^{t}BuP**A** (266 mg, 1.0 mmol, 1.0 equiv), Fp_2 (7 mg, 0.02 mmol, 2 mol%) and 2-vinylpyridine (105 mg, 1.0 mmol, 1.0 equiv) in THF (15 mL) was heated at 80 °C with stirring in a sealed Schlenk tube covered by aluminum foil for 16 h. After cooling to room temperature, volatile materials were removed under reduced pressure. The residue was dissolved in Et_2O (40 mL) and triphenylborane (242 mg, 1.0 mmol, 1.0 equiv) was added. The solution was stirred for 30 min and passed through an activated

charcoal plug (ca. 5 cm thick) in a frit (15 mL, medium porosity). Additional Et_2O (2×50 mL) was used to wash the charcoal plug, and volatile materials were removed from the combined filtrate under reduced pressure. The crude product was recrystallized in minimal Et_2O (ca. 4 mL) at –35 °C, collected by decanting, rinsing with minimal cold Et_2O and drying under vacuum to yield **1h**-BPh_3 adduct as a white solid (292 mg, 0.67 mmol, 67%). Anal. Calcd for $C_{29}H_{31}BNP$: C, 80.01; H, 7.18; N, 3.22. Found: C, 80.02; H, 7.33; N, 3.47. ^{1}H NMR (500 MHz, chloroform-*d*, Figure S46) δ 8.40 (d, J = 5.2 Hz, 1H), 7.73 (t, J = 7.4 Hz, 1H), 7.39 (d, J = 7.1 Hz, 6H), 7.35 – 7.28 (m, 9H), 7.14 (t, J = 6.6 Hz, 1H), 6.91 (d, J = 8.0 Hz, 1H), 2.85 (ddd, J = 9.8, 7.5, 2.0 Hz, 1H), 1.07 – 0.88 (m, 2H), 0.68 (d, J = 12.5 Hz, 9H) ppm. ^{13}C NMR (126 MHz, chloroform-*d*, Figure S47) δ 164.68 (d, J = 8.5 Hz), 147.85, 138.51, 135.77, 127.20, 126.80 (br), 122.49 (d, J = 11.9 Hz), 120.64, 28.08 (d, J = 15.6 Hz), 27.20 (d, J = 32.8 Hz), 25.65 (d, J = 39.7 Hz), 16.70 (d, br, J = 60.0 Hz) ppm. ^{31}P{^{1}H} NMR (203 MHz, chloroform-*d*, Figure S48) δ –147.13 (br) ppm.

S1.4.9 (1-(*tert*-butyl)phosphiran-2-yl)triphenylphosphonium tetraphenylborate, 1i

Vinyltriphenylphosphonium tetraphenylborate was prepared by combining stoichiometric vinyltriphenylphosphonium bromide and sodium tetraphenylborate in water and collected by filtration and lyophilization. A solution of ^{t}BuP**A** (266 mg, 1.0 mmol, 1.0 equiv), Fp_2 (7 mg, 0.02 mmol, 2 mol%) and vinyltriphenylphosphonium tetraphenylborate (609 mg, 1.0 mmol, 1.0 equiv) in THF (50 mL) was heated at 80 °C with stirring in a sealed Schlenk tube covered by aluminum foil for 16 h. After cooling to room temperature, the solution was concentrated to 5 mL and triturated with 25 mL of hexanes. The formed precipitate was collected and washed with hexanes. The obtained solid was dissolved in THF (10 mL)

and passed through a Florisil plug (ca. 5 cm thick) in a frit (15 mL, medium porosity). Additional THF (2×15 mL) was used to wash the Florisil plug, and the crude product was recrystallized in 1:1 THF/hexanes (ca. 10 mL) at –35 °C to yield **1i** as a beige solid (446 mg, 0.64 mmol, 64%). ^{1}H NMR (500 MHz, chloroform-*d*, Figure S49) δ 7.71 (t, J = 7.3 Hz, 3H), 7.51 (td, J = 7.8, 3.5 Hz, 6H), 7.46 – 7.35 (m, 14H), 6.94 (t, J = 7.3 Hz, 8H), 6.81 (t, J = 7.2 Hz, 4H), 1.99 (ddd, J = 10.2, 7.9, 2.2 Hz, 1H), 1.78 (qd, J = 10.4, 4.1 Hz, 1H), 1.00 (d, J = 13.4 Hz, 9H), 0.93 – 0.86 (m, 1H) ppm. ^{13}C NMR (126 MHz, chloroform-*d*, Figure S50) δ 164.25 (dd, J = 98.7, 49.2 Hz), 136.32, 135.61 (d, J = 2.9 Hz), 133.45 (d, J = 9.6 Hz), 130.54 (d, J = 12.7 Hz), 125.49 (dd, J = 5.3, 2.5 Hz), 121.61, 118.00 (d, J = 89.7 Hz), 28.12 (d, J = 15.9 Hz), 27.33 (dd, J = 34.9, 4.1 Hz), 8.69 (dd, J = 47.7, 7.2 Hz), 7.35 (dd, J = 80.5, 50.1 Hz) ppm. ^{31}P{^{1}H} NMR (203 MHz, chloroform-*d*, Figure S51) δ 26.44 (d, J = 34.7 Hz), –171.02 (d, J = 35.1 Hz) ppm. No satisfactory elemental analysis data could be obtained due to residual solvents that cannot be removed in vacuo.

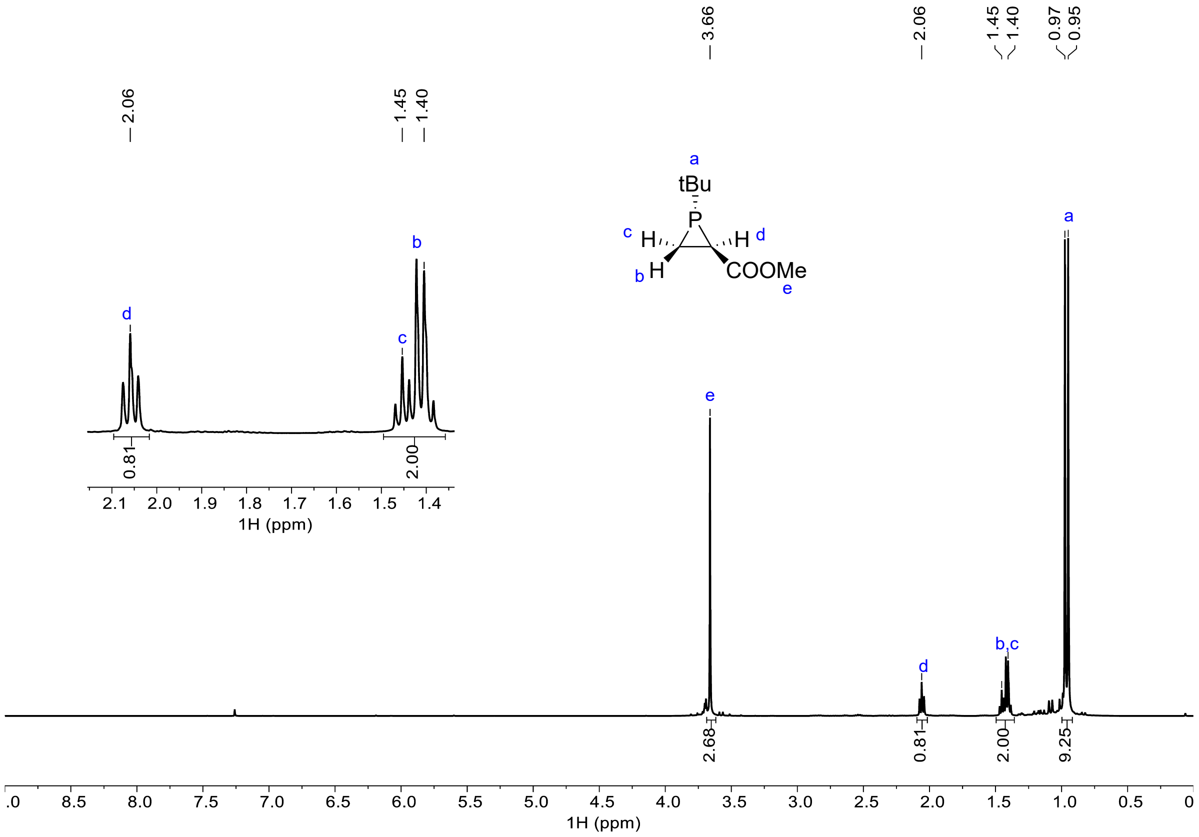

Figure S28: ^{1}H NMR spectrum of **1b** in $CDCl_3$ at 25 °C, recorded at 500 MHz.

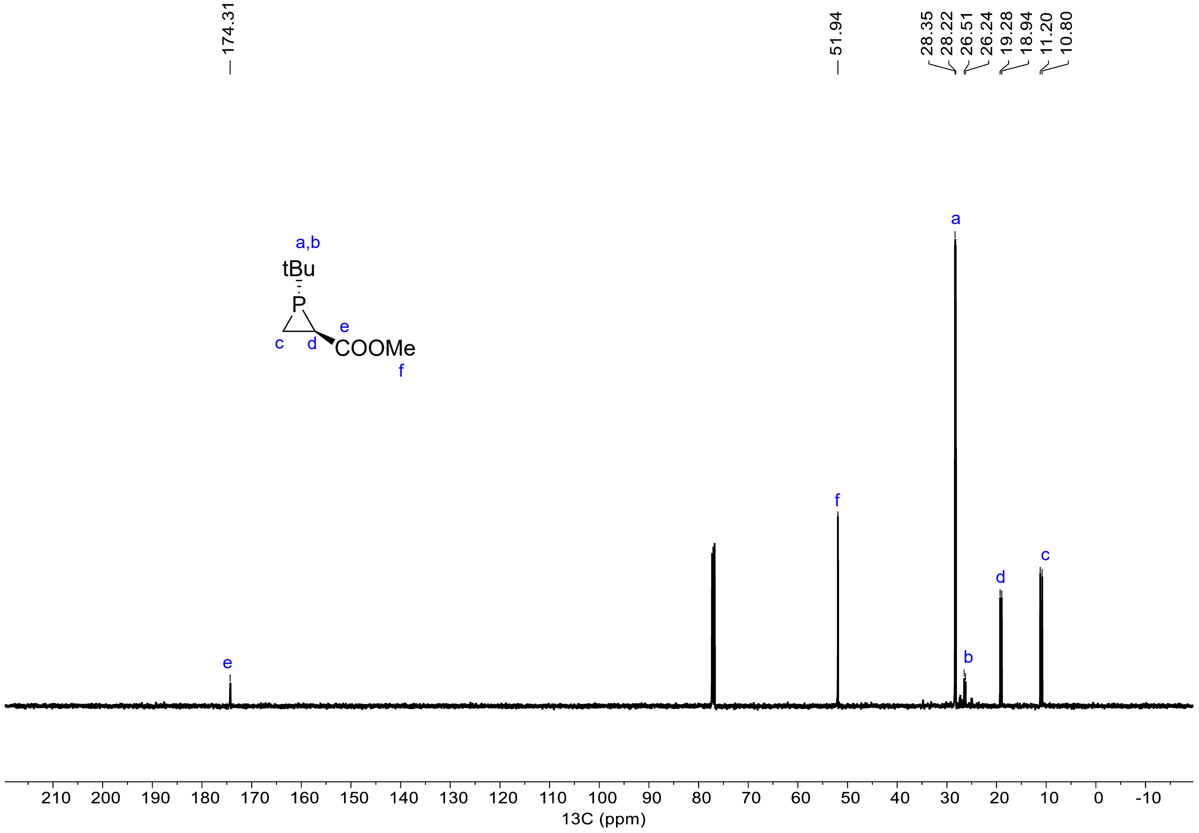

Figure S29: ^{13}C NMR spectrum of **1b** in $CDCl_3$ at 25 °C, recorded at 126 MHz.

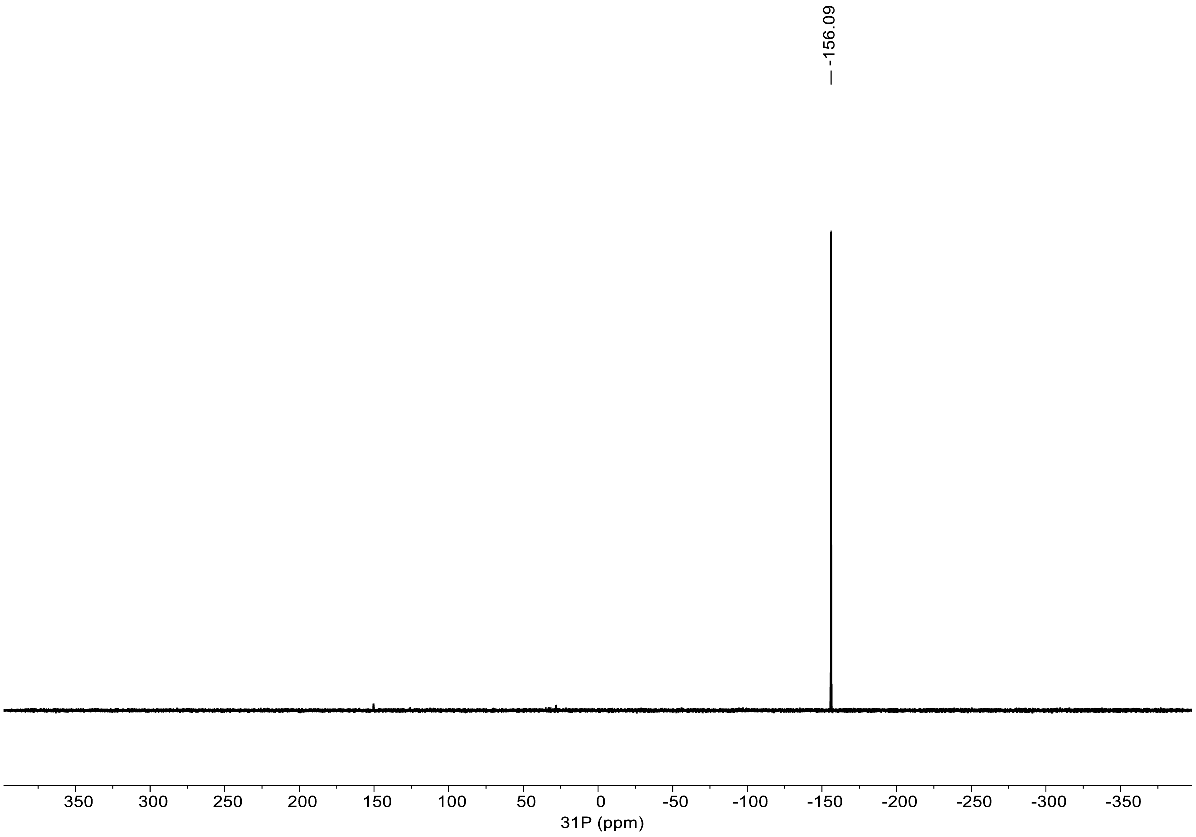

Figure S30: $^{31}P\{^1H\}$ NMR spectrum of **1b** in $CDCl_3$ at 25 °C, recorded at 203 MHz.

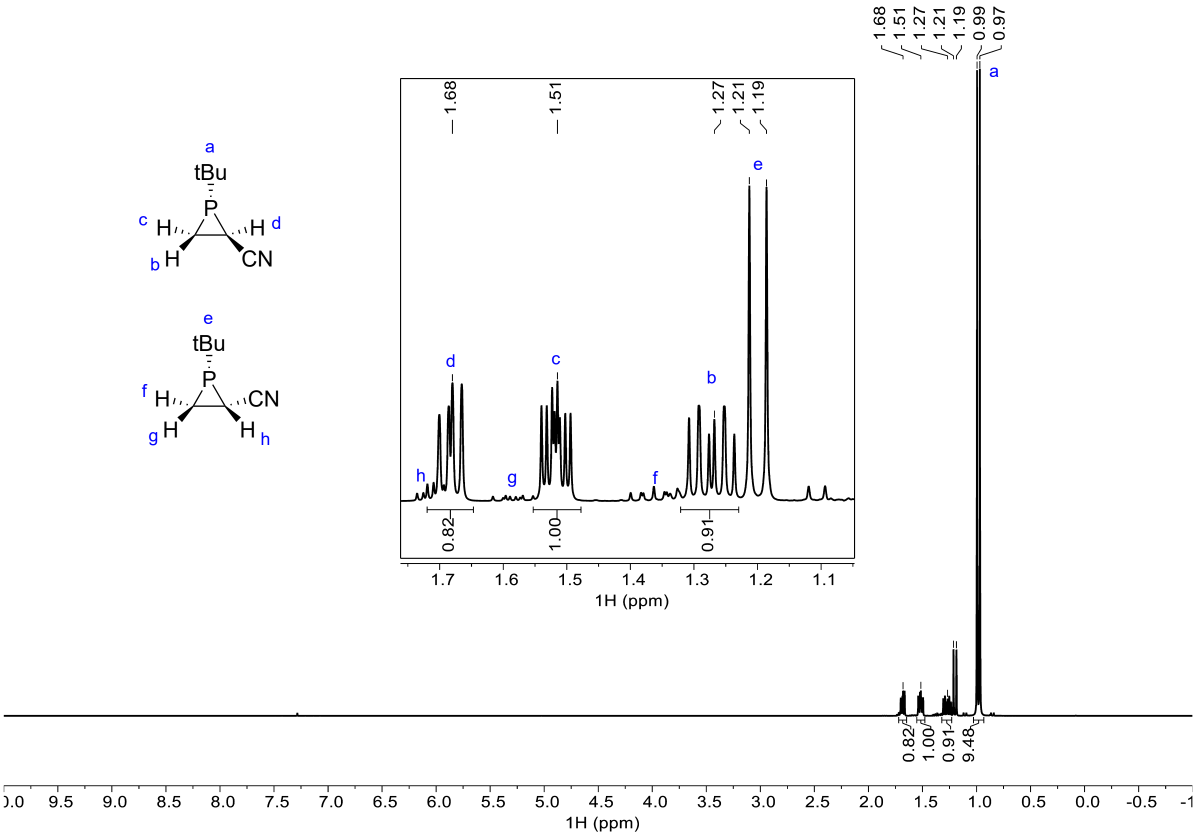

Figure S31: ^{1}H NMR spectrum of **1c** in $CDCl_3$ at 25 °C, recorded at 500 MHz.

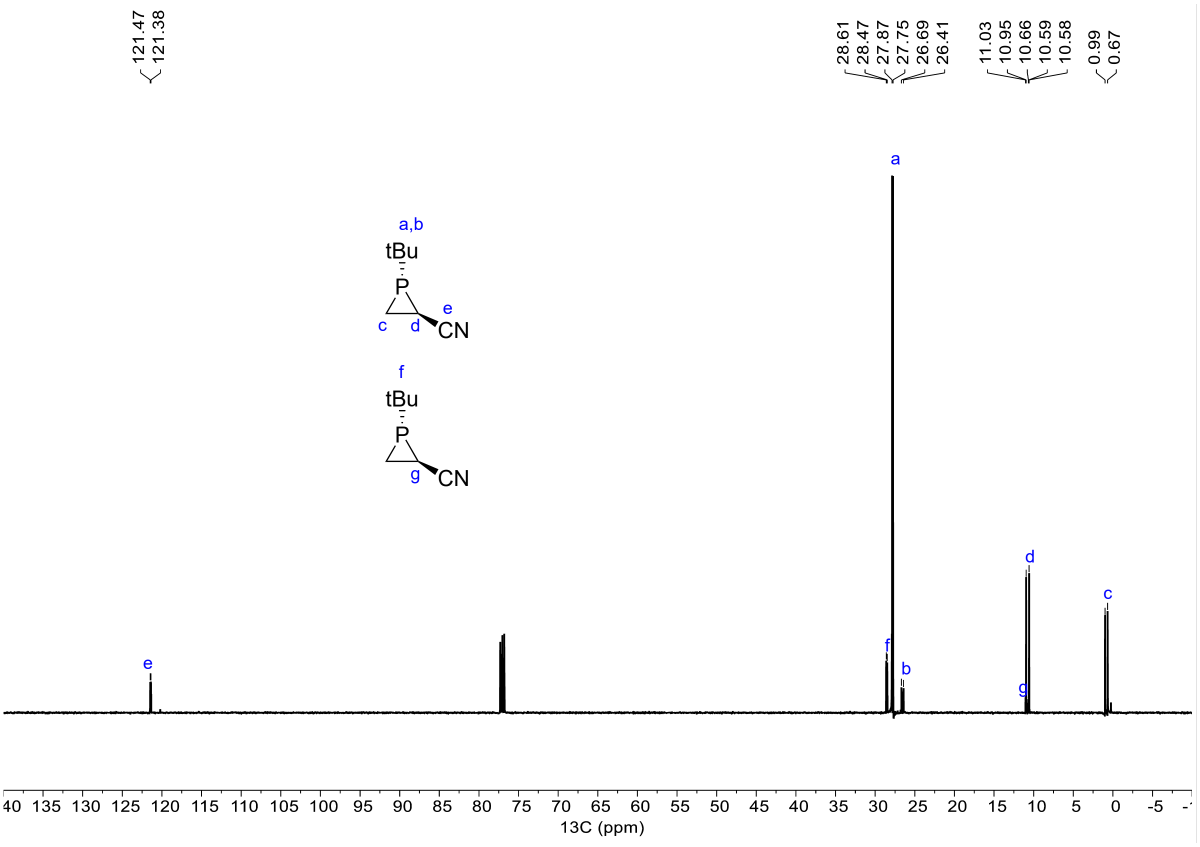

Figure S32: ^{13}C NMR spectrum of **1c** in $CDCl_3$ at 25 °C, recorded at 126 MHz.

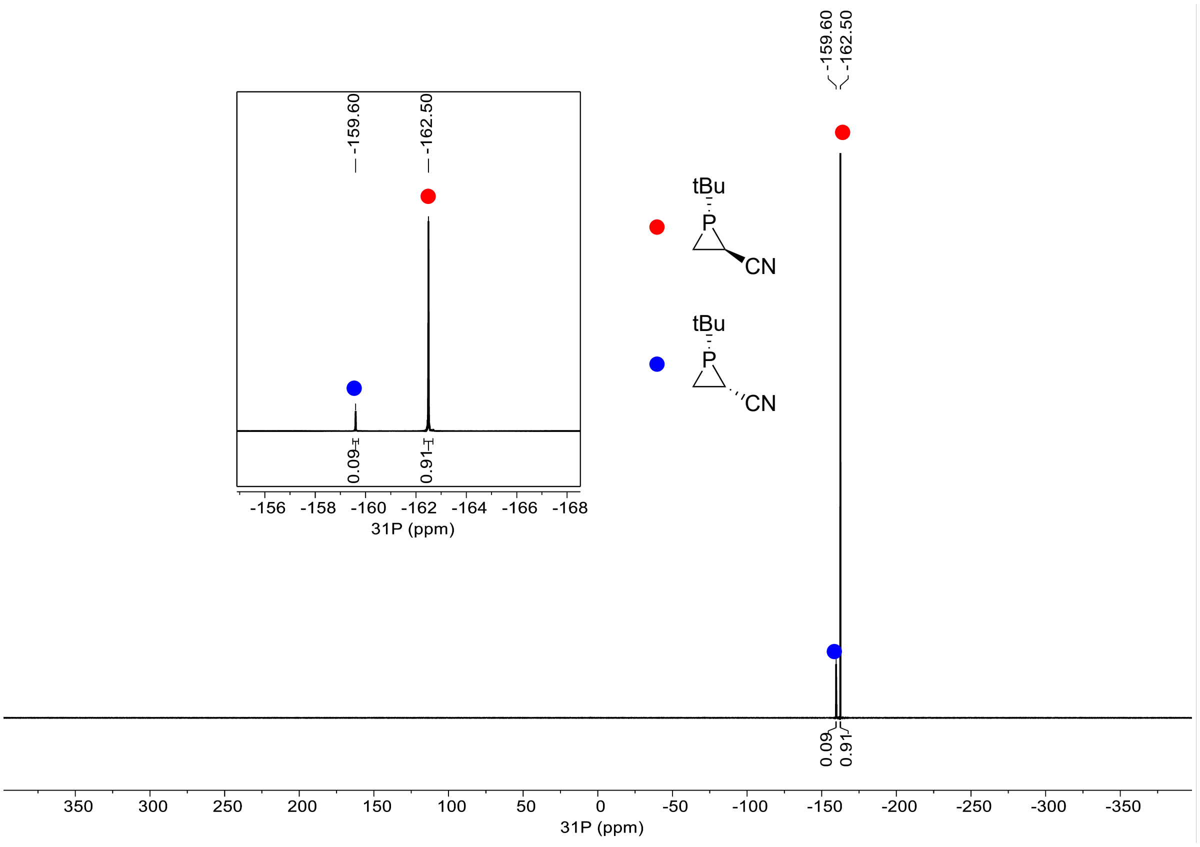

Figure S33: $^{31}P\{^{1}H\}$ NMR spectrum of **1c** in $CDCl_3$ at 25 °C, recorded at 203 MHz.

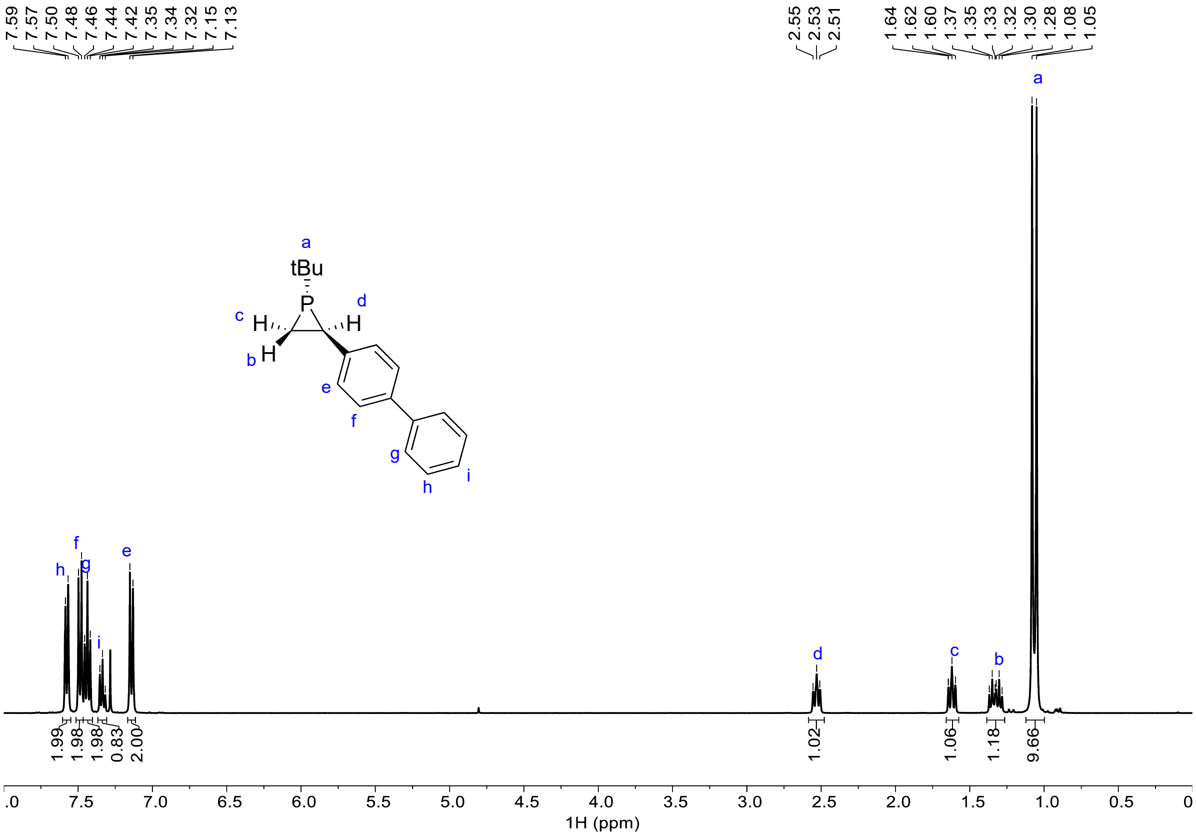

Figure S34: ^{1}H NMR spectrum of **1d** in $CDCl_3$ at 25 °C, recorded at 400 MHz.

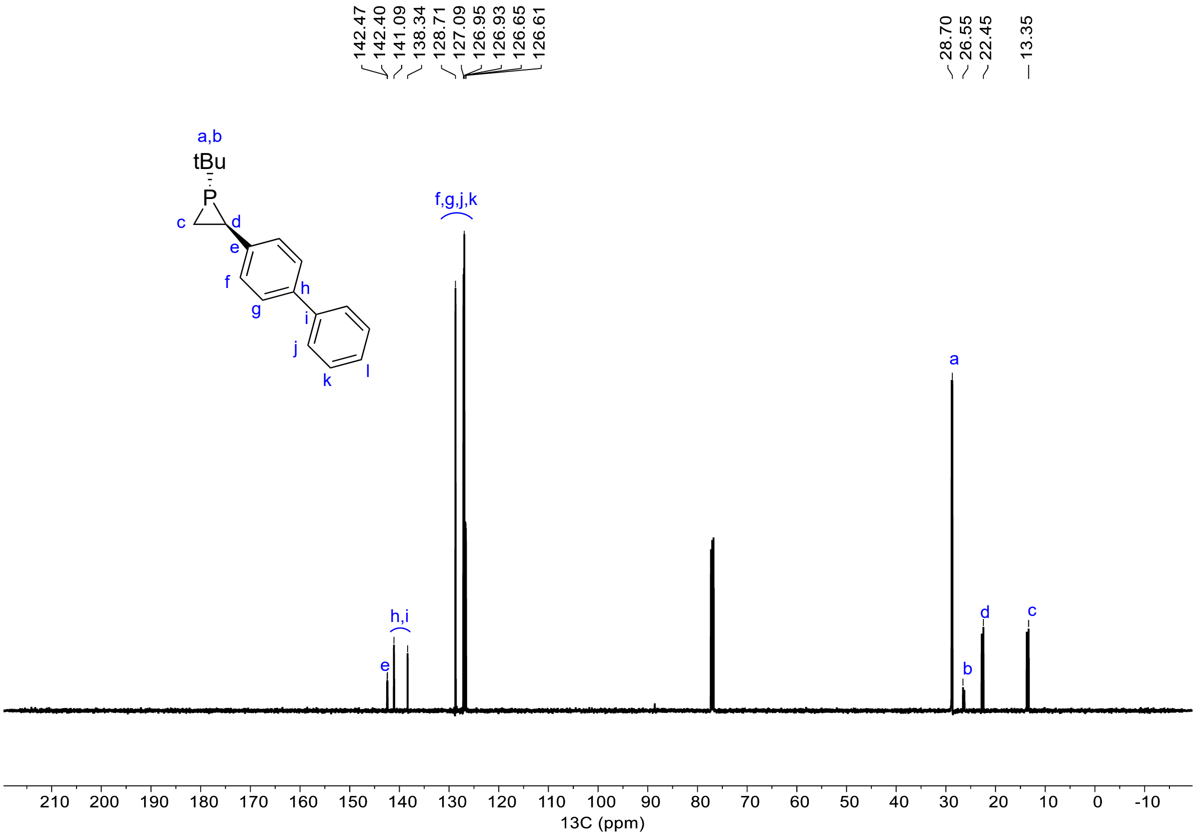

Figure S35: ^{13}C NMR spectrum of **1d** in $CDCl_3$ at 25 °C, recorded at 126 MHz.

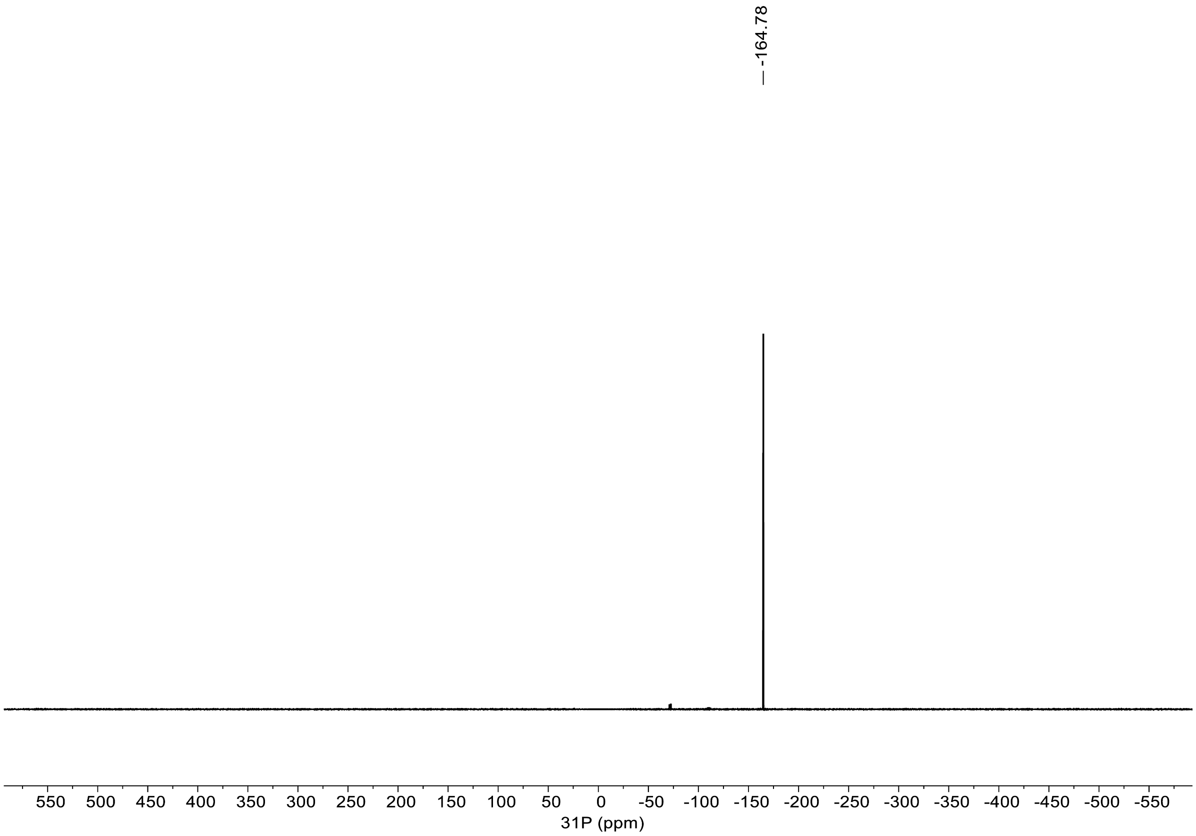

Figure S36: $^{31}P\{^{1}H\}$ NMR spectrum of **1d** in $CDCl_3$ at 25 °C, recorded at 162 MHz.

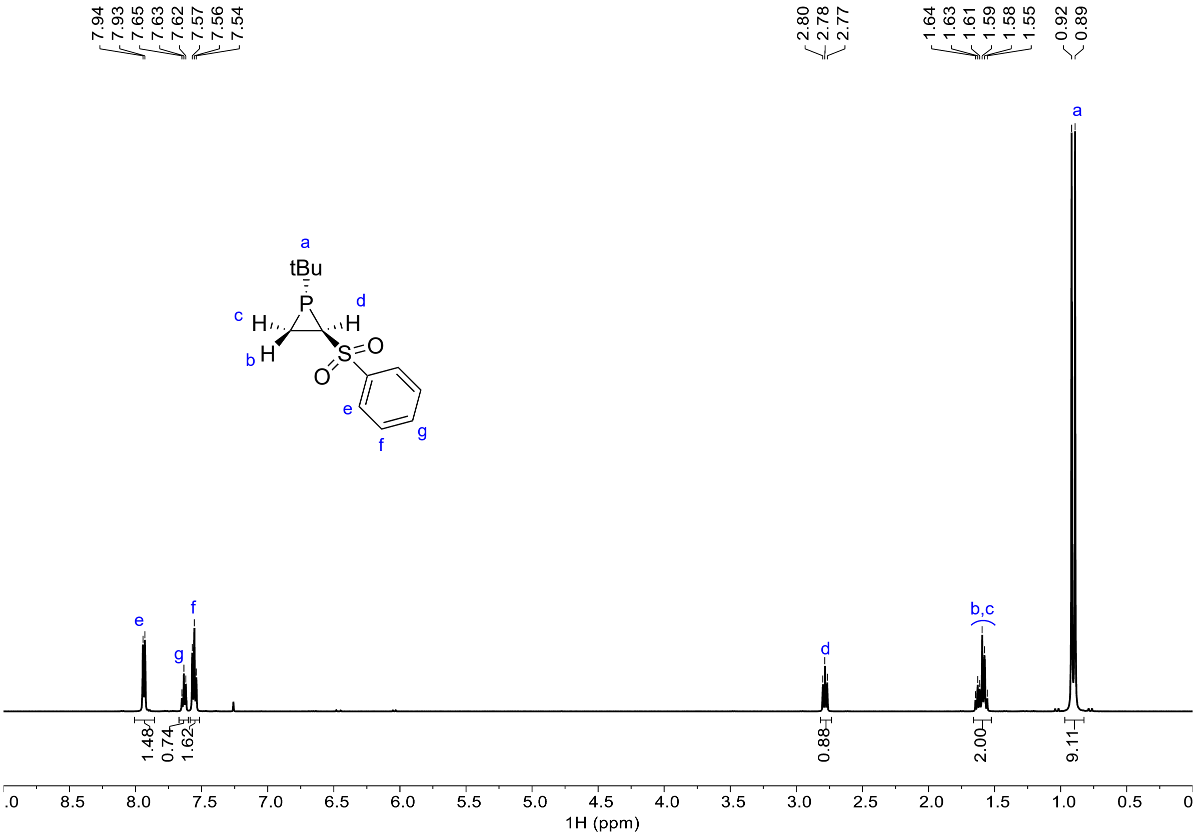

Figure S37: ^{1}H NMR spectrum of **1e** in $CDCl_3$ at 25 °C, recorded at 500 MHz.

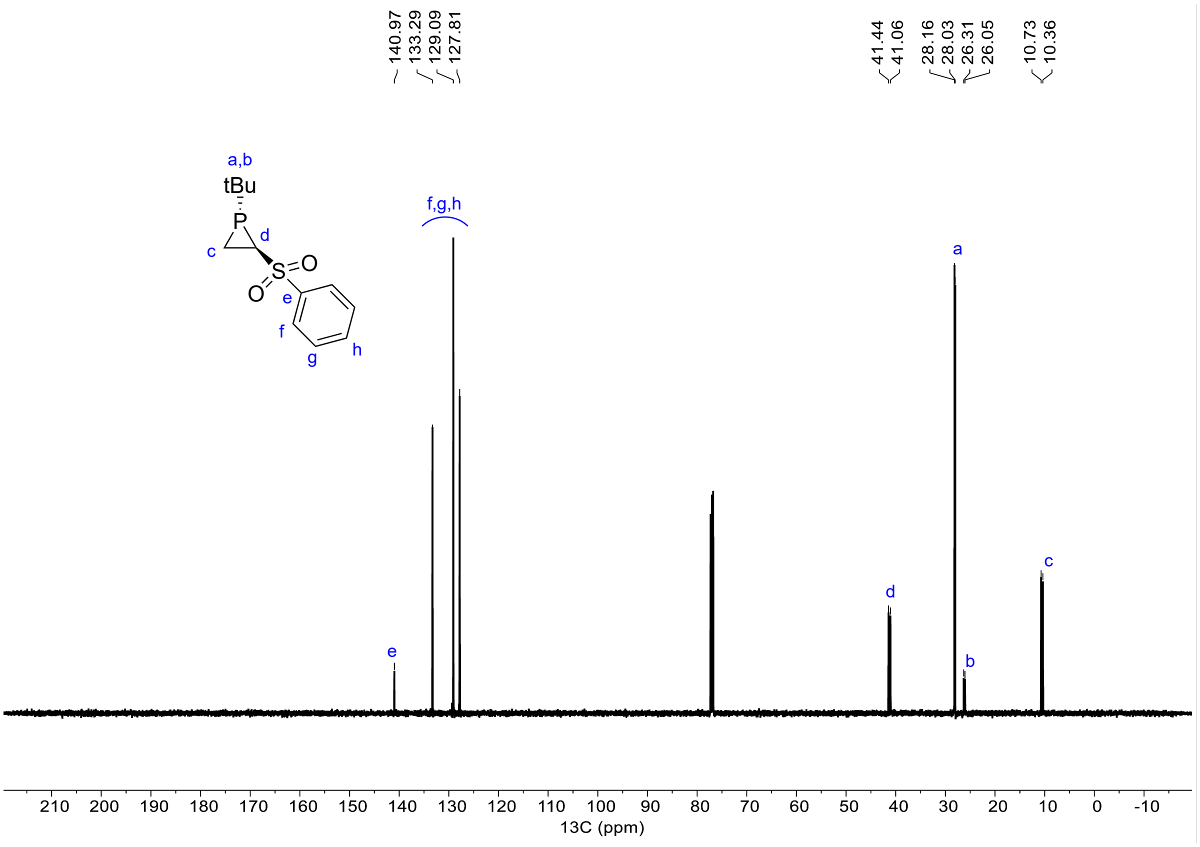

Figure S38: ^{13}C NMR spectrum of **1e** in $CDCl_3$ at 25 °C, recorded at 126 MHz.

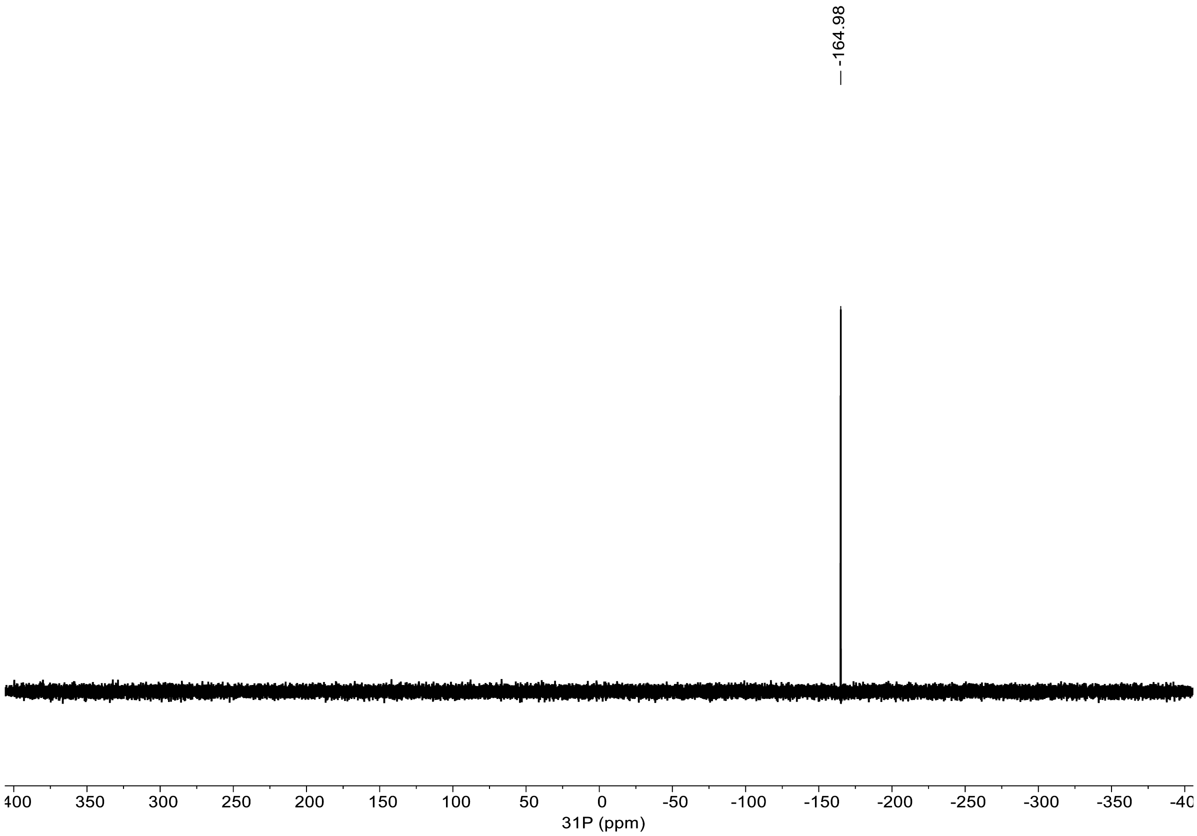

Figure S39: $^{31}P\{^{1}H\}$ NMR spectrum of **1e** in $CDCl_3$ at 25 °C, recorded at 162 MHz.

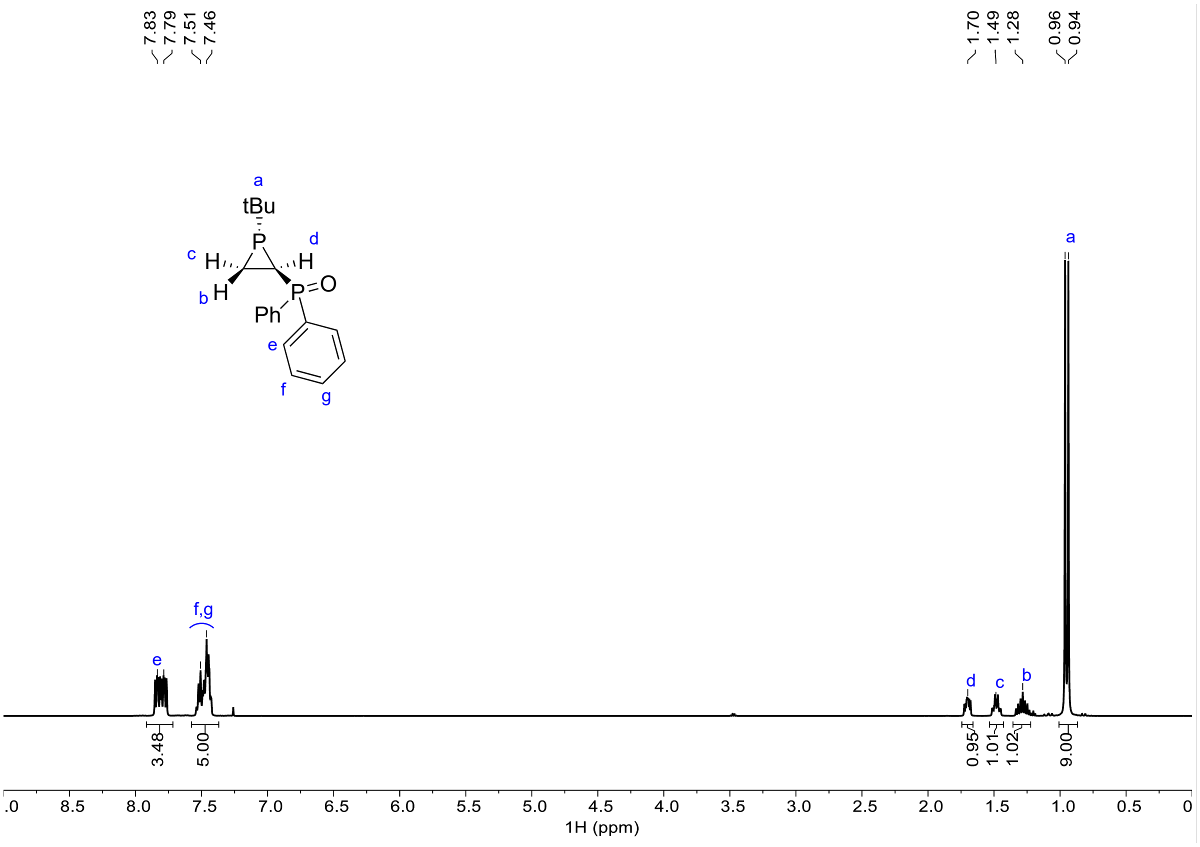

Figure S40: ^{1}H NMR spectrum of **1f** in $CDCl_3$ at 25 °C, recorded at 500 MHz.

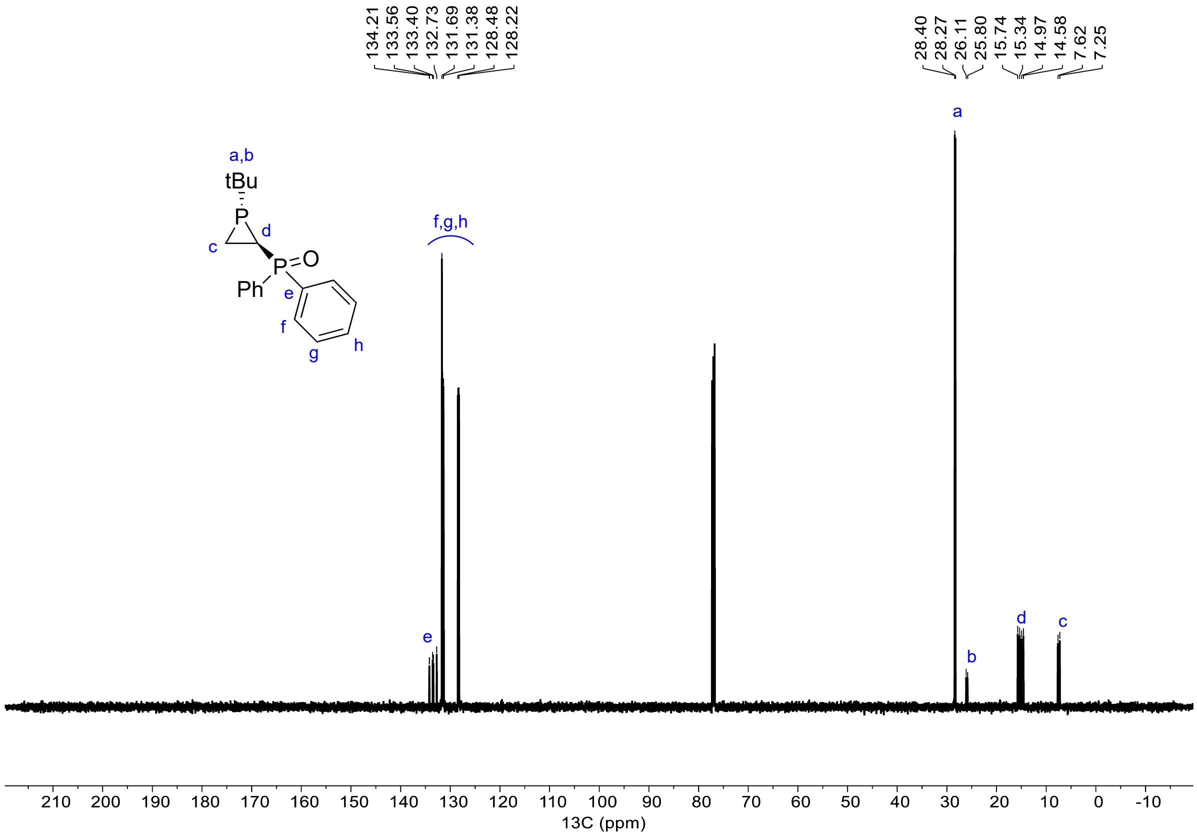

Figure S41: ^{13}C NMR spectrum of **1f** in $CDCl_3$ at 25 °C, recorded at 126 MHz.

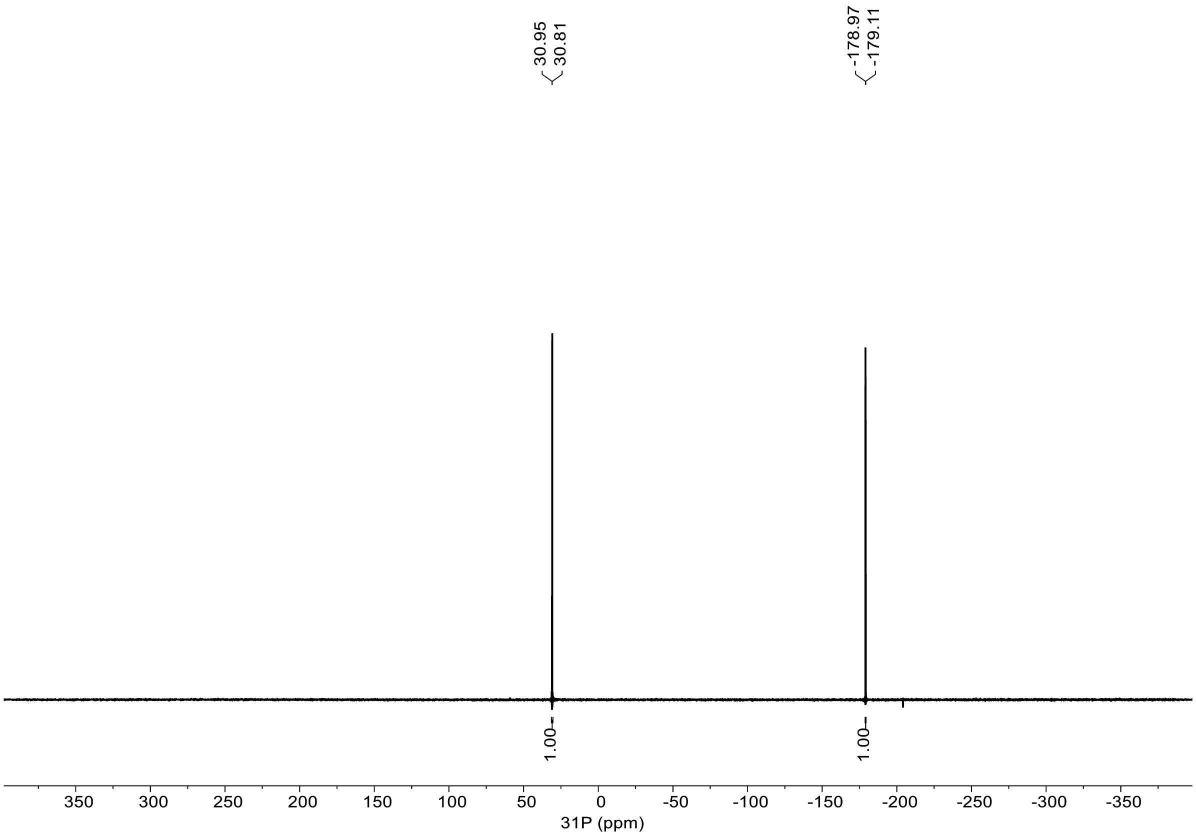

Figure S42: $^{31}P\{^{1}H\}$ NMR spectrum of **1f** in $CDCl_3$ at 25 °C, recorded at 203 MHz.

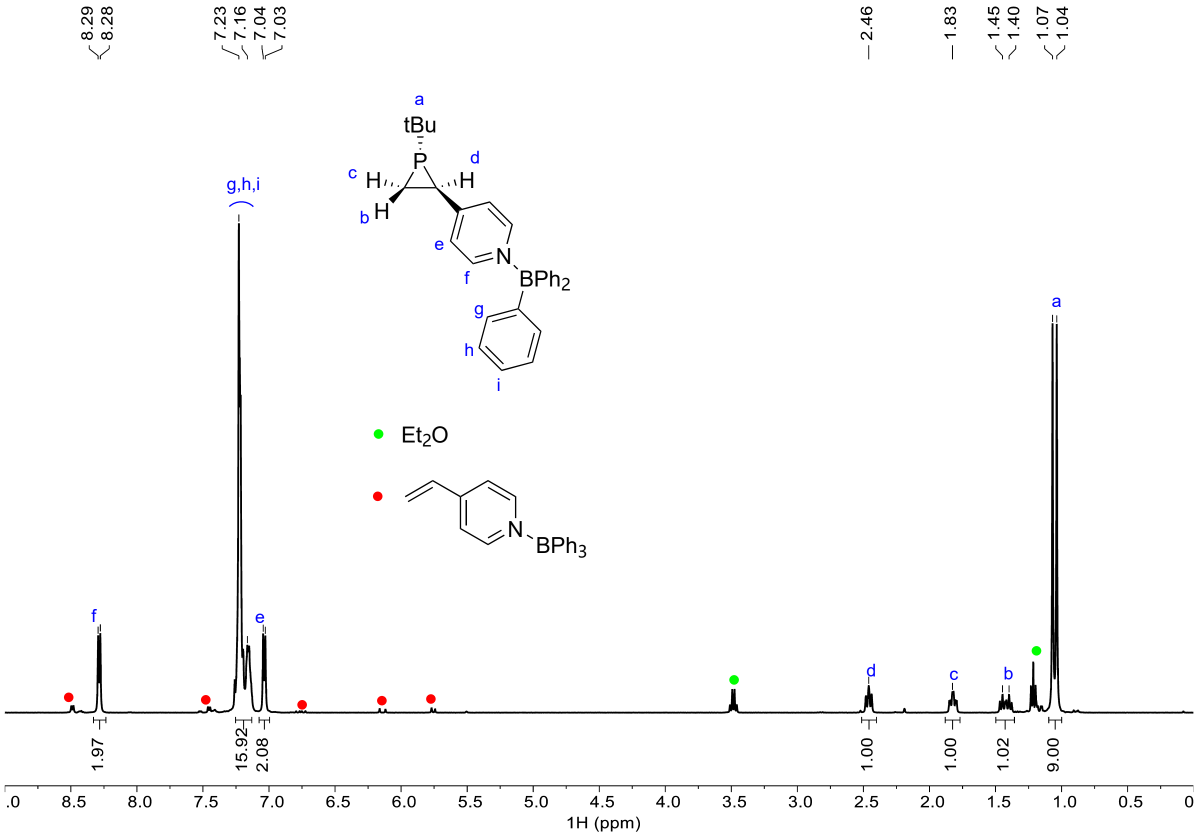

Figure S43: ^{1}H NMR spectrum of **1g** in $CDCl_3$ at 25 °C, recorded at 400 MHz.

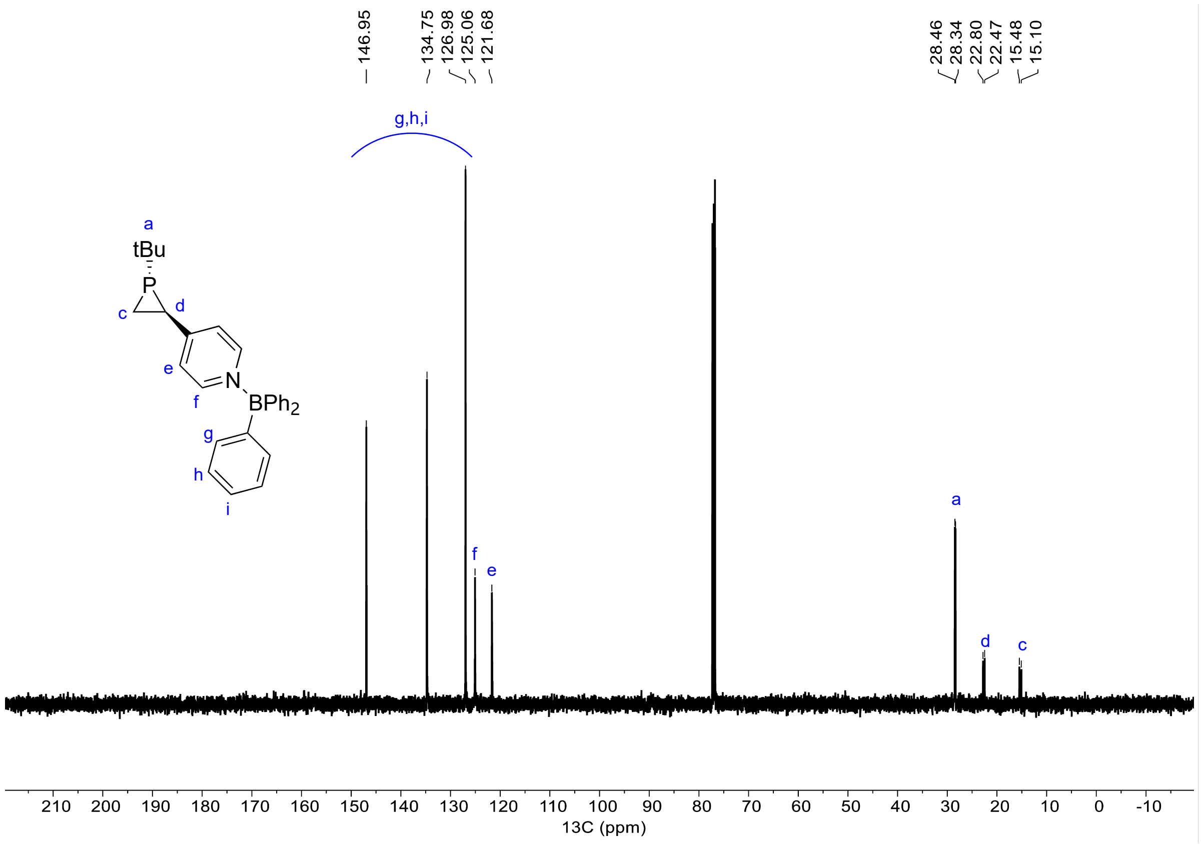

Figure S44: ^{13}C NMR spectrum of **1g** in $CDCl_3$ at 25 °C, recorded at 126 MHz.

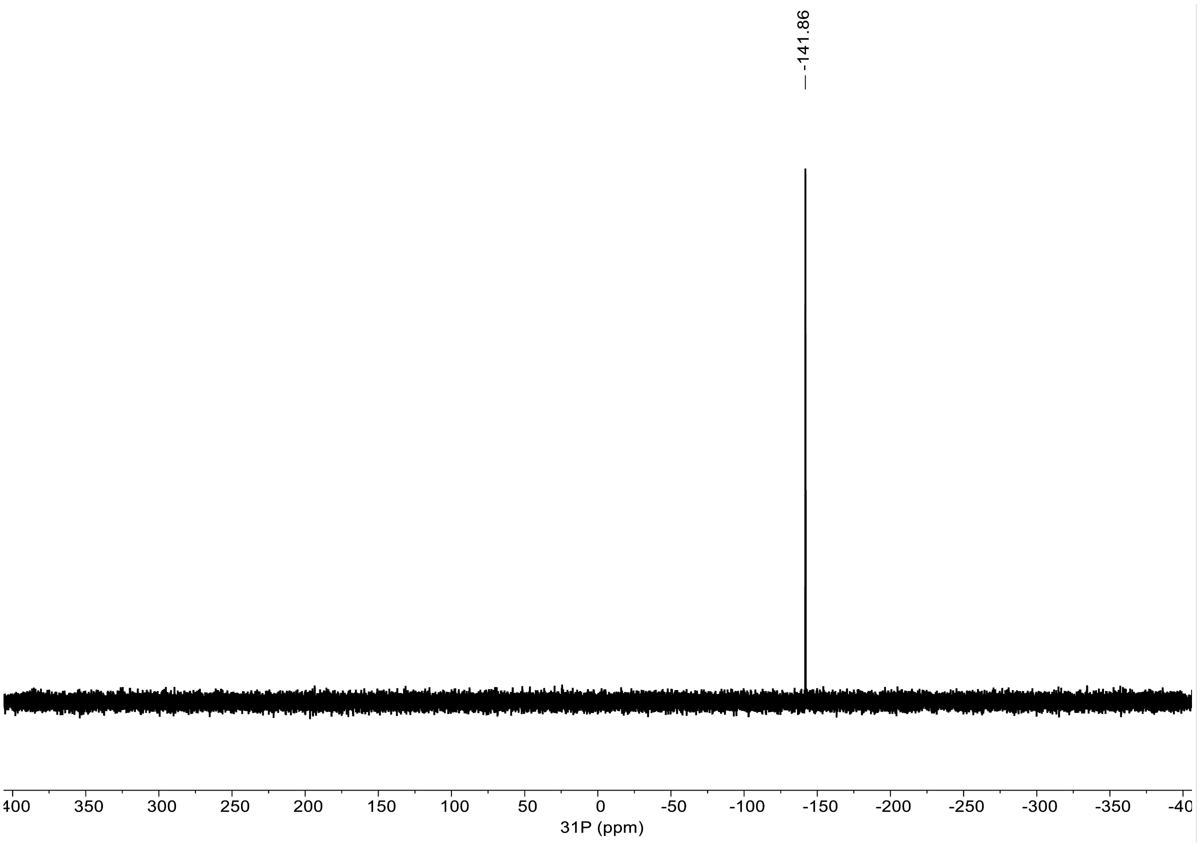

Figure S45: $^{31}P\{^{1}H\}$ NMR spectrum of **1g** in $CDCl_3$ at 25 °C, recorded at 162 MHz.

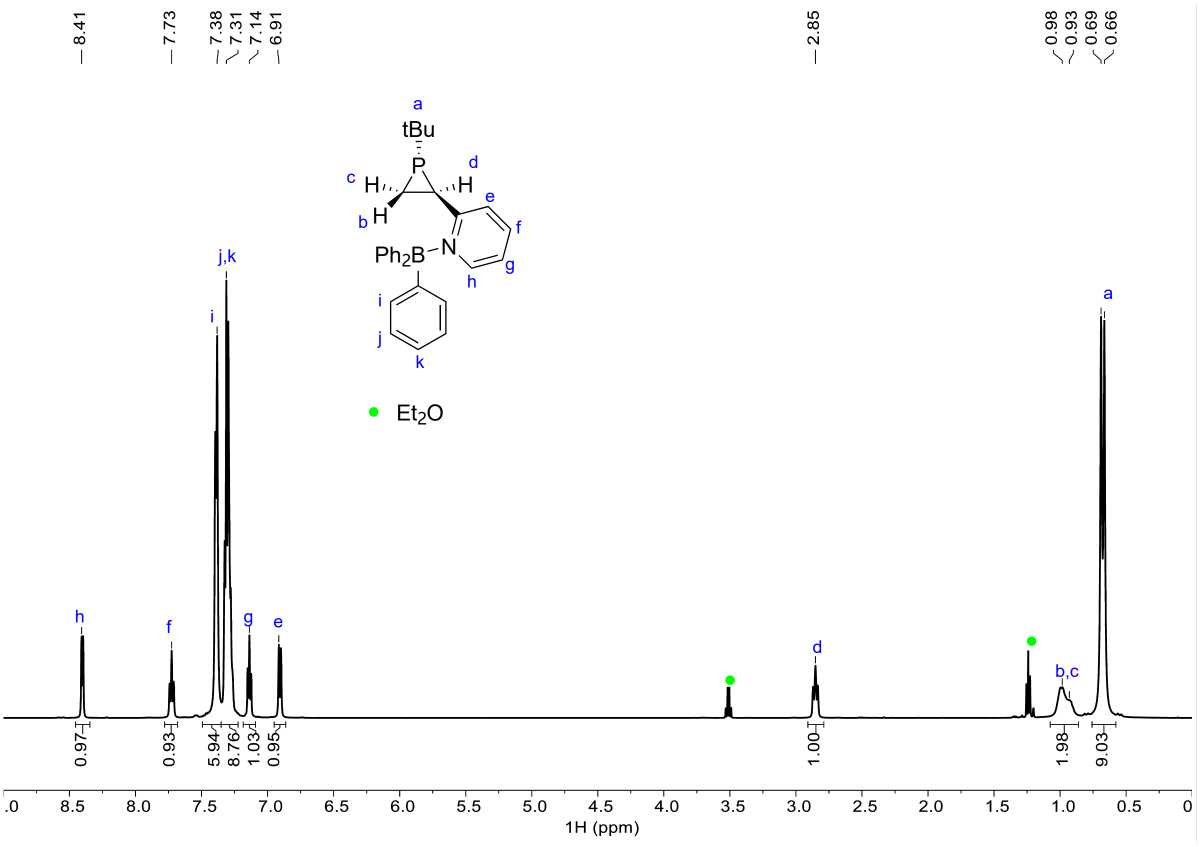

Figure S46: ^{1}H NMR spectrum of **1h** in $CDCl_3$ at 25 °C, recorded at 500 MHz.

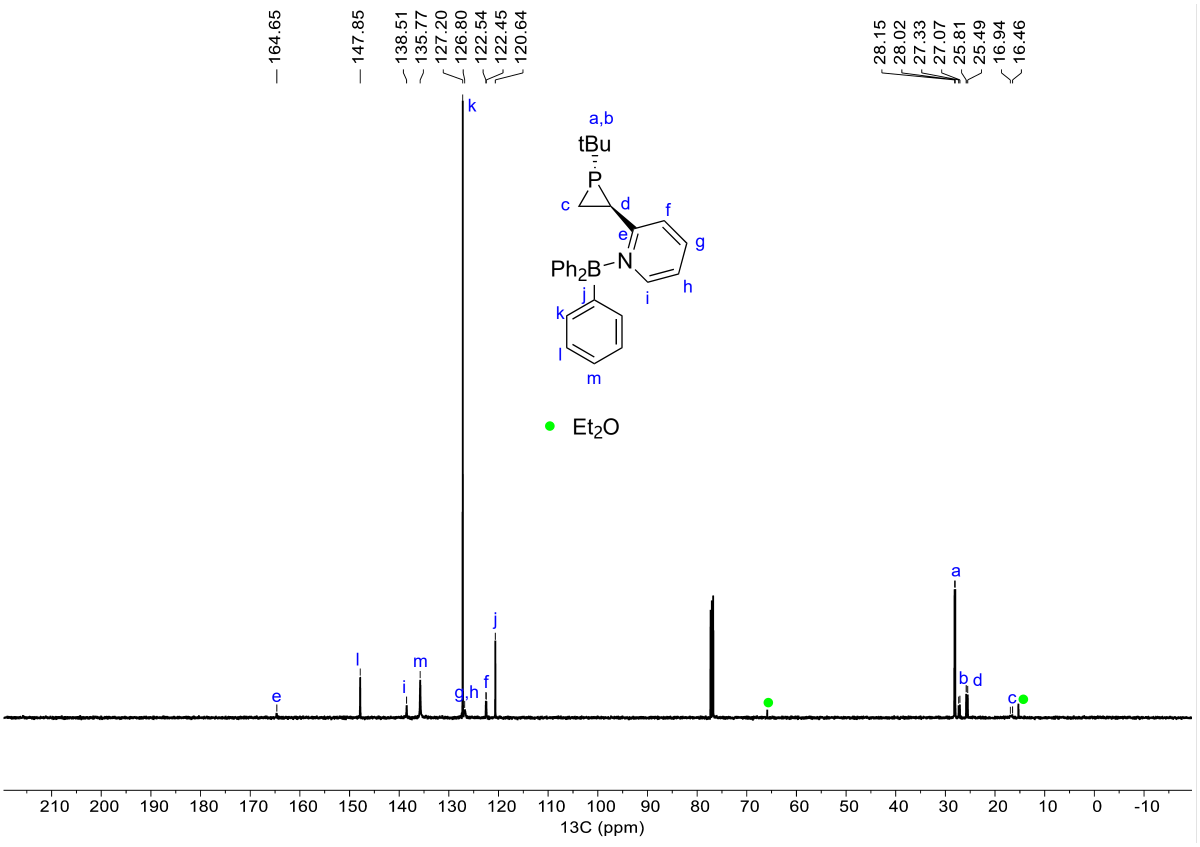

Figure S47: ^{13}C NMR spectrum of **1h** in $CDCl_3$ at 25 °C, recorded at 126 MHz.

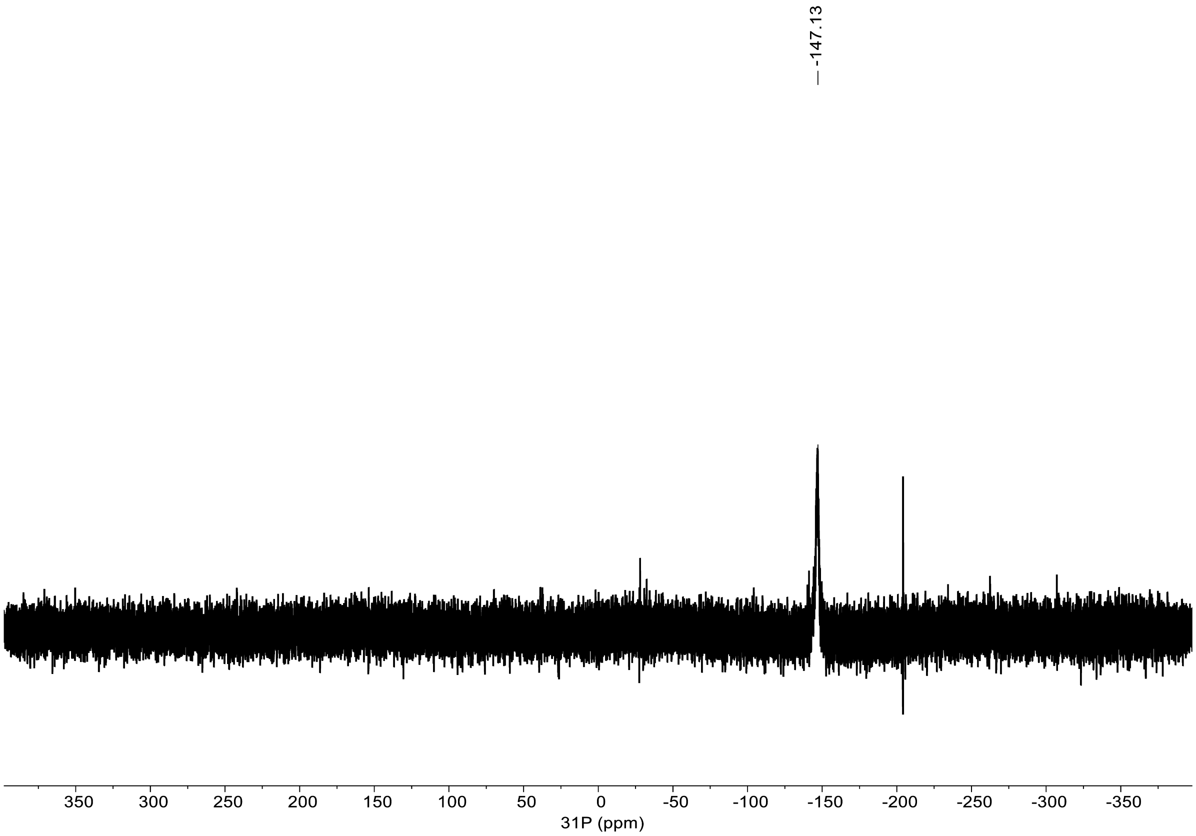

Figure S48: $^{31}P\{^{1}H\}$ NMR spectrum of **1h** in $CDCl_3$ at 25 °C, recorded at 203 MHz.

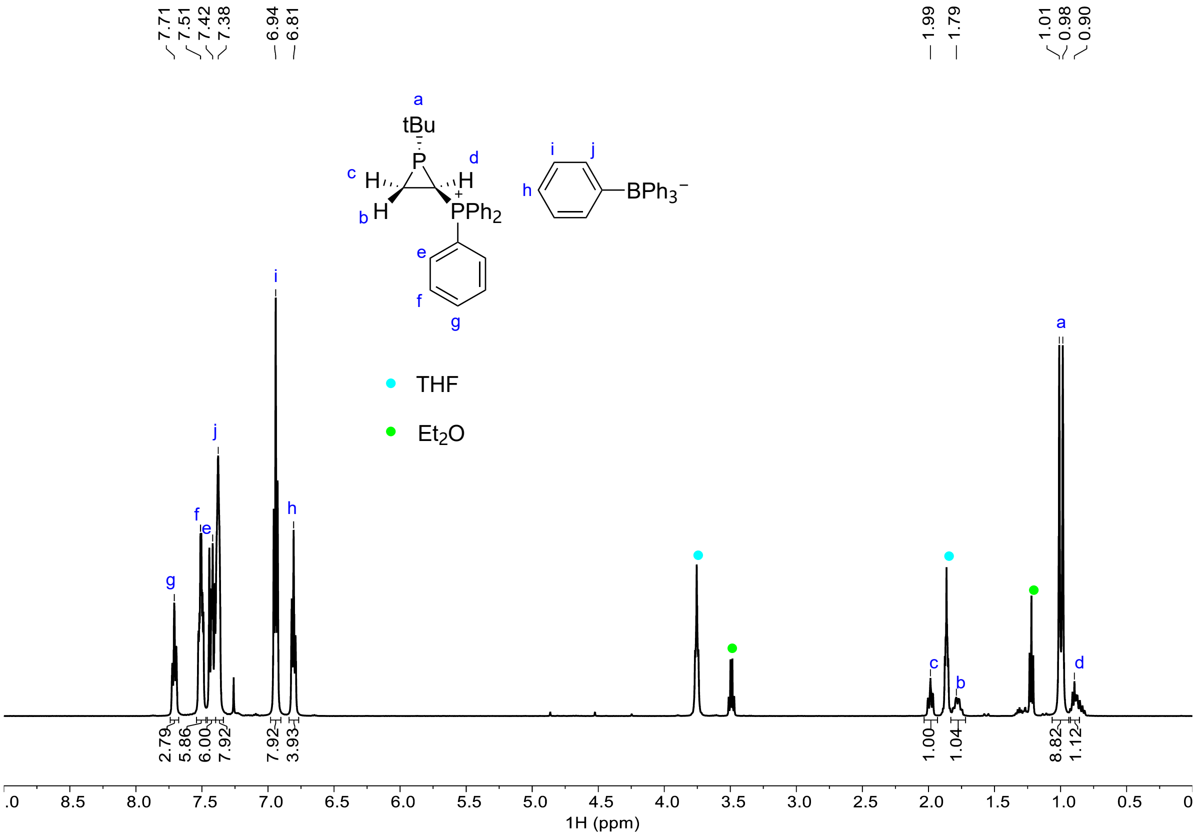

Figure S49: ^{1}H NMR spectrum of **1i** in $CDCl_3$ at 25 °C, recorded at 500 MHz.

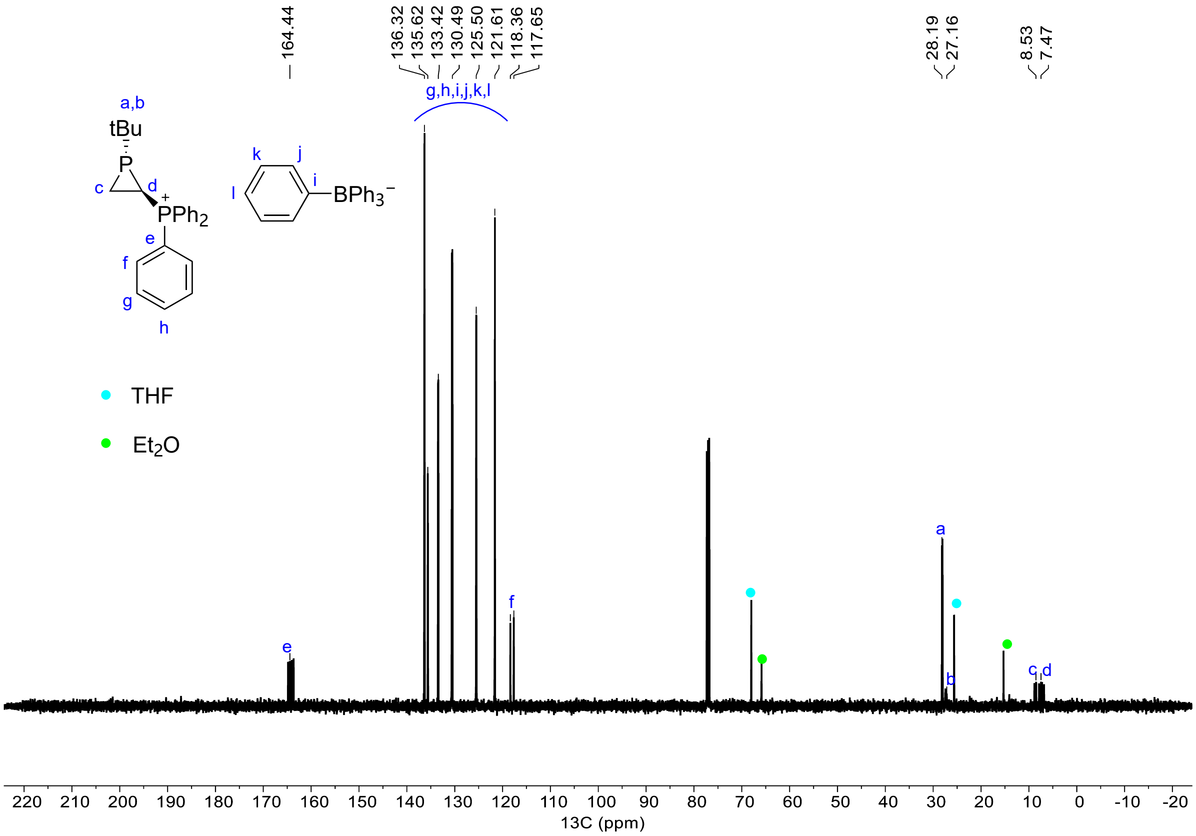

Figure S50: ^{13}C NMR spectrum of **1i** in $CDCl_3$ at 25 °C, recorded at 126 MHz.

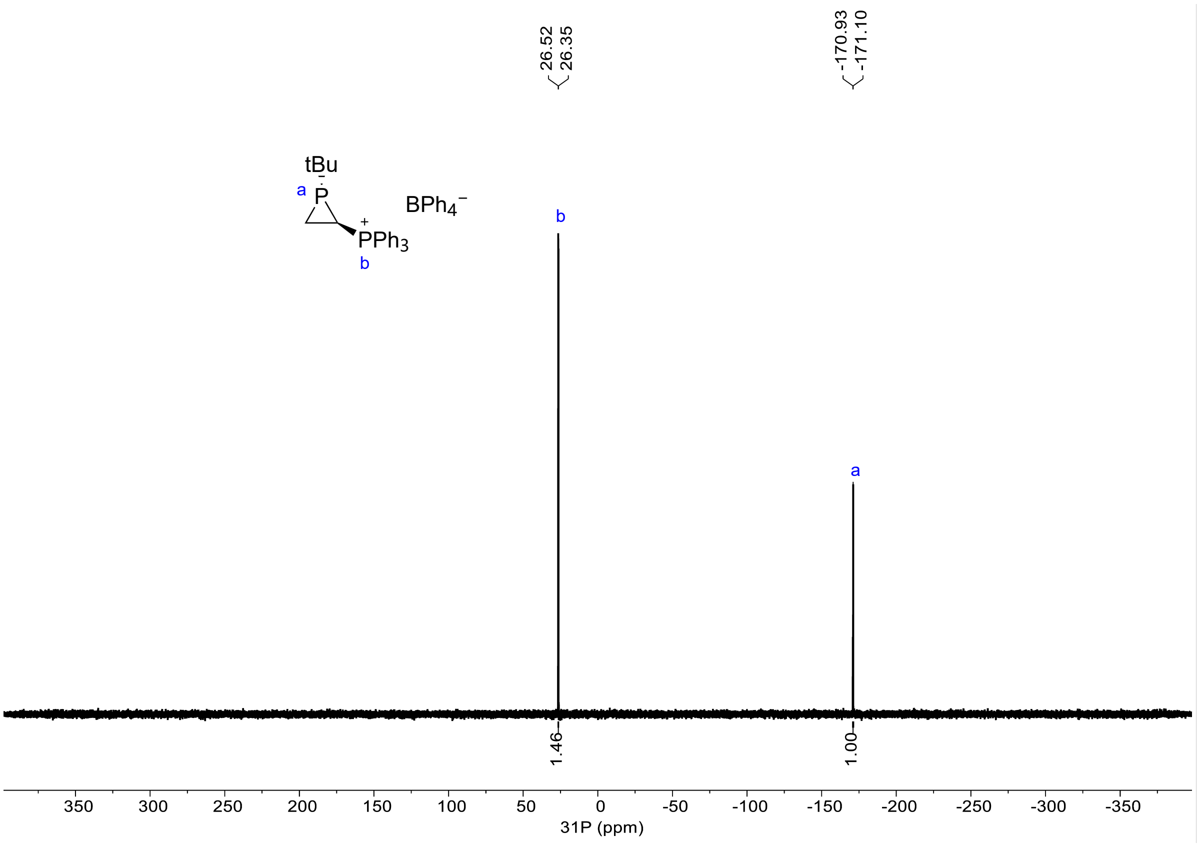

Figure S51: $^{31}P\{^{1}H\}$ NMR spectrum of **1i** in $CDCl_3$ at 25 °C, recorded at 203 MHz.

S2 X-ray crystallography

S2.1 General considerations

Low-temperature (100 K) diffraction data were collected on a Bruker-AXS X8 Kappa Duo diffractometer with $I\mu S$ micro-sources, coupled to a Photon 3 CPAD detector using Mo K_α radiation ($\lambda = 0.71073$ Å) performing ϕ-and ω-scans. The structures were solved by dual-space methods using SHELXT[S12] and refined against F^2 on all data by full-matrix least squares with SHELXL-2017[S12] following established refinement strategies.[S13] All non-hydrogen atoms were refined anisotropically. All hydrogen atoms were included into the model at geometrically calculated positions and refined using a riding model. The isotropic displacement parameters of all hydrogen atoms were fixed to 1.2 times the U-value of the atoms they are linked to (1.5 times for methyl groups). Details of the data quality and a summary of the residual values of the refinement are listed in Table S2 to S4. Further details can be found in the form of .cif files available from the CCDC (CSD reference codes are given in tables).

S2.2 X-ray structure of 2-Cl

Red crystals of **2**-Cl were grown in pentane at −35 °C. The structure was was solved in the orthorhombic space group Pbca with one molecule of **2**-Cl in the asymmetric unit.

S2.3 X-ray structure of 2-F

Red crystals of **2**-F were grown in pentane at −35 °C. The structure was was solved in the orthorhombic space group Pbca with two molecules of **2**-F in the asymmetric unit. The P, F and ^{t}Bu moiety in one of the two molecules was disordered over two positions,

and the disorder ratio was refined freely and converged at 0.856(0.001).

S2.4 X-ray structure of 3

Orange crystals of **3** were grown in Et_2O at −35 °C. The structure was was solved in the monoclinic space group $P2_1/c$ with one molecule of **3** in the asymmetric unit.

Table S2: X-ray crystallographic information for **2**-Cl.

CSD reference code	2190120
Empirical formula	C11 H14 Cl Fe O2 P
Formula weight	300.49
Temperature	100(2) K
Wavelength	0.71073 Å
Crystal system	Orthorhombic
Space group	Pbca
Unit cell dimensions	a = 12.2903(3) Å α = 90°<br>b = 13.3012(3) Å β = 90°<br>c = 15.6001(3) Å γ = 90°
Volume	2550.24(10) Å^3
Z	8
Density (calculated)	1.565 g/cm^3
Absorption coefficient	1.499 mm^{-1}
$F(000)$	1232
Crystal size	0.250 × 0.180 × 0.150 mm^3
Theta ranges for data collection	2.607 to 32.629°
Index ranges	−18≤h≤18, −20≤k≤20, −23≤l≤23
Reflections collected	103880
Independent reflections	4667 [R_{int} = 0.0372]
Completeness to θ = 25.242°	100.0%
Refinement method	Full-matrix least-squares on F^2
Data / restraints / parameters	4667 / 0 / 148
Goodness-of-fit on F^2	1.045
Final R indices [$I>2\sigma(I)$]	R_1 = 0.0205, wR_2 = 0.0487
R indices (all data)	R_1 = 0.0257, wR_2 = 0.0513
Extinction coefficient	n/a
Largest diff. peak and hole	0.418 and −0.455 $e{\cdot}$A^{-3}

Table S3: X-ray crystallographic information for **2**-F.

CSD reference code	2190121
Empirical formula	C11 H14 F Fe O2 P
Formula weight	284.04
Temperature	100(2) K
Wavelength	0.71073 Å
Crystal system	Orthorhombic
Space group	Pbca
Unit cell dimensions	a = 21.4541(5) Å $\alpha = 90°$<br>b = 9.6814(2) Å $\beta = 90°$<br>c = 23.5240(5) Å $\gamma = 90°$
Volume	4886.07(18) Å^3
Z	16
Density (calculated)	1.545 g/cm^3
Absorption coefficient	1.360 mm^{-1}
$F(000)$	2336
Crystal size	0.220 × 0.120 × 0.115 mm^3
Theta ranges for data collection	1.731 to 32.629°
Index ranges	$-32\leq h\leq 32$, $-14\leq k\leq 14$, $-35\leq l\leq 35$
Reflections collected	200569
Independent reflections	8943 [R_{int} = 0.0449]
Completeness to $\theta = 25.242°$	100.0%
Refinement method	Full-matrix least-squares on F^2
Data / restraints / parameters	8943 / 0 / 343
Goodness-of-fit on F^2	1.039
Final R indices [$I>2\sigma(I)$]	R_1 = 0.0323, wR_2 = 0.0810
R indices (all data)	R_1 = 0.0427, wR_2 = 0.0877
Extinction coefficient	n/a
Largest diff. peak and hole	1.660 and −0.787 $e\cdot$A^{-3}

Table S4: X-ray crystallographic information for **3**.

CSD reference code	2190122
Empirical formula	C15 H20 Cl Fe O4 P
Formula weight	386.58
Temperature	100(2) K
Wavelength	0.71073 Å
Crystal system	Monoclinic
Space group	$P2_1/c$
Unit cell dimensions	a = 8.0716(2) Å α = 90°<br>b = 22.6406(5) Å β = 90°<br>c = 9.6109(2) Å γ = 90°
Volume	1671.01(7) $Å^3$
Z	4
Density (calculated)	1.537 g/cm^3
Absorption coefficient	1.171 mm^{-1}
$F(000)$	800
Crystal size	0.380 × 0.280 × 0.150 mm^3
Theta ranges for data collection	1.799 to 33.212°
Index ranges	$-12 \leq h \leq 12$, $-34 \leq k \leq 34$, $-14 \leq l \leq 14$
Reflections collected	83042
Independent reflections	6408 [R_{int} = 0.0287]
Completeness to θ = 25.242°	100.0%
Refinement method	Full-matrix least-squares on F^2
Data / restraints / parameters	6408 / 0 / 203
Goodness-of-fit on F^2	1.057
Final R indices [$I>2\sigma(I)$]	R_1 = 0.0207, wR_2 = 0.0547
R indices (all data)	R_1 = 0.0221, wR_2 = 0.0554
Extinction coefficient	n/a
Largest diff. peak and hole	0.456 and −0.481 $e \cdot A^{-3}$

S3 Computational Details

All DFT calculations are performed with the TURBOMOLE 7.4 suite of programs.[S14] The structures are fully optimized at the TPSS-D3/def2-TZVP + COSMO(THF) level, which combines the TPSS meta-GGA density functional[S15] with the BJ-damped DFT-D3 dispersion correction[S16,S17] and the def2-TZVP basis set,[S18] using the Conductor-like Screening Model (COSMO)[S19] for THF solvent (dielectric constant e = 7.58 and diameter Rsolv = 3.18 Å). The well-established density-fitting RI-J approach[S20] is used, which speeds up semi-local DFT functional calculations by a factor of 5-20 at practically no loss of accuracy. Chemically reasonable reaction paths are generated manually and tested in DFT calculations. Useful initial guesses of transition structures are obtained from interpolation between optimized intermediate structures as well as constrained optimizations with appropriate reaction coordinates. The optimized structures are characterized by frequency analysis (no imaginary frequency for true minima and only one imaginary frequency for transition states) to provide thermal free-energy corrections (at 298.15 K and 1 atm) according to the modified ideal gas-rigid rotor-harmonic oscillator model.[S21] The connection of the transition state with reactants and products is checked visually by careful examining the vibrational transition mode. More accurate solvation free energies in THF solution are computed with the COSMO-RS model[S22] (parameter file: BP_TZVP_16.ctd) using the COSMOtherm package[S23] based on the TPSS-D3 optimized structures, corrected by +1.89 kcal/mol to account for the 1 mol/L reference concentration in solution. To check the effects of the chosen density functional on the reaction energies and barriers, single-point calculations at both TPSS-D3 and hybrid-meta-GGA PW6B95-D3[S24] levels are performed using the large def2-QZVP basis set. Final reaction free energies (ΔG) are determined from the electronic single-point energies plus TPSS-D3

thermal corrections and COSMO-RS solvation free energies. For reactions with **2**-Cl as catalyst, both DFT functionals are in good mutual agreement of –0.4 ± 3.7 (average ± standard deviation) kcal/mol for relative reaction energies but the meta-GGA TPSS-D3 functional tends to predict 5.5 ± 3.0 kcal/mol too low reaction barriers (see S3), as also observed in our recent DFT studies.[S25–S35] For reactions with dinuclear Fp_2 complex as the catalyst, the meta-GGA TPSS-D3 functional seems to give 10 kcal/mol too high energies for each $Fp^{\cdot}$ radical species but still shows good mutual agreement for other close-shell reaction steps. In the discussion, more reliable PW6B95-D3 + COSMO-RS free energies (in kcal/mol, at 298.15 K and 1 mol/L concentration) are used unless specified otherwise. The applied DFT methods in combination with the large AO basis sets provide usually accurate electronic energies with typical absolute errors of 1-2 kcal/mol for chemical energies (including barriers), which has been tested thoroughly for the huge data base GMTKN55[S36] that is the common standard in the field of DFT benchmarking. To help NMR assignment, nuclear magnetic shielding constants are computed using the GIAO (Gauge Including Atomic Orbital) method[S37] at the TPSS/def2-QZVP level; final ^{31}P NMR chemical shifts are computed using the experimental ^{31}P NMR signal of trans-1-^{t}Bu-2-phenylphosphirane **1a** observed at –165.0 ppm in $CDCl_3$ solution as reference.

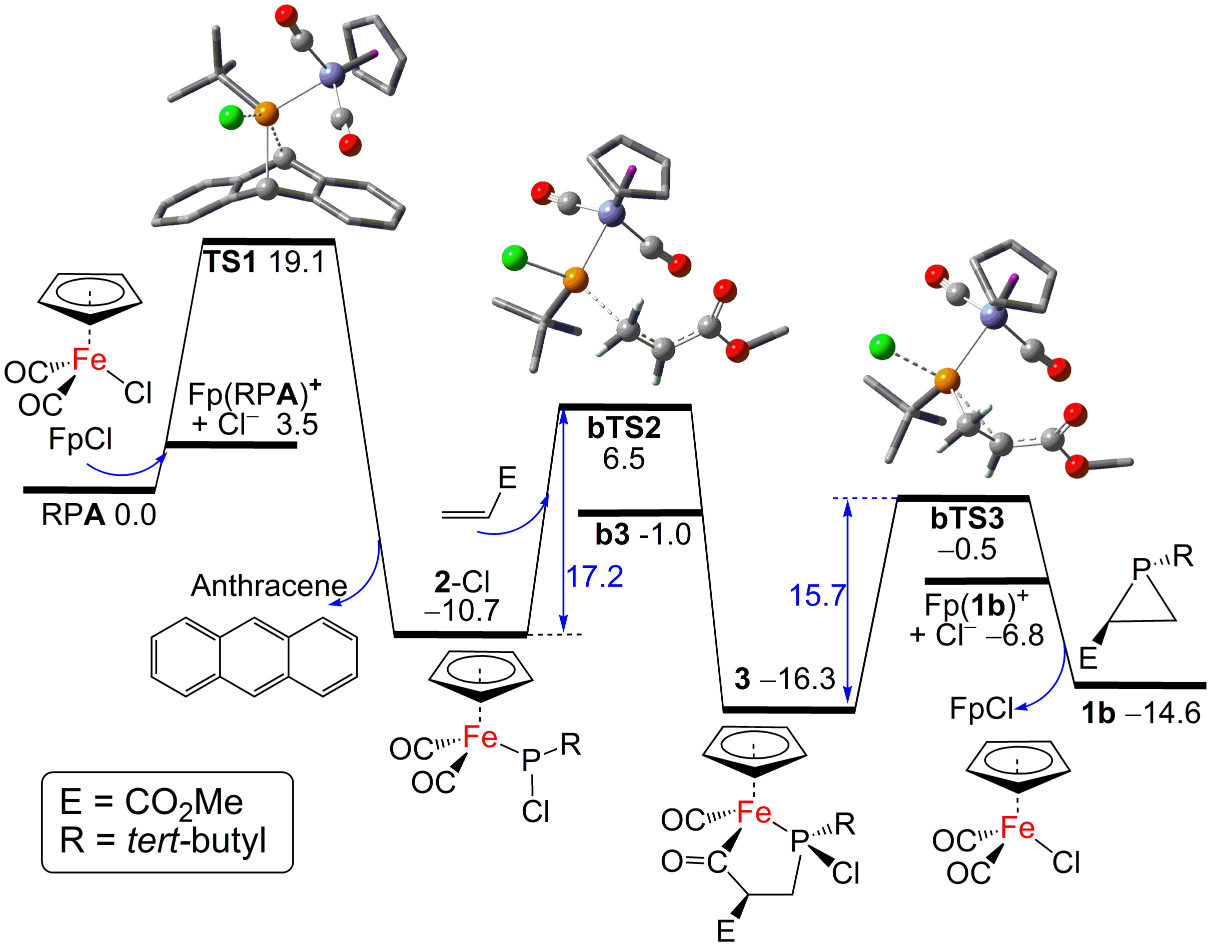

Figure S52: DFT computed free energy paths (in kcal/mol, at 298 K and 1 M in THF) for the **2**-Cl (or FpCl) catalyzed phosphinidene transfer from ^{t}BuP**A** to methyl acrylate at PW6B95-D3/def2-QZVP + COSMO-RS level using TPSS-D3/def2-TZVP + COSMO optimized geometry in THF solution. Crucial C, O, P, Cl and Fe atoms in ball-and-stick model are highlighted as grey, red, orange, green and blue balls, respectively, with most H-atoms omitted for clarity. Due to more facile electrophilic alkene addition (via bTS2), the initial phosphinidene transfer from ^{t}BuP**A** to catalyst FpCl is now rate-limiting over a barrier of 20.8 kcal/mol (increased from 19.1 by 1.7 kcal/mol due to more stable **3** than **1b**).

Table S5. Computed energies for catalytic phosphinidene transfer from dibenzo-7-phosphanorbornadiene (RP**A**) to alkenes in THF solution. TPSS-D3/def2-TZVP + COSMO computed imaginary frequency (ImF), zero-point energies (ZPE), enthalpic (Hc) and Gibbs free-energy (Gc) corrections; the COSMO-RS computed solvation enthalpic (Hsol) and Gibbs free-energy (Gsol) corrections in THF; TPSS-D3/def2-QZVP and PW6B95-D3/def2-QZVP single-point energies (TPSS-D3 and PW6B95 (E_P)); total PW6B95-D3 Gibbs free energies ($G_P = E_P + Gc + Gsol$), relative electronic energies (ΔE_T and ΔE_P) and final Gibbs free-energies (ΔG_T and ΔG_P) at the TPSS-D3 and PW6B95-D3 levels. Each structure is labeled either by its molecular formula or a specific name, with radical, singly charged cation and anion species indicated by the •, + and – superscripts, respectively. Transition structures (with only one imaginary frequency) are indicated by the "**TS**" prefix. See also main-text **Figure 5** for structural labelings. The final PW6B95-D3 Gibbs free energies are used in our discussion.

Reactions (1 mol/L in THF)	ImF cm^{-1}	ZPE kcal/mol	Hc kcal/mol	Gc kcal/mol	Hsol kcal/mol	Gsol kcal/mol	TPSS-D3 E_h	PW6B95 E_h	G_P E_h	ΔE_T kcal/mol	ΔE_P kcal/mol	*ΔG_P kcal/mol*	ΔG_T kcal/mol
Facile PR transfer from RPA (R = tBu) to FpCl complex along with anthracene (Ant) release													
FpCl + RPA	0	261.23	279.45	212.49	-38.90	-26.49	-3183.90248	-3186.30783	-3186.00540	0.00	0.00	*0.00*	0.00
Fp(RP**A**)$^+$ + Cl^-	0	262.39	281.20	220.63	-129.91	-113.48	-3183.77774	-3186.17666	-3185.99988	78.28	82.31	*3.46*	-0.57
**TS1**	160i	261.16	279.66	227.77	-28.84	-20.54	-3183.91879	-3186.30816	-3185.97489	-10.24	-0.21	*19.14*	9.11
**2**-Cl + Ant	0	262.56	280.82	214.61	-35.71	-24.21	-3183.93790	-3186.33190	-3186.02246	-22.23	-15.11	*-10.71*	-17.83
*Formation of **3a** from **2**-Cl and electron-rich styrene (CH_2=CHPh) encounters a sizeable barrier of 28.2 kcal/mol*													
**2**-Cl + CH_2=CHPh	0	223.81	240.63	177.12	-29.96	-19.68	-2953.90561	-2956.04160	-2955.78468	0.00	0.00	*0.00*	0.00
**TS2**	177i	223.49	240.39	191.11	-23.13	-15.97	-2953.89808	-2956.02192	-2955.73981	4.72	12.35	*28.16*	20.53
**3a**	0	225.74	242.06	194.05	-24.34	-17.51	-2953.93593	-2956.07160	-2955.78725	-19.02	-18.82	*-1.61*	-1.81
**TS3**	162i	223.86	240.58	191.66	-27.04	-18.58	-2953.89667	-2956.02560	-2955.74677	5.61	10.04	*23.79*	19.36
Fp(**1a**)$^+$ + Cl^-	0	224.70	241.82	184.32	-130.93	-113.41	-2953.75363	-2955.89354	-2955.77451	95.37	92.91	*6.38*	8.85
Fp(**1a**)$^+$.Cl^-	0	224.85	241.99	192.01	-49.34	-38.29	-2953.87929	-2956.02024	-2955.77226	16.52	13.40	*7.80*	10.91
FpCl + **1a**	0	224.15	240.55	177.19	-34.50	-22.92	-2953.89178	-2956.03686	-2955.78499	8.68	2.97	*-0.19*	5.51
*Formation of **3** from **2**-Cl and electron-deficient alkene CH_2=CHE (E = CO_2Me) is -5.6 exergonic over a low barrier of 17.2 kcal/mol*													
**2**-Cl + CH_2=CHE	0	200.35	216.88	154.39	-28.24	-18.36	-2950.71965	-2952.82769	-2952.60489	0.00	0.00	*0.00*	0.00
**bTS2**	182i	200.11	216.88	167.61	-23.26	-15.97	-2950.72327	-2952.82220	-2952.57754	-2.27	3.45	*17.16*	11.45
**b3**	0	200.83	217.52	168.74	-24.77	-16.95	-2950.73097	-2952.83438	-2952.58948	-7.10	-4.20	*9.67*	6.76
**bTS2c**	68i	200.38	216.72	168.55	-23.41	-16.08	-2950.73133	-2952.83335	-2952.58737	-7.33	-3.56	*10.99*	7.22
**3**	0	202.04	218.27	170.32	-23.24	-16.54	-2950.75336	-2952.86181	-2952.61374	-21.15	-21.41	*-5.56*	-5.30
**bTS3**	157i	200.63	217.08	168.72	-24.32	-16.50	-2950.72883	-2952.83427	-2952.58868	-5.76	-4.13	*10.17*	8.54

Fp**1b**$^{+}$ + Cl^{-}	0	201.03	218.10	160.54	-127.39	-111.34	-2950.57090	-2952.68301	-2952.59859	93.35	90.79	*3.95*	6.51
FpCl + **1b**	0	200.90	216.99	154.36	-32.87	-21.76	-2950.71091	-2952.82843	-2952.61109	5.49	-0.47	*-3.89*	2.06
Using dinuclear Fp$_2$ as catalyst instead													
Fp radical is not coordinated by THF, but cation Fp^{+} is tightly coordinated by one THF													
Fp$^{\bullet}$ + THF	0	135.18	145.05	96.75	-18.86	-8.33	-1916.97220	-1918.25583	-1918.11191	0.00	0.00	*0.00*	0.00
Fp$^{\bullet}$(THF)	0	136.18	146.37	110.18	-16.69	-11.89	-1916.98014	-1918.26390	-1918.10425	-4.98	-5.07	*4.81*	4.89
Fp^{+} + THF	0	135.78	145.70	97.09	-77.01	-56.26	-1916.70580	-1917.98690	-1917.91882	0.00	0.00	*0.00*	0.00
Fp(THF)$^{+}$	0	137.22	147.33	111.40	-60.90	-49.84	-1916.77952	-1918.05675	-1917.95564	-46.26	-43.83	*-23.10*	-25.53
Homolytic Fp$_2$ cleavage is much more favorable than heterolytic cleavage													
Fp$_2$	0	127.09	139.58	98.85	-20.47	-14.03	-3368.81042	-3370.86080	-3370.72263	0.00	0.00	*0.00*	0.00
Fp$^{\bullet}$ + Fp$^{\bullet}$	0	125.71	138.24	82.65	-23.01	-14.62	-3368.73668	-3370.82723	-3370.71279	46.27	21.07	*6.17*	31.37
Fp^{-} + Fp(THF)$^{+}$ - THF	0	126.72	139.59	95.84	-109.14	-99.87	-3368.59536	-3370.66713	-3370.66753	134.95	121.53	*34.58*	48.00
*Reaction of Fp$_2$ with tBuPA is -7.6 kcal/mol exergonic over a barrier of 24.2 kcal/mol via a radical path to generate **4***													
Fp$_2$ + RP**A**	0	324.15	347.49	270.16	-40.77	-28.51	-4408.04355	-4411.12748	-4410.73637	0.00	0.00	*0.00*	0.00
Fp$^{\bullet}$(RP**A**) + Fp$^{\bullet}$	0	323.10	347.18	268.80	-36.96	-25.86	-4408.00064	-4411.11589	-4410.72272	26.92	7.28	*8.56*	28.21
**TS4**$^{\bullet}$ + Fp$^{\bullet}$	200i	322.53	346.28	268.56	-36.55	-25.38	-4407.99066	-4411.09130	-4410.69775	33.19	22.70	*24.23*	34.71
Fp(PR**A**)$^{\bullet}$ + Fp$^{\bullet}$	0	323.49	347.52	269.28	-37.82	-26.11	-4408.00224	-4411.10346	-4410.70991	25.92	15.07	*16.60*	27.45
Ant + **4**	0	325.51	349.24	272.17	-38.37	-26.65	-4408.05759	-4411.14579	-4410.74850	-8.81	-11.49	*-7.61*	-4.94
*..followed by a higher barrier of 24.8 kcal/mol (via **TS5**) for styrene addition*													
**4** + CH_2=CHPh	0	286.76	309.05	234.68	-32.62	-22.12	-4178.02530	-4180.85549	-4180.51072	0.00	0.00	*0.00*	0.00
**TS5**	190i	286.84	309.02	249.68	-29.58	-20.64	-4178.01879	-4180.83918	-4180.47116	4.08	10.23	*24.82*	18.67
**7**	0	289.04	310.72	252.28	-33.87	-23.85	-4178.04656	-4180.88028	-4180.51324	-13.34	-15.56	*-1.58*	0.63
**7o**$^{\bullet}$ + Fp$^{\bullet}$	0	287.27	308.89	235.21	-34.87	-23.76	-4177.97832	-4180.82342	-4180.48044	29.48	20.12	*19.00*	28.36
**7a**$^{\bullet}$ + Fp$^{\bullet}$	0	286.11	308.20	233.39	-35.45	-23.61	-4177.98199	-4180.82553	-4180.48520	27.18	18.80	*16.02*	24.39
**TS6**$^{\bullet}$ + Fp$^{\bullet}$	174i	285.21	307.17	232.56	-33.83	-22.68	-4177.97718	-4180.82051	-4180.48002	30.19	21.95	*19.27*	27.51
Fp(**1a**)$^{\bullet}$ + Fp$^{\bullet}$	0	285.82	308.09	232.99	-33.86	-23.39	-4177.98233	-4180.83894	-4180.49890	26.96	10.38	*7.42*	24.00
Fp$^{\bullet}$ + Fp$^{\bullet}$ + **1a**	0	285.69	307.26	218.66	-38.91	-25.53	-4177.95911	-4180.82294	-4180.50613	41.53	20.42	*2.88*	23.99
Fp$_2$ + **1a**	0	287.07	308.60	234.86	-36.37	-24.94	-4178.03285	-4180.85652	-4180.51596	-4.74	-0.65	*-3.29*	-7.38
Fp(**1a**)$^{+}$ + Fp^{-}	0	286.53	308.53	233.62	-119.67	-101.57	-4177.86272	-4180.69950	-4180.48305	102.02	97.88	*17.36*	21.50
***4** is 6.3 kcal/mol more stable than seperated **5** and CO, while Fp$^{\bullet}$ radical elimination is even more difficult.*													
**4**	0	203.70	221.31	170.73	-22.80	-15.78	-3868.17141	-3870.66240	-3870.41247	0.00	0.00	*0.00*	0.00

**5o** + CO	0	201.54	219.83	157.64	-26.82	-17.02	-3868.11730	-3870.61901	-3870.38889	33.95	27.23	*14.79*	21.52
**5** + CO	0	201.53	219.59	158.28	-25.46	-16.00	-3868.14637	-3870.63520	-3870.40244	15.71	17.07	*6.29*	4.94
FpPR$^\bullet$ + Fp$^\bullet$	0	202.33	219.80	154.30	-28.86	-18.68	-3868.10347	-3870.60808	-3870.38593	42.63	34.09	*16.65*	25.19
...ionic path is prevented by a high barrier													
Fp(RPA)$^+$ + Fp$^-$	0	324.22	347.91	269.93	-118.64	-101.65	-4407.88683	-4410.98262	-4410.70842	98.34	90.90	*14.25*	17.60
**TS4i**	144i	324.64	348.45	285.98	-33.95	-24.38	-4407.99598	-4411.08095	-4410.66105	29.85	29.20	*43.97*	40.52
Ant + **4**	0	325.51	349.24	272.17	-38.37	-26.65	-4408.05759	-4411.14579	-4410.74850	-8.81	-11.49	*-10.90*	-12.32
*...while reaction of FpPR Radical from **4** and styrene encounters a additional barrier of 18.3 kcal/mol, leading to a high barrier of 36.2 kcal/mol*													
FpPR$^\bullet$ + CH_2=CHPh	0	222.54	238.42	176.92	-27.18	-17.70	-2493.58902	-2495.38755	-2495.12779	0.00	0.00	*0.00*	0.00
**TS7**$^\bullet$	200i	222.54	238.46	191.05	-24.89	-16.91	-2493.58746	-2495.37920	-2495.09868	0.98	5.24	*18.27*	14.01
FpPRSty$^\bullet$	0	224.18	239.66	193.48	-23.79	-16.21	-2493.61365	-2495.41185	-2495.12636	-15.46	-15.25	*0.90*	0.69
**TS7c**$^\bullet$	126i	225.34	241.18	193.46	-21.60	-15.13	-2493.59174	-2495.38388	-2495.09668	-1.71	2.30	*19.52*	15.51
Fp$^\bullet$ + **1a**	0	222.83	238.14	177.34	-27.40	-18.22	-2493.59077	-2495.40933	-2495.14973	-1.10	-13.67	*-13.77*	-1.20
Reaction between 4 and tBuPA is 20.8 kcal/mol endergonic to form anion FpPR$^-$													
RPA + **4**	0	400.76	429.23	342.04	-43.10	-30.25	-4907.40454	-4910.92908	-4910.42620	0.00	0.00	*0.00*	0.00
FpPR$^-$ + Fp(RPA)$^+$	0	400.35	428.92	341.40	-118.03	-99.79	-4907.26049	-4910.78415	-4910.39309	90.39	90.94	*20.78*	20.23
..with further reaction of anion FpPR$^-$ and styrene encounters an additional barrier of 26.4 kcal/mol													
FpPR$^-$ + CH_2=CHPh	0	221.02	236.94	175.29	-64.81	-55.53	-2493.64725	-2495.44721	-2495.25033	0.00	0.00	*0.00*	0.00
**TS8**$^-$	269i	219.86	235.68	188.21	-57.77	-50.16	-2493.64120	-2495.43136	-2495.20835	3.80	9.94	*26.34*	20.19
FpPRSty$^-$	0	221.09	237.27	189.45	-60.14	-52.43	-2493.65280	-2495.44542	-2495.22405	-3.48	1.12	*16.49*	11.88
**TS8c**$^-$	151i	220.44	236.42	188.84	-57.45	-50.12	-2493.65257	-2495.44615	-2495.22209	-3.34	0.66	*17.72*	13.72
Fp$^-$ + **1a**	0	221.81	237.21	175.87	-71.49	-61.95	-2493.64213	-2495.44831	-2495.26074	3.21	-0.69	*-6.53*	-2.63
TEMPO radical (or TO$^\bullet$) as a weakly oxidizing radical: it cannot oxidize the Fp$^-$ anion or neutral dimer Fp_2, and cannot even trap the Fp$^\bullet$ radical													
TO$^\bullet$ + Fp$^-$	0	226.25	239.97	182.77	-67.30	-59.21	-2168.46584	-2169.99898	-2169.79604	0.00	0.00	*0.00*	0.00
TO$^-$ + Fp$^\bullet$	0	223.77	237.76	180.51	-70.86	-61.63	-2168.42154	-2169.96793	-2169.77246	27.80	19.48	*14.80*	23.11
Fp$^\bullet$ + TO$^\bullet$	0	227.27	240.90	184.24	-23.21	-15.47	-2168.41448	-2169.96000	-2169.68503	0.00	0.00	*0.00*	0.00
FpOT	0	227.41	242.37	197.31	-17.68	-12.31	-2168.42730	-2169.95283	-2169.65499	-8.05	4.50	*18.85*	6.30
Fp$^+$ + TO$^-$	0	224.36	238.41	180.85	-129.02	-109.55	-2168.15513	-2169.69900	-2169.57937	162.74	163.78	*66.30*	65.27

Table S6: Some experimental and DFT-computed ^{31}P chemical shifts (in ppm) at the GIAO TPSS-D3/def2-QZVP level of theory using TPSS-D3/def2-TZVP + COSMO(THF) optimized geometry.

Compound	Experimental (ppm)	Calculated (ppm)	Difference (ppm)
**1a** (reference)	–165.0	–165.0	0.0
**1b**	–156.1	–158.8	–2.7
**2**-Cl	279.8	327.0	47.2
**2**-F	370.3	383.8	13.5
**b3**	–	141.0	–
**3**	244.6	269.9	25.3
**3a**	248.8	274.2	25.4
**4**	165.8	198.4	32.6
**5**	624.0	611.1	–12.9
**6**	55.2	90.5	35.3
**7**	135.2	222.9	87.7
Fp(**1a**)$^+$	–76.2	–49.6	26.6
Fp(**1b**)$^+$	–	–45.2	–
Fp(^{t}BuP**A**)$^+$	220.7	228.0	7.3

Table S7. TPSS-D3/def2-TZVP + COSMO optimized Cartesian coordinates (in Å) in THF.
Each structure is labeled by the specific name (See also **Table S5** and main-text **Figure 5**), followed by the number of atoms, the total energy, and the detailed atomic coordinates (in double-column text list). Abbreviations for substituents: R = tert-butyl $C(CH_3)_3$, E = CO_2Me, Ph = C_6H_5, and Fp = $(C_5H_5)Fe(CO)_2$.

1a : PC_2-cyclic phosphirane from styrene
30
Energy = -809.1867542035

C	0.0816049	-1.7541418	0.3953401
C	-0.5858016	-0.4026107	0.3635756
H	-0.4999084	-2.6067212	0.0533012
H	0.7128044	-1.9685574	1.2518406
H	-0.2808500	0.2751401	1.1581702
P	0.7420080	-0.6609024	-0.9425143
C	2.2706499	0.1886746	-0.2057833
C	2.0624951	1.7020429	-0.3995428
C	2.5540660	-0.1202823	1.2669055
C	3.4572479	-0.2846584	-1.0687972
H	1.8297181	1.9418173	-1.4435039
H	1.2431064	2.0717388	0.2261598
H	2.9769709	2.2425786	-0.1227748
H	2.7849339	-1.1793328	1.4187915
H	3.4244891	0.4622632	1.5958258
H	1.7150681	0.1509897	1.9169990
H	4.3732856	0.2335623	-0.7570652
H	3.6212291	-1.3626087	-0.9602938
H	3.2887860	-0.0685013	-2.1299556
C	-1.9955694	-0.2230003	-0.0631973
C	-2.7231505	0.8812661	0.4077858
C	-2.6342570	-1.1141940	-0.9407023
C	-4.0459442	1.0898262	0.0174560
H	-2.2438394	1.5806123	1.0892079
C	-3.9560434	-0.9060348	-1.3326518
H	-2.0940780	-1.9736283	-1.3295484
C	-4.6700483	0.1963932	-0.8558576
H	-4.5897767	1.9505754	0.3976154
H	-4.4302301	-1.6079486	-2.0134937
H	-5.7003087	0.3559990	-1.1609211

1b : phosphirane from methyl acrylate
26
Energy = -806.0085058477

C	0.0619922	-1.7573728	0.1107880
C	-0.5772067	-0.4133309	0.3422088
H	-0.5654446	-2.4986649	-0.3767698
H	0.6915080	-2.1396921	0.9070809
H	-0.2966549	0.1411658	1.2317374
C	-1.9610670	-0.1956397	-0.1256986
O	-2.5526751	-0.8892684	-0.9407139
O	-2.5088711	0.9011962	0.4531826
C	-3.8605589	1.2142682	0.0189484
H	-4.1344448	2.1101566	0.5742431
H	-3.8713255	1.4016189	-1.0569328
H	-4.5300481	0.3853438	0.2580991
P	0.7231054	-0.4588324	-1.0188717
C	2.2541295	0.2450900	-0.1541747
C	2.0689332	1.7733158	-0.1115999
C	2.5377944	-0.2946434	1.2503030
C	3.4289615	-0.1080576	-1.0898362
H	1.8403722	2.1752929	-1.1054123
H	1.2567169	2.0556402	0.5665274
H	2.9926698	2.2486528	0.2421911
H	2.7482277	-1.3685496	1.2347699
H	3.4219954	0.2128397	1.6567161
H	1.7091442	-0.1103329	1.9424921
H	4.3529419	0.3432699	-0.7071641
H	3.5777658	-1.1919758	-1.1491360
H	3.2598778	0.2719344	-2.1038075

2-Cl : mononuclear FpPRCl complex
30
Energy = -2643.975412357

C	2.2709046	-1.6780846	0.5063903
C	3.1108094	-0.5338212	0.2644874
C	1.3724552	-1.3448963	1.5550203
H	2.3171096	-2.6237352	-0.0149641
C	2.7179186	0.4946540	1.1565218
H	3.8925498	-0.4672825	-0.4792593
C	1.6267502	0.0032605	1.9506804
H	0.6189918	-2.0005743	1.9664325
H	3.1423369	1.4874920	1.2097268
H	1.1132285	0.5533536	2.7258542
Fe	1.0911664	-0.0304524	-0.0839256
C	1.2331027	-0.3428267	-1.8088175
O	1.3991161	-0.5556554	-2.9344235
C	0.5624318	1.6262453	-0.3081488
O	0.2859470	2.7382350	-0.4770014
P	-1.0619132	-0.8426515	-0.1973814
C	-2.2111637	-0.1307320	1.1425361
C	-2.1569731	1.3848249	1.3400853
C	-3.6398593	-0.5619378	0.7511029
C	-1.8369601	-0.8490608	2.4531290
H	-2.3588295	1.9136323	0.4038363
H	-2.9151290	1.6885960	2.0742948
H	-1.1792456	1.7001100	1.7164079
H	-3.9806761	-0.0448324	-0.1492615
H	-3.6947660	-1.6419816	0.5731525
H	-4.3233870	-0.3146816	1.5736993
H	-2.6116838	-0.6480049	3.2033155
H	-1.7799794	-1.9361367	2.3180116
H	-0.8867183	-0.4916172	2.8567512
Cl	-1.8951673	0.1962439	-1.8808149

3a : $FePC_3$-cyclic adduct of **2**-Cl and styrene

46

Energy = -2953.843290594

C	-0.4229056	3.3903811	0.0954148
C	0.8446233	3.0676526	-0.4890989
C	-1.4494800	2.7627386	-0.6695084
H	-0.5745528	3.9967402	0.9781814
C	0.5917490	2.2270615	-1.6080893
H	1.8122051	3.3918018	-0.1330791
C	-0.8222041	2.0330555	-1.7217864
H	-2.5083493	2.7863739	-0.4591365
H	1.3372177	1.7907606	-2.2582245
H	-1.3212499	1.4166930	-2.4553450
Fe	-0.1781668	1.2986909	0.1304195
C	-1.6365009	-0.0260682	0.2600977
O	-2.7657922	0.1340473	-0.1664750
C	-0.0282306	1.2709081	1.8603130
O	0.0618412	1.2910220	3.0252085
P	0.9940087	-0.4486864	-0.0974255
C	2.8270814	-0.5676280	0.2441501
C	3.3050290	-2.0268062	0.1713957
C	3.5882384	0.3015957	-0.7689925
C	3.0435644	-0.0053927	1.6629715
H	4.3817786	-2.0530827	0.3761201
H	2.8057922	-2.6534602	0.9166502
H	3.1342232	-2.4552841	-0.8197932
H	4.6585594	0.2596309	-0.5367927
H	3.4451109	-0.0558499	-1.7925449
H	3.2694799	1.3465108	-0.7104095
H	2.4902520	-0.5679657	2.4215198
H	4.1103903	-0.0784075	1.9036711
H	2.7465316	1.0451074	1.7220877
Cl	0.9061969	-1.2875873	-2.0369427
C	0.1733098	-1.7556321	0.9021727
C	-1.3159889	-1.3758330	1.0407534
H	0.3085051	-2.7345728	0.4385126
H	0.6710825	-1.7779327	1.8766624
H	-1.5043370	-1.0913862	2.0837271
C	-2.2714648	-2.4813613	0.6714020
C	-3.3470720	-2.7934751	1.5100590
C	-2.1386127	-3.1807217	-0.5357549
C	-4.2633961	-3.7876413	1.1614838
H	-3.4653060	-2.2540858	2.4471560
C	-3.0513793	-4.1734116	-0.8889188
H	-1.3183920	-2.9422834	-1.2086636
C	-4.1177485	-4.4826456	-0.0400367
H	-5.0887331	-4.0195309	1.8293844
H	-2.9327533	-4.7054187	-1.8291613
H	-4.8277031	-5.2580948	-0.3139243

7o• : radical from Fp• elimination of **7**

45

Energy = -2493.529632106

P	-1.4820119	0.3925942	1.0635747
C	-2.5377186	-0.7703639	0.0317445
C	-1.9104697	-2.1764481	0.1025566
C	-3.9911509	-0.8214453	0.5328021
C	-2.4863662	-0.2734421	-1.4233674
H	-0.8689480	-2.1655268	-0.2358930
H	-1.9416673	-2.5866821	1.1166473
H	-2.4757339	-2.8538564	-0.5501042
H	-4.4545595	0.1690343	0.5312082
H	-4.5788752	-1.4753482	-0.1252229
H	-4.0489410	-1.2313772	1.5460347
H	-3.0525910	-0.9636534	-2.0609974
H	-2.9250366	0.7215353	-1.5294101
H	-1.4548472	-0.2365571	-1.7900301
C	0.0404137	3.3714317	1.0538830
C	-0.9809094	4.3170017	0.7834690
C	-0.0988923	2.9464665	2.4146974
H	0.7621970	2.9936325	0.3433990
C	-1.7495803	4.5050173	1.9859504
H	-1.1583793	4.8100726	-0.1623893
C	-1.1878323	3.6692927	2.9902198
H	0.5393980	2.2388715	2.9241310
H	-2.5991525	5.1624300	2.1032137
H	-1.5354782	3.5823193	4.0098870
Fe	-1.8910064	2.4856909	1.3558378
C	-3.2022690	2.6366516	0.2235063
O	-4.0903431	2.8177757	-0.5101895
C	-3.1034232	1.7846817	2.7630927
O	-4.1462251	2.3205406	3.0774907
C	-1.5371283	-0.2525487	2.8021362
C	-2.6844340	0.4651393	3.5336777
H	-0.5673343	-0.0132992	3.2465371
H	-1.6565602	-1.3386503	2.8489554
H	-3.5889665	-0.1497328	3.4596204
C	-2.4641083	0.7787412	4.9967843
C	-3.5653840	0.7874596	5.8661487
C	-1.2111451	1.1395084	5.5094828
C	-3.4198337	1.1461242	7.2055372
H	-4.5444586	0.5140856	5.4824537
C	-1.0613232	1.4990429	6.8496365
H	-0.3361506	1.1410263	4.8665836
C	-2.1652615	1.5053328	7.7038216
H	-4.2862389	1.1424768	7.8614605
H	-0.0793388	1.7734434	7.2253041
H	-2.0484868	1.7822055	8.7477706

7a• : open radical from **7**•

45

Energy = -2493.532082114

P	0.7212980	1.1004769	-1.0396033
C	-0.0541940	2.3198964	0.2009961
C	-1.2221927	3.0187232	-0.5247822
C	-0.5792181	1.7101893	1.5056860
C	1.0176940	3.3857361	0.4944523
H	-0.9125868	3.4056322	-1.5020353
H	-2.0729289	2.3456458	-0.6677792
H	-1.5667883	3.8648936	0.0843471

H	0.2240249	1.2696614	2.1022877
H	-1.0567801	2.4909436	2.1138195
H	-1.3285847	0.9349798	1.3142918
H	0.5963223	4.1605595	1.1487054
H	1.8939431	2.9670765	0.9939672
H	1.3495083	3.8639998	-0.4335965
C	3.8146419	-0.3154685	-1.2904892
C	4.0900501	-1.2245907	-0.2099421
C	2.7403062	-0.8588553	-2.0464197
H	4.3327817	0.6108095	-1.4928444
C	3.1798832	-2.3089155	-0.2953856
H	4.8486042	-1.0924988	0.5492629
C	2.3308031	-2.0754891	-1.4318625
H	2.2835136	-0.3991472	-2.9113886
H	3.1187242	-3.1480581	0.3830064
H	1.5301524	-2.7225835	-1.7620417
Fe	2.1181951	-0.5062415	-0.0392312
C	2.5928761	0.5106147	1.3009343
O	2.9867623	1.0973106	2.2238068
C	0.7271947	-1.1748998	0.7783956
O	-0.1734557	-1.6720442	1.3170601
C	-0.8328555	0.0767172	-1.5384196
C	-1.3534452	0.6081684	-2.8149942
H	-1.5705696	0.1376012	-0.7317885
H	-0.5129145	-0.9636473	-1.6243793
H	-1.9809992	1.4950610	-2.7750398
C	-1.0053803	0.1324302	-4.1046734
C	-0.2015390	-1.0228654	-4.3200301
C	-1.4704337	0.8210928	-5.2628156
C	0.1203797	-1.4468394	-5.6015445
H	0.1519011	-1.5936656	-3.4675535
C	-1.1450229	0.3903290	-6.5392602
H	-2.0889390	1.7054533	-5.1276227
C	-0.3426827	-0.7462111	-6.7233981
H	0.7347273	-2.3336695	-5.7347361
H	-1.5126694	0.9386796	-7.4026515
H	-0.0868119	-1.0817538	-7.7239935

7 : $FePC_3$-cyclic adduct of **4** and styrene
60
Energy = -4177.923031537

C	2.4610206	-1.2962047	0.6751897
C	3.0250008	-0.1258509	0.0832746
C	1.6839881	-0.9077691	1.8036070
H	2.5752701	-2.3072239	0.3088718
C	2.6106938	0.9908886	0.8698501
H	3.6474489	-0.0922233	-0.7992467
C	1.7872629	0.5143712	1.9277045
H	1.1368753	-1.5746199	2.4524802
H	2.8727210	2.0232782	0.6841262
H	1.3329588	1.1249569	2.6908962
Fe	0.9095942	0.0191207	0.0748043
C	0.5480513	-1.1875054	-1.1449168
O	0.4933279	-1.9811871	-1.9894689
C	0.5367118	1.3412265	-1.0231285
O	0.3335289	2.1936994	-1.7782883
P	-1.2472747	0.3726225	0.9862101
C	-2.4871185	-0.7257370	0.0342224
C	-2.1208976	-2.2129726	0.1980527
C	-3.9263735	-0.5641915	0.5621766
C	-2.4904905	-0.3119826	-1.4471072
H	-1.0618960	-2.4163186	0.0303351
H	-2.3752424	-2.5665768	1.2019962
H	-2.6991032	-2.8076838	-0.5198169
H	-4.2855389	0.4628141	0.5011656
H	-4.5839289	-1.1912230	-0.0540104
H	-4.0220999	-0.9069179	1.5947034
H	-3.2134684	-0.9345631	-1.9885670
H	-2.7897513	0.7317178	-1.5645702
H	-1.5209639	-0.4407359	-1.9292154
C	-0.0232200	3.4525865	1.4273657
C	-1.0108211	4.2991049	0.8712142
C	-0.4099495	3.1366720	2.7720385
H	0.8654230	3.1075358	0.9228196
C	-2.0240065	4.5176098	1.8731710
H	-1.0149891	4.6996072	-0.1331212
C	-1.6397127	3.8111232	3.0415615
H	0.1496170	2.5619894	3.4934361
H	-2.9207860	5.1092659	1.7532423
H	-2.1954496	3.7645741	3.9672968
Fe	-1.9023458	2.4575001	1.4315555
C	-3.0993448	2.5759315	0.1882585
O	-3.9092349	2.7888787	-0.6302828
C	-3.0965080	1.5548260	2.6692373
O	-4.2351131	1.8988346	2.9431559
C	-1.3335295	-0.3326893	2.7150319
C	-2.5266018	0.2832655	3.4532243
H	-0.3985594	-0.0481225	3.1961838
H	-1.3772557	-1.4256905	2.7200286
H	-3.3679148	-0.4165141	3.4404616
C	-2.2991332	0.6773267	4.8984024
C	-3.4240848	0.8600991	5.7220166
C	-1.0376209	0.9314878	5.4508993
C	-3.2917126	1.2942743	7.0388011
H	-4.4108187	0.6706449	5.3105174
C	-0.8997497	1.3655066	6.7712200
H	-0.1374173	0.7818537	4.8630026
C	-2.0258685	1.5539163	7.5717632
H	-4.1787860	1.4271620	7.6526300
H	0.0927911	1.5534623	7.1717253
H	-1.9197018	1.8904209	8.5992206

3 : $FePC_3$-cyclic adduct of **2**-Cl and methyl acrylate
42
Energy = -2950.663009979

C	-0.4261380	3.3859813	-0.0929088
C	0.8750175	3.0446449	-0.5885562
C	-1.4032393	2.7217597	-0.8893752
H	-0.6307655	4.0274667	0.7535420
C	0.6908061	2.1593373	-1.6857807

H	1.8192715	3.3876751	-0.1900112
C	-0.7131587	1.9522814	-1.8724556
H	-2.4728396	2.7489175	-0.7426919
H	1.4741297	1.7018521	-2.2738642
H	-1.1666674	1.3032615	-2.6075835
Fe	-0.1751632	1.2994315	0.0425971
C	-1.6372591	-0.0022786	0.1627768
O	-2.7337386	0.0925891	-0.3576025
C	-0.0720914	1.3581562	1.7766723
O	-0.0159069	1.4397371	2.9399428
P	0.9944429	-0.4689373	-0.0732140
C	2.8095329	-0.5882863	0.3506947
C	3.2750549	-2.0535128	0.3415079
C	3.6199156	0.2419362	-0.6568844
C	2.9729782	0.0158583	1.7591566
H	4.3414118	-2.0826963	0.5944539
H	2.7374333	-2.6540185	1.0815186
H	3.1436477	-2.5092456	-0.6434131
H	4.6799951	0.1924699	-0.3831159
H	3.5104996	-0.1429512	-1.6746627
H	3.3142826	1.2922886	-0.6393379
H	2.3814290	-0.5155659	2.5113218
H	4.0276234	-0.0621367	2.0472912
H	2.6887341	1.0714229	1.7729945
Cl	0.9679686	-1.3676584	-1.9749936
C	0.1253347	-1.7300422	0.9493854
C	-1.3411054	-1.2992129	1.0635757
H	0.2093352	-2.7128745	0.4811466
H	0.6137489	-1.7645399	1.9273207
H	-1.5742422	-0.9628480	2.0784546
C	-2.2943570	-2.4021557	0.6823034
O	-2.0731856	-3.2642119	-0.1521641
O	-3.4624246	-2.2885152	1.3464947
C	-4.4915045	-3.2373754	0.9526474
H	-4.7210710	-3.1124859	-0.1075147
H	-4.1494414	-4.2570302	1.1422016
H	-5.3534426	-2.9926339	1.5713820

4 : dinuclear complex Fp_2PR
44
Energy = -3868.058987152

C	1.7796656	-1.6520104	-0.0536905
C	2.7369586	-0.7516659	-0.6321717
C	1.5222757	-1.2274398	1.2789158
H	1.3366410	-2.5058392	-0.5454821
C	3.0453573	0.2311520	0.3434994
H	3.1302547	-0.7989908	-1.6378842
C	2.2877411	-0.0571230	1.5317366
H	0.8233089	-1.6789760	1.9667685
H	3.7216689	1.0646711	0.2116948
H	2.3052180	0.5080369	2.4526651
Fe	0.9885331	0.3147360	-0.1038517
C	0.1538904	0.1521643	-1.6258926
O	-0.2962734	0.0456720	-2.6928990
C	1.1059276	2.0455897	-0.2693731
O	1.3035361	3.1791870	-0.4398425
P	-1.0539272	0.3896446	1.1604938
C	-2.3453122	-0.8081707	0.3935786
C	-1.6382065	-2.1194384	0.0054331
C	-3.2934690	-1.1439463	1.5660356
C	-3.1559226	-0.3159209	-0.8119201
H	-0.9983890	-1.9936286	-0.8707791
H	-1.0285691	-2.5049320	0.8295058
H	-2.3955750	-2.8782578	-0.2344205
H	-3.7976037	-0.2598739	1.9613334
H	-4.0640837	-1.8487128	1.2236408
H	-2.7381651	-1.6110993	2.3864234
H	-3.8522324	-1.1049617	-1.1316523
H	-3.7502864	0.5681752	-0.5741265
H	-2.5123677	-0.0735347	-1.6592381
C	-0.2515195	3.0321215	2.5091021
C	-0.6940780	4.1854162	1.7929138
C	-1.2966784	2.6287869	3.3909886
H	0.6999012	2.5356778	2.3874664
C	-2.0155440	4.5007176	2.2242727
H	-0.1319629	4.7124142	1.0360108
C	-2.3838174	3.5334622	3.2110711
H	-1.2849185	1.7653889	4.0392685
H	-2.6324174	5.3107387	1.8620916
H	-3.3365666	3.4835728	3.7206694
Fe	-1.9773568	2.5555466	1.3786825
C	-1.8648046	2.6904818	-0.3584101
O	-1.8206025	2.8231508	-1.5117872
C	-3.6648865	2.1144669	1.3212196
O	-4.8200408	1.9707085	1.3208569

5o : from **4** via direct CO elimination
42
Energy = -3754.630436367

C	1.7170017	-1.7506228	0.3768092
C	2.8614091	-0.9497724	0.0510453
C	1.0779454	-1.1733101	1.5097486
H	1.3947454	-2.6346663	-0.1542608
C	2.9172917	0.1211390	0.9836291
H	3.5489531	-1.1240262	-0.7644230
C	1.8102392	-0.0062584	1.8841145
H	0.1913161	-1.5449667	2.0022298
H	3.6536306	0.9130412	0.9958246
H	1.5690847	0.6555643	2.7024102
Fe	1.1060811	0.2211711	-0.0889157
C	0.6951394	-0.1014122	-1.7620560
O	0.4958831	-0.2809187	-2.8905819
C	1.3535690	1.9115325	-0.4819494
O	1.5746639	3.0059377	-0.7899614
P	-1.0733191	0.6962203	0.4884749
C	-2.2828803	-0.6931438	-0.0473672
C	-1.6103306	-1.9499518	-0.6160667
C	-3.0763376	-1.1313426	1.1990972
C	-3.2233235	-0.1078735	-1.1167962
H	-1.0629439	-1.7566418	-1.5393058

H	-0.9324006	-2.4111527	0.1060036
H	-2.3942057	-2.6833873	-0.8466597
H	-3.6030975	-0.3022719	1.6705406
H	-3.8153294	-1.8886687	0.9028298
H	-2.4113194	-1.5789169	1.9460163
H	-3.9698500	-0.8632328	-1.3991178
H	-3.7495286	0.7767007	-0.7516518
H	-2.6649140	0.1723610	-2.0164635
C	0.0994869	3.2522755	2.1513366
C	-0.6235680	4.1539932	1.3095047
C	-0.7148008	2.9532181	3.2937614
H	1.0884015	2.8629223	1.9618106
C	-1.8744416	4.4254129	1.9299733
H	-0.2873964	4.5481891	0.3614278
C	-1.9274556	3.6866469	3.1628584
H	-0.4571842	2.2936611	4.1108798
H	-2.6569319	5.0601519	1.5375526
H	-2.7611404	3.6630537	3.8512538
Fe	-1.6896435	2.3692232	1.5581915
C	-3.3940837	2.0817702	1.4639397
O	-4.5681873	2.0303341	1.4651536

5 : from **5o** via CO coordination
42
Energy = -3754.659347486

C	3.3545118	-0.7723638	-0.2995591
C	2.7737005	-2.0076387	0.1347383
C	2.7679697	-0.4148098	-1.5506836
H	4.1086369	-0.2069103	0.2305508
C	1.8159947	-2.3961167	-0.8448483
H	2.9946267	-2.5270750	1.0563833
C	1.8115713	-1.4176323	-1.8817918
H	2.9945152	0.4652413	-2.1343588
H	1.1825743	-3.2709818	-0.7962922
H	1.1798501	-1.4109949	-2.7572685
Fe	1.3036580	-0.5078655	-0.0153198
C	1.6206440	0.4078183	1.4306686
O	1.9165009	0.8948298	2.4452809
C	-0.0432779	-1.4211326	1.0013280
O	-0.0767031	-2.2416430	1.8625991
P	0.0912566	1.0372950	-1.1295609
C	0.0360764	2.8613210	-0.5681205
C	0.9479209	3.5686568	-1.5950426
C	-1.4165343	3.3191914	-0.8039761
C	0.4971286	3.2545079	0.8360367
H	1.9890389	3.2425640	-1.4872199
H	0.6276204	3.3562372	-2.6206671
H	0.9127734	4.6554023	-1.4362953
H	-2.0949530	2.8990467	-0.0558229
H	-1.4734270	4.4149634	-0.7442411
H	-1.7711185	3.0154820	-1.7965131
H	0.3562174	4.3364624	0.9742988
H	-0.0629017	2.7433671	1.6199754
H	1.5603286	3.0403547	0.9727797
C	-2.0528849	-2.3426156	-0.6336743
C	-3.0321006	-1.5661321	0.0385006
C	-1.6626515	-1.6479579	-1.8208681
H	-1.6542881	-3.2877184	-0.2925570
C	-3.2410323	-0.3710880	-0.7348804
H	-3.5132896	-1.8102805	0.9750688
C	-2.4081167	-0.4381561	-1.8897180
H	-0.9363034	-1.9855674	-2.5441469
H	-3.9204168	0.4328726	-0.4876139
H	-2.3310426	0.3105587	-2.6637645
Fe	-1.2655015	-0.4437255	-0.0875575
C	-1.5925462	0.5957882	1.2632395
O	-1.9057055	1.2244036	2.1924205

6 : dinulcear P-P complex $(FpPR)_2$
58
Energy = -4367.401278797

P	-1.0098685	0.2372655	0.4556175
C	-1.4607776	0.4508621	2.3147420
Fe	-2.8551560	0.7604999	-0.9629154
P	0.6645571	1.3425637	-0.4472043
C	-0.2558897	-0.0555603	3.1227305
C	-1.8438170	1.8448912	2.8211551
C	-2.6364343	-0.5123066	2.5572482
C	-1.8400946	0.2657729	-2.3108474
C	-3.3732681	-0.8739753	-0.6064667
C	-4.8209236	1.5136242	-1.1407843
C	1.3005057	2.9934832	0.3129182
Fe	2.4014490	-0.2874893	-0.5818348
H	-0.5426674	-0.1605466	4.1778585
H	0.0860381	-1.0294327	2.7599664
H	0.5751628	0.6513782	3.0749312
H	-2.7824370	2.1908469	2.3847904
H	-1.9835751	1.8117947	3.9113240
H	-1.0654045	2.5820683	2.6087153
H	-2.8741184	-0.5327472	3.6296138
H	-3.5324215	-0.1942241	2.0165700
H	-2.3870064	-1.5296774	2.2386462
O	-1.2589871	-0.0273038	-3.2691382
O	-3.7431094	-1.9579475	-0.4156100
C	-4.0154110	2.0280123	-2.1876214
C	-4.2455288	1.9236098	0.1081239
H	-5.6980119	0.8925417	-1.2601622
C	2.4246169	3.4626027	-0.6278567
C	0.1458063	4.0023752	0.2136972
C	1.8238496	2.9848130	1.7526208
C	2.8223424	0.5439880	-2.0643814
C	1.2324112	-1.3398569	-1.3686803
C	3.9629027	0.2642798	0.7180900
C	-2.9362558	2.7695247	-1.5909602
H	-4.1718584	1.8758790	-3.2465818
C	-3.0923565	2.7120871	-0.1811186
H	-4.6374959	1.6954381	1.0886201
H	2.0756485	3.5195421	-1.6640894
H	2.7624630	4.4616834	-0.3201030
H	3.2841144	2.7872661	-0.5927959

H	-0.6352257	3.7793755	0.9438229
H	0.5237836	5.0108882	0.4294206
H	-0.2954681	4.0065989	-0.7873254
H	1.0830789	2.5889047	2.4519876
H	2.7413432	2.4016152	1.8471779
H	2.0582229	4.0136560	2.0619310
O	3.1228479	1.0531477	-3.0636884
O	0.5475255	-2.1167524	-1.8875590
C	4.3962284	-0.8120817	-0.1258809
C	2.8529444	-0.2018948	1.4837450
H	4.4194105	1.2414552	0.7786210
H	-2.1400623	3.2690561	-2.1235378
H	-2.4447698	3.1774934	0.5404605
C	3.5458908	-1.9227795	0.1034244
H	5.2096739	-0.7705449	-0.8371093
C	2.5820358	-1.5418676	1.1016155
H	2.3069413	0.3626058	2.2186741
H	3.5990609	-2.8805926	-0.3952745
H	1.7842093	-2.1632641	1.4817852

Ant : anthracence
24
Energy = -539.8624752318

C	0.0567034	-3.6586197	0.7125516
C	0.0569527	-2.4758782	1.4082052
C	0.0568264	-1.2229539	0.7233103
C	0.0563672	-1.2230221	-0.7231840
C	0.0557347	-2.4759974	-1.4080277
C	0.0561116	-3.6586882	-0.7122777
C	0.0573196	0.0000483	1.4063554
C	0.0570754	-0.0000548	-1.4063445
C	0.0580309	1.2229668	-0.7232707
C	0.0578306	1.2230101	0.7232273
C	0.0589298	2.4759941	1.4080250
H	0.0589668	2.4734020	2.4953712
C	0.0600515	3.6586772	0.7122709
C	0.0602006	3.6586351	-0.7125531
C	0.0591583	2.4758892	-1.4082080
H	0.0577475	0.0000789	2.4945881
H	0.0569920	-4.6051201	1.2458087
H	0.0577091	-2.4732029	2.4955502
H	0.0548922	-2.4733754	-2.4953935
H	0.0556179	-4.6052216	-1.2454659
H	0.0571794	-0.0000966	-2.4946199
H	0.0606832	4.6052111	1.2454627
H	0.0612744	4.6051291	-1.2458092
H	0.0594230	2.4731912	-2.4955724

b3 : acyclic form of complex **3**
42
Energy = -2950.640960311

C	-4.7439002	3.0502287	0.6806074
C	-5.8607563	2.1747499	0.7639095
C	-3.7456640	2.5885675	1.6038843
H	-4.6549219	3.9011915	0.0198614
C	-5.5644301	1.1595166	1.7363818
H	-6.7686578	2.2481712	0.1811423
C	-4.2667231	1.4332194	2.2543370
H	-2.7447754	2.9874162	1.7322833
H	-6.2091474	0.3415423	2.0236528
H	-3.7526868	0.8421650	2.9990185
Fe	-4.1575395	1.1031297	0.1668993
C	-2.9869573	1.7331593	-1.0261151
O	-2.4292463	2.2786133	-1.8782542
C	-5.1412102	0.2808971	-1.0375657
O	-5.8556604	-0.1763388	-1.8252297
P	-2.8187901	-0.6102752	0.6385204
C	-2.4530677	-1.8769855	-0.7165358
C	-1.2214904	-2.7082392	-0.3141323
C	-3.6615197	-2.8189066	-0.8829328
C	-2.1967587	-1.1701157	-2.0613231
H	-1.0911275	-3.5064756	-1.0540965
H	-0.3083265	-2.1077186	-0.3012705
H	-1.3566243	-3.1746576	0.6666507
H	-3.4868010	-3.4324613	-1.7743272
H	-3.7764964	-3.4807280	-0.0230254
H	-4.5980736	-2.2758637	-1.0293171
H	-1.3686435	-0.4626888	-2.0073305
H	-1.9550329	-1.9396039	-2.8045167
H	-3.0857938	-0.6404502	-2.4145858
Cl	-3.6496034	-1.9046661	2.1360417
C	-1.2002074	-0.0958790	1.3727214
C	-0.5051417	0.6749136	0.3208993
H	-1.4260192	0.5298587	2.2413672
H	-0.6972416	-1.0087458	1.7125641
H	-0.0106808	0.1605903	-0.4934745
C	-0.3109810	2.0578855	0.4597659
O	-0.7168081	2.8078486	1.3778336
O	0.4393076	2.5961622	-0.5869065
C	0.6079300	4.0225012	-0.5317273
H	-0.3609684	4.5298955	-0.5772993
H	1.1245632	4.3228096	0.3849445
H	1.2087682	4.2768321	-1.4066825

bTS2c : almost barrierless **b3** ring-closing
42
Energy = -2950.640489697

C	-1.1221550	2.8649797	-0.1214399
C	0.2276280	3.2978365	-0.2771047
C	-1.3769261	1.8631921	-1.1141433
H	-1.8229517	3.2265983	0.6176177
C	0.8215305	2.5583131	-1.3491572
H	0.7253847	4.0434151	0.3278828
C	-0.1786663	1.6763734	-1.8589877
H	-2.2820653	1.2830245	-1.2467289
H	1.8375876	2.6496527	-1.7045141
H	-0.0460407	0.9762648	-2.6716189
Fe	0.1823125	1.2495759	0.1808565
C	-0.9728333	0.3578702	1.2596121
O	-1.6946043	0.1852166	2.1524211

C	1.2947749	1.5914451	1.4924447
O	1.9893872	1.9153095	2.3626115
P	1.0333004	-0.6869417	-0.4544449
C	2.0588120	-1.6944734	0.7657089
C	2.2412181	-3.1273557	0.2331924
C	3.4384630	-1.0339524	0.9403969
C	1.3463969	-1.7387400	2.1313523
H	2.9380254	-3.6531956	0.8960117
H	1.2987976	-3.6819269	0.2298904
H	2.6631776	-3.1338675	-0.7762882
H	3.9895904	-1.5986218	1.7012015
H	4.0144241	-1.0527079	0.0128911
H	3.3605612	0.0008031	1.2818499
H	0.3561806	-2.1924013	2.0673771
H	1.9588345	-2.3440479	2.8103088
H	1.2468245	-0.7450422	2.5743383
Cl	2.3890793	-0.5667312	-2.0729385
C	-0.2956192	-1.7903228	-1.1198437
C	-1.3600846	-1.9031847	-0.0975167
H	-0.6801912	-1.2967629	-2.0181808
H	0.1794171	-2.7330937	-1.4140723
H	-1.2323127	-2.5577896	0.7555869
C	-2.6477573	-1.4014697	-0.3725472
O	-3.0083882	-0.7257574	-1.3606100
O	-3.5638890	-1.7237929	0.6232238
C	-4.8786958	-1.1705727	0.4362764
H	-4.8573213	-0.0801672	0.5366197
H	-5.2764514	-1.4309714	-0.5482870
H	-5.4920954	-1.6081871	1.2254930

bTS2 : FLP-like alkene addition of **2-**Cl
42
Energy = -2950.630789518

C	-3.0801946	0.4855190	0.1415299
C	-3.2984681	-0.9157444	0.1510291
C	-2.1247417	0.7978760	1.1723582
H	-3.5348017	1.1934836	-0.5373874
C	-2.4723733	-1.4872335	1.1790413
H	-3.9468517	-1.4634283	-0.5187123
C	-1.7784924	-0.4188131	1.8185000
H	-1.7039009	1.7743577	1.3803818
H	-2.4002108	-2.5354104	1.4308529
H	-1.0733296	-0.5279504	2.6303890
Fe	-1.2667120	-0.5152050	-0.2446714
C	-0.7686566	0.7753326	-1.3440822
O	-0.5735429	1.6405658	-2.0855865
C	-1.2254875	-1.7828709	-1.4560190
O	-1.3054911	-2.6037196	-2.2697092
P	0.8395531	-0.8501813	0.5124986
C	2.1485408	-1.3866685	-0.7600665
C	3.5273435	-1.2164908	-0.0884540
C	2.0034605	-2.8472136	-1.2128508
C	2.0564465	-0.4611893	-1.9864576
H	4.2953020	-1.6152903	-0.7624446
H	3.7602402	-0.1658111	0.1015720
H	3.5845668	-1.7701426	0.8543515
H	2.7638382	-3.0419932	-1.9802494
H	2.1669524	-3.5408121	-0.3852507
H	1.0256723	-3.0532761	-1.6503952
H	2.0933524	0.5958145	-1.7111879
H	2.9083927	-0.6731079	-2.6448173
H	1.1419490	-0.6437168	-2.5579565
Cl	0.8482907	-2.5948320	1.7565586
C	1.7574507	1.0243978	1.5795413
C	1.9186302	2.0707996	0.6761071
H	0.9571117	1.1055746	2.3099259
H	2.6232618	0.4646300	1.9169554
H	2.7893387	2.1228448	0.0308910
C	0.9188935	3.0828697	0.5187361
O	-0.1306224	3.2173342	1.1658758
O	1.2574672	3.9851136	-0.4689969
C	0.2963381	5.0400043	-0.6806157
H	-0.6652849	4.6213633	-0.9901794
H	0.1589550	5.6254900	0.2325477
H	0.7178279	5.6582118	-1.4737676

bTS3 : PC_2 ring-conctraction of complex **3**
42
Energy = -2950.638061857

C	-0.1279243	3.1377776	-0.1862868
C	1.2890644	3.1187090	-0.0762803
C	-0.5053621	2.2606254	-1.2577959
H	-0.8040175	3.6964263	0.4460747
C	1.8026597	2.2262438	-1.0791321
H	1.8762103	3.6652897	0.6487030
C	0.6919060	1.7141902	-1.8048641
H	-1.5126328	2.0045085	-1.5566247
H	2.8399267	1.9759055	-1.2461191
H	0.7559319	0.9978816	-2.6109438
Fe	0.5231694	1.1891973	0.2337768
C	-0.8580013	0.9151847	1.3062336
O	-1.7399571	0.8382843	2.0450362
C	1.7178245	0.9587438	1.5083812
O	2.5009657	0.9310746	2.3586661
P	0.4996288	-0.9521464	-0.4572624
C	0.8772022	-2.3722318	0.7486671
C	0.5762359	-3.7148571	0.0600765
C	2.3687495	-2.3667908	1.1415775
C	0.0588236	-2.2045724	2.0422706
H	0.8888965	-4.5197396	0.7354962
H	-0.4854051	-3.8537669	-0.1555148
H	1.1459552	-3.8124187	-0.8692337
H	2.5022735	-3.1379955	1.9093831
H	3.0126670	-2.5973436	0.2936203
H	2.6857722	-1.4144773	1.5694461
H	-1.0161286	-2.2031629	1.8623056
H	0.3072975	-3.0348693	2.7144022
H	0.3249497	-1.2761524	2.5575358
Cl	2.4577557	-1.1080379	-1.7147840
C	-0.7144238	-1.4742013	-1.6795253

C -1.7236594 -1.4984374 -0.5760315
H -0.9030002 -0.6977542 -2.4226014
H -0.4693400 -2.4242879 -2.1587100
H -1.8815643 -2.4230815 -0.0377630
C -2.7961178 -0.5651617 -0.5619680
O -2.9389364 0.4355333 -1.2844314
O -3.7413409 -0.8774615 0.3983163
C -4.8339194 0.0584218 0.4958936
H -5.3814606 0.1136656 -0.4491794
H -5.4753691 -0.3307902 1.2871116
H -4.4641145 1.0535916 0.7579579

CH_2=CHE : electron-poor methyl acrylate
12
Energy = -306.6561496920
C 2.2588484 1.5421399 0.0004411
C 1.4939220 0.4470081 -0.0008078
H 1.8103370 2.5319708 0.0015726
H 3.3421568 1.4759419 0.0006619
H 1.9168396 -0.5532108 -0.0019897
C 0.0171588 0.5463631 -0.0005449
O -0.6359712 1.5788528 -0.0001905
O -0.5449583 -0.6876987 -0.0006856
C -1.9983660 -0.7026076 0.0001794
H -2.2703600 -1.7568966 0.0005419
H -2.3731982 -0.1997599 0.8941857
H -2.3742632 -0.2000412 -0.8935453

Cl^- : chloride anion
1
Energy = -460.3887073905
Cl 0.0000000 0.0000000 0.0000000

CO : carbon monoxide
2
Energy = -113.3757872552
C 0.0329496 0.0000000 0.0000000
O 1.1670504 0.0000000 0.0000000

Fp(**1a**)$^+$.Cl^- : contact ion pair
46
Energy = -2953.818729438
Fe 0.2965182 0.8075838 -1.2121573
C -0.1436435 1.8513638 -2.9702509
C 1.6620325 1.9003959 -0.9244991
C -1.7649815 1.2312821 -1.4467346
C 1.4004858 -0.5786477 -1.4097762
C -1.4029019 0.0705943 -2.1971243
C -0.4047839 0.4666513 -3.1488585
C -0.9875535 2.3282569 -1.9096517
H 0.6156177 2.3990756 -3.5118601
H -2.4878734 1.2646093 -0.6437524
H -1.8203488 -0.9193928 -2.0852049
H 0.1175398 -0.1660906 -3.8527118
H -1.0248708 3.3388228 -1.5285514
O 2.5405965 2.6334991 -0.7921275
O 2.1376882 -1.4405968 -1.5857638
P -0.1440363 0.4013147 0.9298011
Cl 2.8471308 1.0108530 -4.0978392
C 0.1926586 1.6665254 2.2773017
C 1.6984995 1.6048300 2.5918693
C -0.1980878 3.0515367 1.7314489
C -0.6209005 1.3787505 3.5477677
H 1.9702907 0.6381734 3.0273976
H 2.3120726 1.7670292 1.7018113
H 1.9450713 2.3895399 3.3163515
H -1.2621981 3.0932523 1.4761533
H -0.0040223 3.8030830 2.5052093
H 0.3835049 3.3224674 0.8472249
H -0.3271182 2.1092055 4.3103865
H -1.6949123 1.4888872 3.3745561
H -0.4249227 0.3827404 3.9557654
C -1.5390695 -0.6606959 1.4191180
C -0.1764208 -1.2830026 1.6996317
H -2.1395886 -0.3747714 2.2761382
H -2.1183007 -1.0847626 0.6048122
H 0.1269434 -1.2483646 2.7443134
C 0.3208562 -2.4825629 0.9765821
C 1.5572947 -3.0304517 1.3521933
C -0.3869354 -3.0793253 -0.0741163
C 2.0778204 -4.1354576 0.6851270
H 2.1149270 -2.5772257 2.1682439
C 0.1352496 -4.1860448 -0.7446786
H -1.3567268 -2.6922550 -0.3706238
C 1.3701792 -4.7152473 -0.3715828
H 3.0384187 -4.5433503 0.9864411
H -0.4266835 -4.6343603 -1.5590248
H 1.7782217 -5.5738521 -0.8965842

Fp(**1a**)$^\bullet$: radical complex of Fp$^\bullet$ and **1a**
45
Energy = -2493.532029495
C -1.1623526 -3.2204041 -0.6406377
C -2.4258159 -2.8163222 -0.1733466
C -0.7365097 -2.2665678 -1.6400949
H -0.5942520 -4.0754995 -0.3011026
C -2.7860722 -1.6042611 -0.8727396
H -3.0109790 -3.2991144 0.5971654
C -1.7865898 -1.3374414 -1.8437082
H 0.1902602 -2.3176264 -2.1949577
H -3.7066357 -1.0523457 -0.7380110
H -1.7663290 -0.4920601 -2.5164278
Fe -0.9693690 -1.2679283 0.2959672
C 0.5905080 -1.6193375 1.0401325
O 1.5829177 -1.9315829 1.5603454
C -1.6972397 -0.9171229 1.8554232
O -2.2165393 -0.7743620 2.8915458
P -0.4251863 1.0341252 -0.2601297
C -1.3276399 2.5119872 0.5083475
C -0.8439775 2.6115146 1.9663420

C	-1.0949802	3.8444286	-0.2121655
C	-2.8289931	2.1702016	0.4668884
H	-0.9729130	1.6642997	2.4986371
H	0.2146202	2.8885777	2.0106279
H	-1.4227329	3.3791797	2.4956009
H	-1.4995058	3.8294796	-1.2287061
H	-1.6116954	4.6394054	0.3408290
H	-0.0351193	4.1152141	-0.2589662
H	-3.3991300	2.9642410	0.9653634
H	-3.1863078	2.0895294	-0.5657277
H	-3.0393647	1.2257086	0.9764879
C	1.2824138	1.6739453	-0.6775108
H	1.4715701	2.6697897	-0.2816187
C	0.3090896	1.6278105	-1.8377197
H	-0.0109342	2.5801553	-2.2485897
H	0.4667902	0.8612678	-2.5920147
C	2.4517361	0.7659368	-0.5741684
C	3.4256231	1.0174371	0.4055548
C	2.6163564	-0.3539229	-1.4012373
C	4.5230449	0.1737983	0.5590592
H	3.3134520	1.8831412	1.0545930
C	3.7134315	-1.2011067	-1.2476805
H	1.8841899	-0.5703876	-2.1725736
C	4.6708883	-0.9442694	-0.2659412
H	5.2638384	0.3876422	1.3249541
H	3.8190091	-2.0652773	-1.8982357
H	5.5243111	-1.6055345	-0.1456513

Fp(**1a**)$^{+}$: cation complex of Fp^{+} and **1a**
45
Energy = -2493.408821400

C	-0.9962883	-2.9511246	-0.8083938
C	-2.1996277	-2.7723993	-0.0716343
C	-0.8854408	-1.8838287	-1.7553975
H	-0.2761186	-3.7439611	-0.6597477
C	-2.8531393	-1.5960717	-0.5767260
H	-2.5568642	-3.4061659	0.7276197
C	-2.0470103	-1.0605461	-1.6157827
H	-0.0856754	-1.7496603	-2.4685338
H	-3.7909355	-1.1894946	-0.2258241
H	-2.2670748	-0.1742246	-2.1936182
Fe	-0.9508632	-1.1070507	0.1936795
C	0.6381566	-1.5467953	0.8641579
O	1.6034917	-1.9268686	1.3586897
C	-1.6061368	-0.7917710	1.8070800
O	-2.0593688	-0.6777567	2.8607479
P	-0.4003785	1.0133759	-0.2424871
C	-1.3694923	2.4532617	0.4729655
C	-0.8996784	2.6418476	1.9266511
C	-1.1268796	3.7416188	-0.3281063
C	-2.8647533	2.0940500	0.4166498
H	-1.0232100	1.7343441	2.5234455
H	0.1521997	2.9408348	1.9652254
H	-1.4985886	3.4318130	2.3935884
H	-1.5150386	3.6652720	-1.3472534
H	-1.6618718	4.5548986	0.1750462
H	-0.0695333	4.0195542	-0.3659390
H	-3.4417052	2.9256760	0.8361254
H	-3.1994907	1.9355597	-0.6137446
H	-3.0958133	1.2002176	1.0016643
C	1.3103796	1.5662115	-0.6753553
H	1.4996958	2.5802414	-0.3284047
C	0.3327159	1.4706630	-1.8419509
H	0.0228201	2.4013813	-2.3053145
H	0.4822295	0.6626946	-2.5507641
C	2.4787944	0.6581324	-0.5348564
C	3.3572665	0.8547102	0.5420807
C	2.7309300	-0.3915794	-1.4270945
C	4.4501232	0.0136142	0.7292046
H	3.1748094	1.6704308	1.2373859
C	3.8263327	-1.2354527	-1.2392286
H	2.0862323	-0.5484264	-2.2861438
C	4.6860210	-1.0396377	-0.1590863
H	5.1192365	0.1785946	1.5686748
H	4.0086470	-2.0428001	-1.9425258
H	5.5377819	-1.6970406	-0.0125673

Fp(**1b**)$^{+}$: cation complex of Fp^{+} and **1b**
41
Energy = -2490.227042149

C	-0.6798994	-2.9211889	-0.6550353
C	-1.9740945	-2.8863918	-0.0620972
C	-0.6002110	-1.8784639	-1.6285807
H	0.1185947	-3.6004192	-0.3900341
C	-2.7154922	-1.8292081	-0.6943023
H	-2.3354222	-3.5392456	0.7196025
C	-1.8714825	-1.2171668	-1.6566369
H	0.2681281	-1.6392715	-2.2243729
H	-3.7331014	-1.5432155	-0.4680435
H	-2.1396904	-0.3829719	-2.2895074
Fe	-0.9969019	-1.0608682	0.2620201
C	0.5103494	-1.2779391	1.1697452
O	1.4737193	-1.4434691	1.7760760
C	-1.9342970	-0.7247201	1.7290203
O	-2.5653131	-0.6114635	2.6863126
P	-0.4796191	1.0467289	-0.2477625
C	-1.3526230	2.5137004	0.5239333
C	-0.9231865	2.5772595	2.0003013
C	-0.9977784	3.8299472	-0.1842181
C	-2.8653329	2.2580372	0.3862288
H	-1.1299970	1.6461501	2.5343784
H	0.1444765	2.7992419	2.0906035
H	-1.4839599	3.3764451	2.4971341
H	-1.3402383	3.8405785	-1.2220861
H	-1.5131640	4.6392830	0.3447672
H	0.0727075	4.0497828	-0.1533766
H	-3.4029352	3.0923345	0.8496047
H	-3.1644947	2.2019077	-0.6653561
H	-3.1797242	1.3399277	0.8880072
C	1.2322393	1.5750011	-0.7030444

H	1.5215872	2.5569392	-0.3435209
C	0.2274542	1.5012555	-1.8507545
H	-0.0725576	2.4390823	-2.3050447
H	0.3972145	0.6874275	-2.5494992
C	2.3175872	0.5580081	-0.6839876
O	2.3040521	-0.4836593	-1.3220173
O	3.3101625	0.9291910	0.1415113
C	4.4254544	-0.0073211	0.2362344
H	4.8829013	-0.1307510	-0.7468217
H	5.1172539	0.4536046	0.9382292
H	4.0664216	-0.9679561	0.6088248

Fp_2 : dinuclear iron-complex
30
Energy = -3368.721409645

C	3.2780134	-0.4322224	0.7910860
C	2.8307289	0.8883610	0.4724110
C	2.5778385	-0.8806912	1.9542811
H	4.0166683	-0.9988803	0.2407457
C	1.8437254	1.2488182	1.4345286
H	3.1575692	1.4889988	-0.3642678
C	1.6879743	0.1611738	2.3454997
H	2.6804681	-1.8458401	2.4292141
H	1.2889753	2.1766168	1.4572189
H	0.9954755	0.1259377	3.1751382
Fe	1.2369473	-0.4973537	0.3639888
C	1.4988994	-1.6218237	-0.9473054
O	1.6956522	-2.3704106	-1.8157926
C	-0.1619393	-1.5608093	1.1270232
O	-0.2871639	-2.4700158	1.8806797
C	-1.8627558	-2.1577120	-1.0160253
C	-2.8500576	-1.7973828	-0.0541808
C	-1.7069073	-1.0701035	-1.9270317
H	-1.3078451	-3.0854155	-1.0386342
C	-3.2974403	-0.4768852	-0.3730665
H	-3.1770499	-2.3979931	0.7824616
C	-2.5970487	-0.0283801	-1.5361097
H	-1.0142225	-1.0348885	-2.7565094
H	-4.0363157	0.0896936	0.1770516
H	-2.6997034	0.9367032	-2.0111577
Fe	-1.2564521	-0.4114492	0.0545678
C	-1.5186898	0.7130989	1.3657574
O	-1.7155514	1.4618144	2.2341029
C	0.1424341	0.6522766	-0.7082166
O	0.2675913	1.5615214	-1.4617821

$Fp_2^{\bullet+}$: radical cation
30
Energy = -3368.526195758

C	3.2699648	-0.4858853	0.8160146
C	2.8412834	0.8250072	0.4272533
C	2.5461812	-0.8652534	1.9803721
H	4.0009994	-1.0923476	0.2993744
C	1.8666971	1.2604619	1.3745396
H	3.1929753	1.3809413	-0.4302506
C	1.6769431	0.2217986	2.3258794
H	2.6288297	-1.8069091	2.5038512
H	1.3448261	2.2073527	1.3578598
H	0.9901848	0.2408908	3.1609993
Fe	1.2198075	-0.5173192	0.3816936
C	1.5602611	-1.5427392	-1.0290147
O	1.8059066	-2.2262455	-1.9225820
C	0.0399155	-1.7163064	1.0923675
O	-0.2728158	-2.6267935	1.7531079
C	-1.8859749	-2.1705085	-0.9528216
C	-2.8621255	-1.7310156	-0.0090824
C	-1.6926787	-1.1349409	-1.9067583
H	-1.3651693	-3.1178963	-0.9318632
C	-3.2886324	-0.4207734	-0.4030407
H	-3.2167048	-2.2839866	0.8491479
C	-2.5617299	-0.0459439	-1.5667612
H	-1.0041341	-1.1572953	-2.7403002
H	-4.0202511	0.1881337	0.1098726
H	-2.6420023	0.8941461	-2.0934192
Fe	-1.2396771	-0.3904054	0.0364566
C	-1.5830381	0.6318352	1.4488151
O	-1.8304302	1.3129792	2.3436644
C	-0.0617918	0.8118663	-0.6709364
O	0.2521989	1.7239098	-1.3287620

FpCl : mononuclear iron-complex
16
Energy = -2144.617452933

C	1.9578353	-1.5363956	0.9362132
C	2.4812611	-1.2767279	-0.3595838
C	2.2504573	-0.4329053	1.7831246
H	1.3555993	-2.3911273	1.2080045
C	3.1229060	0.0102726	-0.3158872
H	2.4069483	-1.9250800	-1.2207581
C	2.9810035	0.5290444	1.0015841
H	1.9717560	-0.3330283	2.8222573
H	3.6172682	0.5042368	-1.1407734
H	3.3491809	1.4844629	1.3486270
Fe	1.1095120	0.2443419	0.1435210
C	0.7306656	0.7589944	-1.5057460
O	0.5113323	1.0914615	-2.5883841
C	0.4727233	1.7092140	0.9029422
O	0.0835258	2.6691205	1.4108236
Cl	-0.9479153	-0.7732742	0.3229029

FpFp(**1a**) : adduct of **1a** and Fp_2
60
Energy = -4177.887251735

C	-2.6253633	2.5100424	-1.7591565
C	-2.0786186	1.5208122	-2.6197161
C	-4.0526324	2.3309170	-1.7142106
H	-2.0721901	3.2847743	-1.2466708
C	-3.1451688	0.6966586	-3.0736628
H	-1.0334297	1.3846172	-2.8528239
C	-4.3672180	1.2092103	-2.5173437

H	-4.7490331	2.9214333	-1.1355407
H	-3.0506827	-0.1658957	-3.7163696
H	-5.3540642	0.7947047	-2.6717444
Fe	-3.0629122	0.5820643	-0.9626109
C	-3.0729189	1.2118332	0.6661025
C	-3.9690452	-0.8657037	-0.5904593
O	-3.2198297	1.7462029	1.6918898
O	-4.6086210	-1.8060219	-0.3406920
C	-1.4282956	-0.8213950	-0.8893377
Fe	-0.5553558	-1.6808129	0.6966516
O	-1.1415570	-1.1542311	-2.0130911
C	-0.7145268	-3.1673007	-0.1851010
P	1.3848560	-1.1351528	-0.1562225
O	-0.8665818	-4.1967404	-0.7131071
C	1.7208730	0.1821795	-1.3562326
C	2.2171476	0.5216355	0.0465756
C	2.7365291	-2.4237446	-0.4338710
H	0.8488762	0.7165933	-1.7174562
H	2.4797622	0.0218837	-2.1177754
C	1.6100031	1.5944936	0.8698622
H	3.2929689	0.4452687	0.1752324
C	2.4139872	-3.1174406	-1.7713914
C	4.1432260	-1.8130570	-0.4996972
C	2.6821515	-3.4421987	0.7158309
C	2.2716793	2.0081570	2.0378345
C	0.3943653	2.2089424	0.5402205
H	1.4092619	-3.5469904	-1.7738683
H	2.4907840	-2.4164148	-2.6083338
H	3.1324406	-3.9301493	-1.9357340
H	4.4416429	-1.3784306	0.4591647
H	4.8552797	-2.6112716	-0.7421386
H	4.2304611	-1.0488458	-1.2787060
H	3.4454702	-4.2118431	0.5496460
H	2.8833620	-2.9651699	1.6810376
H	1.7065557	-3.9316230	0.7687599
C	1.7361525	3.0040606	2.8516225
H	3.2162904	1.5403802	2.3063450
H	-0.1582053	1.8839978	-0.3355840
C	-0.1380843	3.2121261	1.3494300
C	0.5265272	3.6137511	2.5090200
H	2.2641819	3.3068800	3.7516712
H	-1.0842681	3.6734271	1.0829539
H	0.1070267	4.3927417	3.1389189
C	-1.5608201	-2.5702498	2.3123679
C	-2.0192582	-1.2330248	2.1429560
C	-0.1640961	-2.5308722	2.6328495
H	-2.1669264	-3.4619437	2.2272133
C	-0.9097147	-0.3584667	2.3249264
H	-3.0345226	-0.9519149	1.9142074
C	0.2309536	-1.1698058	2.6260617
H	0.4728988	-3.3843267	2.8131586
H	-0.9156132	0.7179619	2.2714416
H	1.2301634	-0.7970939	2.8052976

FpFpRP**A** : adduct of RP**A** and Fp_2

68

Energy = -4407.892482865

C	-3.1146274	2.6126680	-1.5559906
C	-2.5248501	1.6952227	-2.4675321
C	-4.4542040	2.1739312	-1.2748481
H	-2.6419750	3.4957365	-1.1497130
C	-3.4640354	0.6610303	-2.7232758
H	-1.5132069	1.7281966	-2.8391988
C	-4.6627870	0.9695534	-1.9884170
H	-5.1552258	2.6528789	-0.6058845
H	-3.3084644	-0.2013739	-3.3552538
H	-5.5617093	0.3681770	-1.9677734
Fe	-3.0246089	0.6920179	-0.6566338
C	-2.6276622	1.4109998	0.8896295
O	-2.4578511	1.9657517	1.8978171
C	-3.7402614	-0.7995842	-0.0883680
O	-4.2938569	-1.7674205	0.2471131
C	-1.7860538	-1.1464186	2.2871866
C	-1.6515909	-2.5466099	2.0938185
C	-0.5148157	-0.6114720	2.6261482
H	-2.7038315	-0.5922230	2.1909234
C	-0.2892411	-2.9028990	2.3720480
H	-2.4423682	-3.2273493	1.8116765
C	0.4069647	-1.7155670	2.6907590
H	-0.2924285	0.4235569	2.8334789
H	0.1347997	-3.8949854	2.3110827
H	1.4552941	-1.6540853	2.9465997
Fe	-0.4015065	-1.5414464	0.7144793
C	-0.6491904	-2.7874359	-0.4660608
O	-0.8919391	-3.6602118	-1.2040147
C	-1.2042451	-0.3908110	-0.7411185
O	-0.7321525	-0.4014986	-1.8537945
C	0.0762026	3.6651586	0.3928424
C	0.4546249	2.7392150	-0.5879829
C	1.2438461	1.6587386	-0.2178660
C	1.6504544	1.4967638	1.1198393
C	1.2763193	2.4139328	2.0940881
C	0.4795031	3.5036229	1.7189631
C	1.8800351	0.5883330	-1.0923211
C	2.5918045	0.3055422	1.2326352
C	3.7824051	0.6697230	0.3650987
C	3.3737602	0.8121578	-0.9752526
C	4.2943081	1.1269720	-1.9667378
H	3.9842895	1.2266384	-3.0035768
C	5.6383677	1.3025617	-1.6051529
C	6.0391541	1.1773697	-0.2728705
C	5.1080498	0.8631094	0.7275043
H	1.4749279	0.4942881	-2.0949694
H	-0.5382387	4.5185206	0.1192769
H	0.1499390	2.8742707	-1.6209075
H	1.5990899	2.2956682	3.1253506
H	0.1720862	4.2277474	2.4678894
H	2.8258798	-0.0196677	2.2447852
H	6.3737186	1.5403857	-2.3687605
H	7.0835156	1.3195902	-0.0093636

H	5.4264308	0.7504701	1.7606192
P	1.6245550	-0.9009675	0.0850900
C	2.7549849	-2.3175856	-0.5078362
C	2.3917437	-2.5176504	-1.9933534
C	4.2792818	-2.1365825	-0.3839012
C	2.4096741	-3.5815770	0.3037303
H	1.3114174	-2.5745041	-2.1523168
H	2.7858084	-1.6964595	-2.6004054
H	2.8399515	-3.4533960	-2.3493338
H	4.5822758	-1.9520441	0.6502187
H	4.7429519	-3.0785353	-0.7053352
H	4.6710418	-1.3365669	-1.0117118
H	3.0273228	-4.4075019	-0.0697321
H	2.6395497	-3.4446805	1.3660328
H	1.3626412	-3.8663301	0.2130664

FpOT : unstable radical recombination
44
Energy = -2168.350155152

C	-10.3739120	-1.3377680	-0.0521023
C	-11.6168455	-1.8398364	-0.8069572
C	-11.5724666	-3.3381351	-1.0923844
C	-10.3257583	-3.6406300	-1.9181657
C	-9.0185817	-3.2119653	-1.2264120
H	-12.5002775	-1.5723420	-0.2148051
H	-11.5631292	-3.9090960	-0.1555544
H	-12.4710713	-3.6463448	-1.6394563
H	-10.2484264	-4.7106339	-2.1462941
H	-10.3982001	-3.1076114	-2.8756911
H	-11.6856945	-1.2980379	-1.7600121
N	-9.1533433	-1.7811737	-0.7980545
O	-8.0175112	-1.3596052	-0.1300638
C	-10.3871371	-1.8019614	1.4193736
H	-9.4092676	-1.6074137	1.8682269
H	-11.1461343	-1.2374294	1.9725936
H	-10.6178753	-2.8646617	1.5225899
C	-10.3847352	0.1964215	-0.0701932
H	-10.2371242	0.5688416	-1.0889150
H	-11.3512314	0.5565341	0.2993265
H	-9.6012567	0.5987637	0.5740728
C	-8.6661018	-4.1543026	-0.0564306
H	-8.3434090	-5.1240815	-0.4513952
H	-7.8467645	-3.7206866	0.5238348
H	-9.5131842	-4.3279794	0.6108250
C	-7.8913315	-3.2886119	-2.2634252
H	-7.8310044	-4.3098670	-2.6554956
H	-8.0878017	-2.6063681	-3.0955725
H	-6.9304536	-3.0313911	-1.8162114
C	-4.9770542	-1.8221488	-0.2206893
C	-4.7193278	-1.4823856	-1.5719595
C	-4.9099806	-0.6344281	0.5628543
H	-5.2604921	-2.8003285	0.1436046
C	-4.4504625	-0.0691571	-1.6338452
H	-4.7105157	-2.1619582	-2.4137949
C	-4.5512762	0.4494222	-0.3162241
H	-5.0484364	-0.5633054	1.6331730
H	-4.1984464	0.4924302	-2.5232249
H	-4.3821522	1.4762234	-0.0203276
Fe	-6.7168055	-0.1852570	-0.9241978
C	-7.4685285	0.0467087	-2.4849578
O	-7.9360910	0.2518568	-3.5264833
C	-7.3035891	1.3379649	-0.3392214
O	-7.6233931	2.3973142	0.0173395

FpPR**A**• : radical complex of FpPR• and **A**
53
Energy = -2723.541203580

C	2.4739632	0.9692910	-0.0261307
C	3.1243374	1.3430152	1.1860005
C	3.0064884	-0.2906319	-0.4395192
H	1.6973924	1.5285766	-0.5274397
C	4.0602348	0.3229504	1.5310924
H	2.9210055	2.2370106	1.7576499
C	3.9832091	-0.6854810	0.5190925
H	2.6955421	-0.8578126	-1.3041974
H	4.6989367	0.3061879	2.4027161
H	4.5526680	-1.6047938	0.4988863
Fe	2.1074718	-0.4843233	1.4694288
C	1.2609111	0.0406669	2.8988652
O	0.7466125	0.3512071	3.8940424
C	2.3400052	-2.0533338	2.2017443
O	2.6171841	-3.0303741	2.7673204
C	0.5125187	3.7887661	0.8198460
C	-0.0239902	2.6416646	1.4152754
C	-0.7747861	1.7345182	0.6712474
C	-1.0665249	2.0206018	-0.6973391
C	-0.4750684	3.1589789	-1.2950959
C	0.3110210	4.0263814	-0.5467640
C	-1.2415070	0.4091130	1.1955025
C	-2.0205751	1.2136696	-1.3846646
C	-2.9149884	0.3824571	-0.6518711
C	-2.6159947	0.0893089	0.7120970
C	-3.5835512	-0.5076384	1.5183089
H	-3.3669122	-0.6855963	2.5687968
C	-4.8126240	-0.9038403	0.9837548
C	-5.0733815	-0.7145136	-0.3821236
C	-4.1425434	-0.0740342	-1.1887197
H	-1.1834074	0.3477452	2.2832175
H	1.0956437	4.4857960	1.4149340
H	0.1528590	2.4518537	2.4693677
H	-0.6721210	3.3642751	-2.3446962
H	0.7506494	4.9017424	-1.0167746
H	-2.2124568	1.4026142	-2.4385109
H	-5.5559436	-1.3737446	1.6211746
H	-6.0168594	-1.0489887	-0.8046144
H	-4.3632618	0.1168033	-2.2363369
P	0.0517686	-0.8573512	0.3638812
C	-0.6012253	-2.6188027	0.6727448
C	-0.9326248	-2.9616869	2.1285825
C	-1.8393488	-2.8449571	-0.2259104

C	0.4837879	-3.5593048	0.1069281
H	-0.0661701	-2.8348379	2.7830573
H	-1.7398090	-2.3271486	2.5057433
H	-1.2639138	-4.0067745	2.2068845
H	-1.6420883	-2.5084654	-1.2492557
H	-2.0553826	-3.9218472	-0.2551013
H	-2.7280254	-2.3324962	0.1386714
H	0.1063884	-4.5901102	0.1305804
H	0.7048143	-3.3047356	-0.9363754
H	1.4179288	-3.5310189	0.6666965

FpPROT : stable complex of FpPR• and TO•
58
Energy = -2667.738221476

C	1.1745123	1.8556593	-0.4246976
C	2.4624548	2.2410543	0.0819828
C	1.3582943	0.6841136	-1.2179637
H	0.2248230	2.3309036	-0.2245976
C	3.4246721	1.3082796	-0.3831212
H	2.6594116	3.0821762	0.7328219
C	2.7374471	0.3370387	-1.1872261
H	0.5776418	0.1474898	-1.7279528
H	4.4813834	1.3128936	-0.1553358
H	3.1913869	-0.5126257	-1.6780341
Fe	1.9379307	0.3156556	0.7672411
C	1.6865788	0.9048137	2.3975775
O	1.6043385	1.3124118	3.4818677
C	2.9493288	-0.9885638	1.3494206
O	3.7179155	-1.7607619	1.7579762
P	-0.0580094	-0.9388820	1.0185890
C	-0.0169953	-2.5728962	0.0420874
C	1.0879042	-3.4482425	0.6531402
C	-1.3787266	-3.2410475	0.3330979
C	0.1864070	-2.4594066	-1.4713009
H	2.0771706	-3.1531711	0.2971869
H	1.0821374	-3.3998884	1.7492625
H	0.9238932	-4.4942333	0.3634038
H	-2.2042378	-2.6596335	-0.0847870
H	-1.3945930	-4.2439153	-0.1144781
H	-1.5415360	-3.3440835	1.4119705
H	0.0988043	-3.4525654	-1.9329933
H	-0.5701800	-1.8114301	-1.9244412
H	1.1801759	-2.0683393	-1.7083969
N	-1.9518907	0.9908310	0.1580302
O	-1.2694566	-0.3169932	-0.0003419
C	-2.6512179	1.0999146	1.4898008
C	-3.5342567	2.3686531	1.4414866
C	-4.4788193	2.4321377	0.2500984
C	-3.6404865	2.3397303	-1.0180981
C	-2.8076258	1.0458617	-1.0826355
H	-4.0791655	2.4117777	2.3917286
H	-5.2136641	1.6193206	0.2900048
H	-5.0445754	3.3709077	0.2671508
H	-4.2643258	2.3726539	-1.9189106
H	-2.9571963	3.1985273	-1.0611549
H	-2.8697787	3.2424062	1.4102258
C	-3.4675384	-0.1354470	1.9080581
H	-2.8731549	-1.0436223	1.7791847
H	-3.7258475	-0.0442824	2.9687816
H	-4.3958532	-0.2404964	1.3439645
C	-1.5905810	1.3799288	2.5663062
H	-0.9047432	2.1589726	2.2230560
H	-2.0920673	1.7296883	3.4762589
H	-1.0192077	0.4834250	2.8169659
C	-3.7258694	-0.1726363	-1.3118748
H	-4.1270194	-0.1203989	-2.3300639
H	-3.1567331	-1.0987692	-1.2135180
H	-4.5709295	-0.2062395	-0.6228593
C	-1.8721389	1.1595960	-2.2947613
H	-2.4674626	1.3981650	-3.1827949
H	-1.1378017	1.9554220	-2.1450894
H	-1.3505831	0.2186097	-2.4819652

FpPRSty⁻ : adduct of FpPR⁻ and styrene
45
Energy = -2493.618973931

Fe	2.0269318	0.4250328	1.0424601
C	3.9603329	-0.2109984	1.6587486
H	4.5081026	0.2368036	2.4764203
C	3.0324749	-1.3017943	1.7621242
H	2.7816700	-1.8319405	2.6698175
C	2.5240817	-1.5818244	0.4606552
H	1.8132368	-2.3529822	0.2091500
C	3.1008023	-0.6491269	-0.4431241
H	2.9143528	-0.5877427	-1.5060361
C	3.9935122	0.1989966	0.3037311
H	4.5771203	1.0138350	-0.1024945
C	1.5163882	1.0598243	2.5798579
O	1.3024038	1.5293465	3.6299453
C	1.7310763	1.9352202	0.2210116
O	1.5797176	2.9547950	-0.3208129
P	-0.3058532	-0.0063821	0.3382690
C	-1.2069470	-1.0625607	1.6665207
C	-2.4748170	-1.6680137	1.0337312
H	-3.0864284	-0.8971727	0.5544999
H	-2.2336274	-2.4252732	0.2819953
H	-3.0712198	-2.1518811	1.8221677
H	-2.2550815	0.7069288	2.3884068
C	-1.6575193	-0.1175541	2.7925447
H	-0.8095862	0.3087260	3.3326144
H	-2.2759782	-0.6694983	3.5159604
C	-0.3607129	-2.1948385	2.2578760
H	-0.0165331	-2.8864713	1.4803575
H	0.5140918	-1.7949048	2.7773543
H	-0.9546093	-2.7765329	2.9791507
C	-0.2263343	-1.2574448	-1.0978338
H	0.7375286	-1.1273702	-1.6036018
H	-0.2533132	-2.2767591	-0.6947379
C	-1.3797827	-0.9594495	-1.9780052
H	-2.3109451	-1.4971779	-1.8190465

C	-1.3555516	0.0683135	-2.9354844
C	-0.2191040	0.9300729	-3.1313279
H	0.6428409	0.8265671	-2.4775587
C	-0.2086029	1.9215170	-4.1038810
H	0.6745720	2.5527279	-4.1996158
C	-1.3083005	2.1374328	-4.9502037
H	-1.2918284	2.9166226	-5.7073255
C	-2.4413994	1.3176096	-4.7730403
H	-3.3157528	1.4645511	-5.4067186
C	-2.4705415	0.3269135	-3.8057299
H	-3.3639106	-0.2874092	-3.6934777

FpPRSty• : adduct of FpPR• and styrene
45
Energy = -2493.532094808

Fe	0.7784860	-0.3187608	1.6400904
C	2.6015694	0.4385672	2.3728231
H	2.7538670	0.7633810	3.3928759
C	2.7963254	-0.8778439	1.8821875
H	3.1219241	-1.7333009	2.4569425
C	2.4530038	-0.8768379	0.4861025
H	2.4932220	-1.7335466	-0.1720711
C	2.0692992	0.4456901	0.1267781
H	1.7338904	0.7662125	-0.8493736
C	2.1403437	1.2629941	1.2877585
H	1.8950213	2.3137270	1.3441842
C	0.0849715	-1.9183879	1.7416164
O	-0.3225177	-3.0033105	1.8157497
C	-0.0543883	0.2841527	3.0538620
O	-0.5055314	0.6591966	4.0572687
P	-0.9132880	0.2894423	0.1231496
C	-2.6594179	0.2932891	0.8826723
C	-3.6607282	0.3054520	-0.2906924
H	-3.4160506	1.0878749	-1.0177573
H	-3.6916877	-0.6576362	-0.8093863
H	-4.6672756	0.5045806	0.1002312
H	-2.6229972	2.4728125	0.9610345
C	-2.8064164	1.6289153	1.6350424
H	-2.1161149	1.7095161	2.4774313
H	-3.8278293	1.7188340	2.0281409
C	-2.9949799	-0.8842715	1.8049514
H	-2.8760709	-1.8437878	1.2910093
H	-2.3653676	-0.8945802	2.6987286
H	-4.0407470	-0.8110069	2.1342712
C	-1.0388858	-1.2487724	-1.0300975
H	-0.0848815	-1.7755729	-0.9632699
H	-1.8199268	-1.9143343	-0.6486639
C	-1.3229125	-0.7779316	-2.4016963
H	-2.3528054	-0.5319853	-2.6482598
C	-0.3469866	-0.4967803	-3.3914700
C	1.0390569	-0.7755705	-3.2227743
H	1.3807272	-1.2492146	-2.3082773
C	1.9598783	-0.4738589	-4.2161109
H	3.0116224	-0.6992617	-4.0590556
C	1.5463074	0.1137257	-5.4192643
H	2.2714185	0.3494576	-6.1925271
C	0.1842939	0.3892405	-5.6154129
H	-0.1475610	0.8413057	-6.5464595
C	-0.7418340	0.0918015	-4.6281945
H	-1.7951832	0.3125388	-4.7853151

FpPR⁻ : anion complex
29
Energy = -2183.779467233

C	2.1173941	2.1572481	-0.5013397
C	3.2336536	2.0088105	0.3867505
C	2.4163679	1.4548149	-1.7170098
H	1.1966618	2.6833019	-0.2867733
C	4.2072600	1.2132836	-0.2918672
H	3.3254826	2.4305398	1.3781344
C	3.7084767	0.8913432	-1.6033162
H	1.7461468	1.3451910	-2.5580972
H	5.1624790	0.9041964	0.1125626
H	4.2127057	0.2880177	-2.3457877
Fe	2.4332255	0.1415096	-0.0309593
C	1.0967744	-0.0349327	1.2397391
O	0.8381138	0.0810543	2.4242674
C	3.2008257	-1.3248856	0.4522164
O	3.7972427	-2.2898378	0.7842119
P	0.2536640	-0.5394895	-0.3394036
C	-0.0616640	-2.4255139	-0.1579911
C	0.2696724	-2.9689392	1.2364097
C	-1.5652050	-2.6199628	-0.4290971
C	0.7469688	-3.1760909	-1.2257478
H	1.3312428	-2.8536954	1.4692510
H	-0.3049481	-2.4483075	2.0091030
H	0.0254303	-4.0412421	1.2868933
H	-1.8411333	-2.2396896	-1.4198884
H	-1.8244055	-3.6884888	-0.3868029
H	-2.1701571	-2.0906913	0.3161005
H	0.5094029	-4.2510263	-1.2014699
H	0.5151467	-2.7967723	-2.2281779
H	1.8202058	-3.0497466	-1.0589722

FpPR• : radical iron complex
29
Energy = -2183.670358845

C	1.9501740	1.9984535	-0.6514647
C	3.2689752	2.0349931	-0.0792341
C	1.8749712	0.8530118	-1.4957695
H	1.1587565	2.7127756	-0.4746112
C	3.9918247	0.9136971	-0.5598644
H	3.6366379	2.7789764	0.6137800
C	3.1239204	0.1716406	-1.4321062
H	1.0135387	0.5489100	-2.0721754
H	5.0075326	0.6498767	-0.3006633
H	3.3783992	-0.7394056	-1.9536310
Fe	2.2852448	0.2681412	0.5050048
C	1.9067418	0.8863966	2.1042180
O	1.6784444	1.3167302	3.1562711

C	2.9816710	-1.2182911	1.1123440
O	3.5057757	-2.1724074	1.5166665
P	0.1527311	-0.6043896	0.5199951
C	0.0590019	-2.3661706	-0.1988795
C	0.2150777	-3.3864267	0.9459835
C	-1.3724236	-2.4737544	-0.7695784
C	1.0680771	-2.6671846	-1.3136955
H	1.2204076	-3.3632068	1.3743910
H	-0.5054708	-3.1921393	1.7479380
H	0.0324355	-4.4004340	0.5622187
H	-1.5206094	-1.7803224	-1.6048437
H	-1.5482971	-3.4951012	-1.1345683
H	-2.1233804	-2.2509139	-0.0033062
H	0.8907576	-3.6767887	-1.7099883
H	0.9687355	-1.9602158	-2.1441704
H	2.0973804	-2.6264521	-0.9473221

FpRP**A**$^{+}$.Fp^{-} : contact ion pair
68
Energy = -4407.879614309

C	-3.1312833	4.0466663	-1.4116583
C	-3.2277976	2.6184180	-1.5267347
C	-3.2841613	4.3670173	-0.0325552
H	-2.9902226	4.7491599	-2.2207444
C	-3.4533313	2.0636040	-0.2197882
H	-3.1368530	2.0551071	-2.4465957
C	-3.4807667	3.1446908	0.7019596
H	-3.2356978	5.3573041	0.4006194
H	-3.6111389	1.0221223	0.0186736
H	-3.6124214	3.0658993	1.7727977
Fe	-1.6267638	3.1072126	-0.2947049
C	-0.3289036	2.3954298	-1.1726828
O	0.5613396	1.9130796	-1.7964867
C	-0.5534496	3.8224043	0.8506434
O	0.1514775	4.3596741	1.6359593
C	4.5383467	-1.0797087	0.5603719
C	4.3282401	-0.2717004	1.7094263
C	3.6993600	-2.2406789	0.6758047
H	5.2039265	-0.8541442	-0.2605788
C	3.3587672	-0.9173765	2.5415856
H	4.8040384	0.6780230	1.9098292
C	2.9893753	-2.1382747	1.9019523
H	3.6253501	-3.0454725	-0.0414133
H	2.9971678	-0.5575184	3.4932856
H	2.2947168	-2.8688278	2.2878543
Fe	2.5251076	-0.4882415	0.6450670
C	2.3214125	1.2683069	0.7604066
O	2.3617481	2.4151447	0.8325289
C	2.5151316	-0.4368542	-1.1294297
O	2.7182914	-0.3884716	-2.2614616
C	0.7152332	-2.6429942	-3.6265869
C	0.0039706	-1.6632001	-2.9228181
C	-0.1229898	-1.7980000	-1.5472894
C	0.4332706	-2.9060081	-0.8762309
C	1.1516552	-3.8691727	-1.5716230
C	1.2901644	-3.7271826	-2.9593000
C	-0.8917884	-0.9284512	-0.5660230
C	0.0533080	-2.8714304	0.5986369
C	-1.4611217	-2.9287855	0.6439204
C	-2.0030841	-1.8338768	-0.0540816
C	-3.3760622	-1.7249095	-0.2309656
H	-3.8050110	-0.9073940	-0.8016542
C	-4.2075131	-2.6924074	0.3497370
C	-3.6686673	-3.7514008	1.0842854
C	-2.2804758	-3.8845608	1.2286205
H	-1.1796933	0.0707907	-0.8861629
H	0.8255639	-2.5545240	-4.7034346
H	-0.4201860	-0.8071505	-3.4386564
H	1.5843376	-4.7239725	-1.0588829
H	1.8427146	-4.4745915	-3.5209242
H	0.5736572	-3.5829940	1.2352559
H	-5.2843835	-2.6140685	0.2320970
H	-4.3288216	-4.4861172	1.5355834
H	-1.8559361	-4.7195077	1.7796136
P	0.3390723	-1.0156933	0.9021278
C	-0.4398304	-0.4894619	2.5521200
C	0.1592146	0.8673743	2.9600006
C	-1.9667723	-0.3012542	2.4942940
C	-0.0858594	-1.5673586	3.5935559
H	-0.0869547	1.6381933	2.2226879
H	1.2426311	0.8222361	3.0888798
H	-0.2848532	1.1578141	3.9193504
H	-2.5025980	-1.2392425	2.3509239
H	-2.2777676	0.1266361	3.4552850
H	-2.2369734	0.4083046	1.7068134
H	-0.4937486	-1.2575719	4.5623850
H	-0.5251623	-2.5358269	3.3363062
H	0.9936937	-1.6886319	3.7171246

Fp(tBuP**A**)$^{+}$: cation complex
53
Energy = -2723.419387215

C	2.1940279	2.1756978	-0.5961312
C	3.4601096	1.7813291	-0.0461506
C	1.8470256	1.2309307	-1.6003516
H	1.6067847	3.0328681	-0.2995851
C	3.8758410	0.5950600	-0.7101436
H	3.9989100	2.2903148	0.7405521
C	2.8701398	0.2395669	-1.6677694
H	0.9578469	1.2587036	-2.2109097
H	4.7846974	0.0440760	-0.5113821
H	2.8973737	-0.6064870	-2.3383992
Fe	2.0473888	0.2621603	0.2748267
C	1.6987963	0.8652777	1.8997981
O	1.5622300	1.2500370	2.9770670
C	2.7626991	-1.2138648	0.9297653
O	3.3380073	-2.1082138	1.3779271
C	-1.0416610	3.9258698	1.3687111
C	-1.1056952	2.6309272	1.9020063
C	-1.1621549	1.5546845	1.0264037

C	-1.1596553	1.7636060	-0.3665606
C	-1.1064470	3.0457151	-0.8971056
C	-1.0435106	4.1317234	-0.0124831
C	-1.2713589	0.0630402	1.3368017
C	-1.2727645	0.4305397	-1.0907680
C	-2.5954716	-0.1722327	-0.6467819
C	-2.5880377	-0.4010344	0.7416237
C	-3.6845654	-0.9699521	1.3756711
H	-3.6768178	-1.1582619	2.4453052
C	-4.8040262	-1.3016304	0.5995822
C	-4.8181092	-1.0563603	-0.7757817
C	-3.7087045	-0.4860602	-1.4139839
H	-1.0738343	-0.2266094	2.3670799
H	-0.9949645	4.7785107	2.0393498
H	-1.1061387	2.4774843	2.9767056
H	-1.1129997	3.2078919	-1.9713293
H	-0.9981311	5.1425051	-0.4066686
H	-1.0942740	0.4422981	-2.1643740
H	-5.6705253	-1.7519723	1.0744156
H	-5.6962205	-1.3166897	-1.3591025
H	-3.7161996	-0.3133982	-2.4863341
P	-0.0581264	-0.5531873	0.0116495
C	-0.1846541	-2.4163714	-0.2709841
C	-0.0278691	-3.0702920	1.1157872
C	-1.4948351	-2.9142947	-0.9107958
C	0.9564436	-2.8222091	-1.2257894
H	0.8505582	-2.7167092	1.6613972
H	-0.9135542	-2.8852595	1.7311171
H	0.0772962	-4.1529743	0.9843058
H	-1.6734062	-2.4528968	-1.8847608
H	-1.3803292	-3.9938645	-1.0659234
H	-2.3652568	-2.7519930	-0.2751602
H	0.8984023	-3.9045376	-1.3843879
H	0.8464489	-2.3346797	-2.2004334
H	1.9481288	-2.5937027	-0.8353965

Fp(THF)$^{\bullet}$: radical complex
28
Energy = -1916.922887669

Fe	-0.5806331	-0.4218805	0.1344371
C	0.6780078	-0.1198919	1.8276140
C	-1.7847949	-0.1954434	-1.1241424
C	-1.1617831	1.2044256	1.3885370
C	-0.9194818	-2.1351244	0.3244572
C	-0.0784724	1.6123205	0.5221312
C	1.0610755	0.8278022	0.8418233
C	-0.6968115	0.1402492	2.1910305
H	1.3143108	-0.8737310	2.2710373
H	-2.1556001	1.6302353	1.3993058
H	-0.1205937	2.4113346	-0.2054574
H	2.0254974	0.8840690	0.3567894
H	-1.2672707	-0.4026691	2.9318453
O	-2.6332886	-0.0210177	-1.9055352
O	-1.1894790	-3.2549626	0.5073144
O	1.1258888	-0.9942230	-1.6520695
C	2.3589712	-1.6719477	-1.2728760
C	1.4177157	0.0296550	-2.6411416
C	3.4909895	-0.8859562	-1.9336398
C	2.8016403	-0.3138446	-3.1825510
H	2.3049461	-2.7019522	-1.6453554
H	2.4135266	-1.6903642	-0.1810129
H	1.4089381	1.0120886	-2.1506207
H	0.6222537	-0.0012897	-3.3899918
H	3.8262860	-0.0719899	-1.2810507
H	4.3525530	-1.5171430	-2.1661227
H	3.3153512	0.5581233	-3.5962019
H	2.7273541	-1.0792987	-3.9624909

Fp(THF)$^{+}$: cation complex
28
Energy = -1916.775180316

Fe	-0.2786386	-0.3886532	-0.0082944
C	0.6911924	-0.0724807	1.8385671
C	-1.5770011	-0.1988637	-1.2157864
C	-1.2257241	1.0405596	1.1673009
C	-0.6057057	-2.1223292	0.2558334
C	-0.1020844	1.6470744	0.5048982
C	1.0653508	0.9615199	0.9402110
C	-0.7400075	-0.0180594	1.9838716
H	1.3573836	-0.7630027	2.3358769
H	-2.2620836	1.3275050	1.0560584
H	-0.1389821	2.4823060	-0.1796919
H	2.0697994	1.1451286	0.5821467
H	-1.3440726	-0.6699201	2.5994342
O	-2.4558036	-0.0555832	-1.9432219
O	-0.8563374	-3.2217618	0.4800077
O	1.1166274	-0.7383898	-1.3888085
C	2.2903642	-1.5995960	-1.1114571
C	1.4354402	0.2367537	-2.4453707
C	3.3886319	-1.0574719	-2.0169388
C	2.6072637	-0.4000089	-3.1687425
H	1.9849093	-2.6180432	-1.3629642
H	2.5144959	-1.5276384	-0.0464481
H	1.6997580	1.1891730	-1.9748678
H	0.5309166	0.3519596	-3.0435525
H	3.9859466	-0.3088861	-1.4876423
H	4.0528900	-1.8535743	-2.3588733
H	3.2001861	0.3424931	-3.7068381
H	2.2523815	-1.1526371	-3.8786449

Fp- : aa
15
Energy = -1684.435537939

C	1.8224156	-1.3903906	0.8963956
C	2.4823386	-1.2274800	-0.3599897
C	2.2908138	-0.3391822	1.7508966
H	1.1338543	-2.1797345	1.1654194
C	3.3613831	-0.0898426	-0.2768890
H	2.3337088	-1.8419101	-1.2385065
C	3.2454332	0.4595701	1.0279630

H	1.9666128	-0.1609516	2.7680643
H	3.9898754	0.2834302	-1.0746629
H	3.7800097	1.3173745	1.4136600
Fe	1.3652301	0.4859612	0.0781852
C	0.6512785	0.8238663	-1.4515616
O	0.1600327	1.0544979	-2.5088102
C	0.2825663	1.5787396	0.8492493
O	-0.4635781	2.3319367	1.3865514

$Fp^{\bullet}$: radical
15
Energy = -1684.323705792

C	1.9275837	-1.5080425	0.9228535
C	2.4895981	-1.2550107	-0.3592479
C	2.2549747	-0.4134828	1.7705796
H	1.2973887	-2.3477066	1.1853433
C	3.2257577	-0.0132750	-0.2885728
H	2.4076126	-1.8925615	-1.2287113
C	3.0818518	0.5034881	1.0197333
H	1.9626556	-0.2986634	2.8052099
H	3.7724311	0.4433172	-1.1018242
H	3.4978629	1.4287811	1.3930779
Fe	1.1710913	0.3223248	0.1163265
C	0.6528878	0.7550042	-1.5081491
O	0.3651868	1.0652732	-2.5921225
C	0.3817020	1.6912586	0.8895544
O	-0.0866098	2.6251801	1.4019139

Fp^{+} : cation
15
Energy = -1684.122856964

Fe	-0.2996339	-0.3830606	0.0290932
C	0.6846154	-0.0626163	1.8526422
C	-1.5709801	-0.1694326	-1.2300518
C	-1.2428324	1.0399845	1.1769290
C	-0.6149691	-2.1352334	0.3071228
C	-0.1256938	1.6497168	0.5100262
C	1.0462426	0.9682862	0.9422192
C	-0.7441336	-0.0159209	2.0032740
H	1.3586333	-0.7518658	2.3409444
H	-2.2836647	1.3097466	1.0631229
H	-0.1672874	2.4737007	-0.1878667
H	2.0431777	1.1324296	0.5506317
H	-1.3470047	-0.6725003	2.6153882
O	-2.4240726	0.0069857	-1.9734900
O	-0.8506646	-3.2278958	0.5564461

Styrene : CH_2=CHPh
16
Energy = -309.8395674888

C	2.3189204	1.5285649	0.0001575
C	1.4736370	0.4874509	-0.0002855
H	1.9771779	2.5604165	0.0006781
H	3.3932274	1.3725332	-0.0002396
H	1.8923297	-0.5191796	-0.0004213
C	0.0054825	0.5375944	-0.0001086
C	-0.7130888	-0.6709357	-0.0001181
C	-0.7229278	1.7422079	-0.0002397
C	-2.1077241	-0.6816036	-0.0002527
H	-0.1645425	-1.6101300	-0.0005473
C	-2.1141347	1.7329703	0.0000450
H	-0.1972193	2.6928023	-0.0003957
C	-2.8147798	0.5214332	-0.0000273
H	-2.6409325	-1.6282341	-0.0008016
H	-2.6578930	2.6737068	0.0007089
H	-3.9010033	0.5188131	0.0003304

*t*BuP**A**$^{\bullet-}$: radical anion
38
Energy = -1039.251275987

C	-5.5239127	-1.1925934	-0.1200757
C	-4.4570174	-0.9540142	0.7579214
C	-3.7544236	0.2462727	0.7172938
C	-4.0887102	1.2546674	-0.2393033
C	-5.1907791	1.0074727	-1.0961611
C	-5.8921304	-0.1931358	-1.0333451
C	-2.7249017	0.6658837	1.7292085
C	-3.2732228	2.4267052	-0.2975447
C	-1.9104915	2.3612185	0.1374341
C	-1.5605082	1.3503844	1.0828379
C	-0.2302369	1.1294456	1.4169367
H	0.0181479	0.3347761	2.1188354
C	0.7879052	1.9552051	0.9099102
C	0.4470997	3.0006682	0.0445913
C	-0.8776348	3.1926113	-0.3507576
H	-2.4086542	-0.1728281	2.3604781
H	-6.0730211	-2.1295598	-0.0764429
H	-4.1855939	-1.7018251	1.5019394
H	-5.4743525	1.7653946	-1.8249036
H	-6.7293026	-0.3606074	-1.7083619
H	-3.5623873	3.2257700	-0.9797874
H	1.8213839	1.7976610	1.2072926
H	1.2224474	3.6606118	-0.3397291
H	-1.1230880	3.9819769	-1.0597621
P	-3.7794833	1.8718071	2.8173768
C	-2.5786041	2.5178781	4.1419916
C	-1.7338851	1.3935065	4.7601999
C	-1.6604630	3.6181132	3.5745457
C	-3.4709304	3.1353807	5.2353954
H	-2.3718927	0.5917449	5.1502962
H	-1.0493428	0.9622657	4.0245649
H	-1.1291024	1.7858932	5.5922154
H	-2.2557118	4.4328722	3.1477410
H	-1.0328264	4.0350035	4.3784325
H	-1.0043246	3.2329820	2.7903823
H	-2.8490366	3.5945825	6.0182045
H	-4.1216160	3.9149799	4.8200858
H	-4.1082544	2.3749301	5.7011635

*t*BuP**A** : dibenzo-7-phosphanorbornadiene

38

Energy = -1039.188170159

C	-5.1036378	-1.2345930	-0.5894752
C	-4.1259439	-1.2066327	0.4152919
C	-3.6721211	0.0255703	0.8697232
C	-4.1863250	1.2236524	0.3326420
C	-5.1562361	1.1959830	-0.6620933
C	-5.6126143	-0.0476152	-1.1217468
C	-2.6344989	0.3433148	1.9397951
C	-3.5179764	2.4169009	1.0057954
C	-2.0342824	2.3038962	0.7034844
C	-1.5218612	1.1136183	1.2546106
C	-0.1769469	0.7956269	1.1280191
H	0.2225611	-0.1198395	1.5568919
C	0.6682533	1.6929420	0.4535290
C	0.1580863	2.8661932	-0.1019387
C	-1.2077476	3.1749588	0.0094140
H	-2.3291275	-0.5015548	2.5556657
H	-5.4703585	-2.1895723	-0.9556996
H	-3.7358909	-2.1319960	0.8311519
H	-5.5580231	2.1178367	-1.0745888
H	-6.3717569	-0.0877220	-1.8980804
H	-3.9786251	3.3836911	0.8061506
H	1.7282376	1.4705127	0.3662259
H	0.8234402	3.5518222	-0.6195375
H	-1.5993650	4.0951835	-0.4165616
P	-3.6722917	1.7124152	2.7852211
C	-2.5625428	2.6691346	3.9832337
C	-2.0163851	1.6052303	4.9554839
C	-1.4078389	3.5143784	3.4298261
C	-3.5385122	3.5987233	4.7371821
H	-2.8151891	0.9557578	5.3347044
H	-1.2622693	0.9775362	4.4691133
H	-1.5478303	2.0995187	5.8160317
H	-1.7638568	4.2635054	2.7164927
H	-0.9350621	4.0453274	4.2676767
H	-0.6454178	2.9080458	2.9367087
H	-2.9963335	4.1351260	5.5265786
H	-3.9783579	4.3439909	4.0639252
H	-4.3542109	3.0332337	5.2002533

THF : solvent

13

Energy = -232.5925824574

O	-0.0007626	-0.0000911	1.2593978
C	-0.0971361	1.1799521	0.4199399
C	0.2594463	0.7229594	-0.9942347
C	-0.2595389	-0.7230429	-0.9942589
C	0.0971168	-1.1799069	0.4198871
H	0.5841228	1.9377328	0.8197405
H	-1.1254179	1.5650418	0.4678329
H	1.3455406	0.7368645	-1.1385325
H	-0.2017489	1.3481470	-1.7636608
H	-1.3456518	-0.7370286	-1.1384432
H	0.2016788	-1.3482419	-1.7636601
H	-0.5834449	-1.9383888	0.8195178
H	1.1257959	-1.5639980	0.4679240

TO^- : TEMPO$^-$ anion

29

Energy = -484.0919496836

N	-0.7673654	-0.4170811	0.0000015
O	-2.0739008	0.1474414	0.0000912
C	-0.0697671	-0.0349501	1.2702967
C	1.3711206	-0.5734314	1.2424659
C	2.1393393	-0.1261423	-0.0002462
C	1.3710846	-0.5740996	-1.2428096
C	-0.0696390	-0.0346367	-1.2703080
H	1.8864763	-0.2617437	2.1617011
H	2.2573122	0.9655428	-0.0005317
H	3.1522037	-0.5509261	-0.0000560
H	1.8864603	-0.2626677	-2.1621175
H	1.3248287	-1.6729414	-1.2497011
H	1.3250531	-1.6721507	1.2496979
C	-0.1071207	1.4796968	1.5682133
H	-1.0845673	1.8362987	1.2208481
H	-0.0081900	1.6710371	2.6443336
H	0.6854840	2.0303393	1.0528001
C	-0.8400350	-0.7586803	2.3845507
H	-0.8439097	-1.8359113	2.1822826
H	-0.3753514	-0.5774472	3.3626552
H	-1.8735122	-0.4021480	2.3913767
C	-0.1061259	1.4799225	-1.5686689
H	-0.0070651	1.6705528	-2.6449143
H	-1.0835059	1.8369265	-1.2216162
H	0.6865959	2.0305650	-1.0534911
C	-0.8415124	-0.7570835	-2.3839274
H	-0.3770722	-0.5774332	-3.3624417
H	-0.8473324	-1.8335578	-2.1803113
H	-1.8744251	-0.3989370	-2.3901733

$TO^{\bullet}$: TEMPO radical

29

Energy = -484.0199030012

N	-0.7627351	-0.1471379	-0.0000154
O	-2.0366347	0.0541374	-0.0000326
C	-0.0714612	0.0005819	1.3291460
C	1.3615650	-0.5501892	1.2412255
C	2.1127348	-0.0744844	-0.0002121
C	1.3611732	-0.5511982	-1.2410188
C	-0.0714458	0.0004682	-1.3291372
H	1.8853716	-0.2606483	2.1594146
H	2.2106492	1.0180042	-0.0007216
H	3.1296562	-0.4819469	-0.0001521
H	1.8850476	-0.2631161	-2.1596222
H	1.3149451	-1.6481839	-1.2258695
H	1.3161386	-1.6472188	1.2273162
C	-0.0806409	1.4874248	1.7338071
H	-1.0985256	1.8809443	1.6633598
H	0.2640150	1.5827755	2.7687891

H	0.5746158	2.0886985	1.0982503
C	-0.8736297	-0.8129524	2.3523774
H	-0.9365153	-1.8611917	2.0432141
H	-0.3654048	-0.7634177	3.3208837
H	-1.8847469	-0.4154572	2.4601253
C	-0.0796632	1.4873995	-1.7336102
H	0.2649062	1.5826739	-2.7686244
H	-1.0972823	1.8815795	-1.6630035
H	0.5760595	2.0882210	-1.0981104
C	-0.8741211	-0.8122244	-2.3526244
H	-0.3652229	-0.7636071	-3.3208227
H	-0.9386898	-1.8602923	-2.0432336
H	-1.8845976	-0.4132879	-2.4610984

TS1 : FpCl-induced **A** release from RP**A**
54
Energy = -3183.815223750

Fe	-1.9403834	0.2148502	0.1825278
C	-3.7128705	0.3102773	-0.9550998
C	-2.7008082	-1.0380502	1.1597038
C	-1.6186519	0.6611107	-1.8600201
C	-1.5660726	1.1990602	1.5976695
C	-1.9955233	1.8385007	-1.1580283
C	-3.3012214	1.6205082	-0.6003635
C	-2.6657244	-0.2969322	-1.7275749
H	-4.6431744	-0.1613670	-0.6697433
H	-0.6775520	0.5049046	-2.3635923
H	-1.3953656	2.7304340	-1.0537436
H	-3.8629564	2.3224547	-0.0002434
H	-2.6869594	-1.2903080	-2.1512202
O	-3.3178296	-1.7883761	1.7861061
O	-1.4218851	1.8602557	2.5340663
C	0.2782210	-2.5473066	-0.3462991
P	0.1541741	-0.7401333	0.3436134
C	1.4975467	-3.2936880	0.2177200
C	0.3838433	-2.4629298	-1.8766941
C	3.8065509	-1.1471948	-1.5428475
H	3.7781939	-1.2466738	-2.6255078
C	4.7980230	-1.7863116	-0.8057752
H	5.5450978	-2.3873870	-1.3175753
C	4.8440156	-1.6646895	0.5935202
H	5.6201081	-2.1750534	1.1566434
C	3.8875196	-0.9000431	1.2583051
H	3.8997475	-0.8201600	2.3423764
C	2.8969672	-0.2375386	0.5264171
C	2.8412455	-0.3550390	-0.8895512
C	1.0998100	2.7775378	-1.7823748
H	1.0755256	2.7012042	-2.8671852
C	0.8307666	3.9905051	-1.1587993
H	0.5951587	4.8646436	-1.7605839
C	0.8615225	4.0988251	0.2430331
H	0.6457318	5.0512994	0.7184276
C	1.1701849	2.9857758	1.0202865
H	1.1866211	3.0596871	2.1043570
C	1.4700338	1.7643495	0.4040290
C	1.4323612	1.6451914	-1.0109370
C	1.7518877	0.3410156	-1.5343672
H	1.6183243	0.1799312	-2.6036809
C	1.7723378	0.5106275	1.0944057
H	1.6951230	0.5223028	2.1783315
C	-0.9914508	-3.3384417	0.0050919
H	-1.1530206	-3.3915096	1.0829949
H	-0.8659542	-4.3611219	-0.3739603
H	-1.8784993	-2.9111202	-0.4701038
H	2.4378907	-2.8117681	-0.0499120
H	1.4973970	-4.3059627	-0.2082369
H	1.4405441	-3.3802372	1.3050080
H	1.3593928	-2.0976154	-2.1956720
H	-0.3893224	-1.8212302	-2.3085634
H	0.2448351	-3.4721003	-2.2839646
Cl	0.0050609	-1.4596052	2.4610238

TS2 : FLP-like styrene addition to **2**-Cl
46
Energy = -2953.802429542

C	-2.8879999	-0.3834879	-0.6506187
C	-2.8468672	0.8513623	-1.3636807
C	-1.7322586	-1.1383487	-1.0280608
H	-3.6480701	-0.6867580	0.0551668
C	-1.6706649	0.8673486	-2.1783576
H	-3.5739812	1.6484003	-1.2886307
C	-0.9895248	-0.3679752	-1.9673282
H	-1.4527058	-2.1124012	-0.6514430
H	-1.3493509	1.6705112	-2.8244208
H	-0.0531747	-0.6594518	-2.4189855
Fe	-1.1533163	0.6917058	-0.1329704
C	-0.9169249	-0.0688354	1.4582040
O	-1.0545518	-0.4826049	2.5338285
C	-1.6703359	2.1846674	0.6202858
O	-2.1470267	3.1120425	1.1296897
P	1.0824956	1.1596273	-0.3481963
C	1.7101730	2.6454798	0.6610414
C	3.2344268	2.7923479	0.4684901
C	1.0621349	3.9652693	0.2136803
C	1.3971633	2.3725133	2.1443768
H	3.5661650	3.6760168	1.0272591
H	3.7986168	1.9345962	0.8430584
H	3.4843777	2.9404420	-0.5855523
H	1.4194371	4.7673175	0.8721968
H	1.3478212	4.2104088	-0.8120401
H	-0.0257909	3.9437191	0.2723402
H	1.8354040	1.4319280	2.4922629
H	1.8219935	3.1840145	2.7482310
H	0.3214282	2.3423182	2.3359689
Cl	1.5487752	1.9665076	-2.3402648
C	2.3582229	-0.4102745	0.3970676
C	1.5939320	-1.3771596	1.0858639
H	2.8451382	-0.6915038	-0.5353648
H	2.9996380	0.2100601	1.0178361
H	1.4151418	-1.2313190	2.1461085

C	0.9869843	-2.5140844	0.4590398
C	0.1489974	-3.3888147	1.2008120
C	1.1596083	-2.8120149	-0.9190176
C	-0.4933565	-4.4639391	0.6002642
H	-0.0040396	-3.1908142	2.2589974
C	0.5174457	-3.8934611	-1.5131807
H	1.7995478	-2.1810828	-1.5294101
C	-0.3229597	-4.7269130	-0.7666296
H	-1.1385734	-5.1035391	1.1977961
H	0.6670240	-4.0852779	-2.5730466
H	-0.8301407	-5.5646640	-1.2358683

TS3 : PC_2 ring-contraction of $FePC_3$-ring **3a**
46
Energy = -2953.805162984

C	-0.7131010	3.1348779	0.1126390
C	0.6595130	3.3860859	-0.1800630
C	-1.1933655	2.1865842	-0.8478655
H	-1.2855340	3.5768522	0.9156558
C	1.0390653	2.5912429	-1.3065318
H	1.3106455	4.0489645	0.3733223
C	-0.1119951	1.8529940	-1.7131734
H	-2.1901120	1.7714145	-0.8870916
H	2.0201616	2.5297415	-1.7528413
H	-0.1439452	1.1516788	-2.5344456
Fe	0.3794524	1.3549101	0.2807786
C	-0.7064065	0.5394454	1.4223086
O	-1.4162712	0.1664666	2.2525542
C	1.6370702	1.5572222	1.4934757
O	2.4159902	1.8008266	2.3138159
P	1.0857085	-0.6392256	-0.4799469
C	2.0780461	-1.7685303	0.6801650
C	2.1950006	-3.1704182	0.0574213
C	3.5025712	-1.1979173	0.8443903
C	1.4312709	-1.8495662	2.0743239
H	2.8567049	-3.7708228	0.6929156
H	1.2323662	-3.6850928	-0.0012129
H	2.6378162	-3.1201612	-0.9419698
H	4.0082610	-1.7882837	1.6177676
H	4.0680009	-1.2675970	-0.0849059
H	3.5023696	-0.1552240	1.1674068
H	0.4238888	-2.2660139	2.0391019
H	2.0580263	-2.4899792	2.7078199
H	1.3811176	-0.8669210	2.5519454
Cl	2.8026460	-0.2306305	-2.1190609
C	-0.0274849	-1.6912026	-1.4765615
C	-0.9263736	-1.9817817	-0.3418055
H	-0.4617801	-1.1232700	-2.3000699
H	0.5416721	-2.5308331	-1.8837901
H	-0.6420200	-2.8009154	0.3095995
C	-2.2852234	-1.5352502	-0.2539871
C	-3.1041100	-1.9656248	0.8243319
C	-2.8967820	-0.6864504	-1.2124335
C	-4.4254715	-1.5597426	0.9448598
H	-2.6722526	-2.6253799	1.5736634
C	-4.2228179	-0.2814469	-1.0857939
H	-2.3288235	-0.3519428	-2.0763250
C	-5.0020195	-0.7047682	-0.0046978
H	-5.0169190	-1.9099740	1.7876571
H	-4.6555484	0.3669140	-1.8445984
H	-6.0353801	-0.3850065	0.0921641

TS4a : ionic Fp_2-induced **A** release from RP**A**
68
Energy = -4407.859220093

C	-3.9216192	1.3834850	-1.8356879
C	-3.4473838	0.0262604	-1.8150752
C	-4.3112373	1.7206204	-0.5178144
H	-3.9548503	2.0321277	-2.6998548
C	-3.5627051	-0.4695808	-0.4868195
H	-3.0885634	-0.5249833	-2.6737864
C	-4.0744249	0.5804078	0.3265882
H	-4.6907437	2.6792927	-0.1918565
H	-3.2987532	-1.4631010	-0.1597266
H	-4.2862494	0.5294563	1.3852239
Fe	-2.2584793	1.2227764	-0.5430193
C	-1.0683840	1.5655884	-1.7670890
O	-0.3780861	1.8110105	-2.6752743
C	-1.9252236	2.5660686	0.5165645
O	-1.8623795	3.5214879	1.1838064
C	3.7491146	2.1460193	0.7707399
C	2.8526058	2.6085574	1.7766782
C	3.8302833	0.7210972	0.8911811
H	4.2625290	2.7579809	0.0427209
C	2.3681812	1.4822401	2.5070842
H	2.5688973	3.6385198	1.9445067
C	2.9905933	0.3205424	1.9602573
H	4.4147649	0.0631306	0.2639295
H	1.6943454	1.5203876	3.3488674
H	2.8512575	-0.6916972	2.2967676
Fe	1.8341088	1.3122050	0.4519041
C	0.9582149	2.7612713	-0.0078352
O	0.6113003	3.8131964	-0.3463881
C	2.1739894	0.9783516	-1.2536650
O	2.5790507	0.9937991	-2.3354669
C	2.4314103	-1.8514517	-3.4539114
C	1.1241531	-1.6326658	-3.0135545
C	0.8504969	-1.6990767	-1.6484693
C	1.8686481	-2.0021026	-0.7164720
C	3.1804072	-2.1878276	-1.1643963
C	3.4538681	-2.1158690	-2.5326381
C	-0.4668403	-1.5419665	-0.9712895
C	1.3391582	-2.1146134	0.6527547
C	0.2424032	-3.1012185	0.6939612
C	-0.7772902	-2.8011057	-0.2362035
C	-1.8942440	-3.6247799	-0.3603842
H	-2.6654435	-3.4064975	-1.0954310
C	-2.0168460	-4.7406832	0.4741822
C	-1.0228244	-5.0274198	1.4185325
C	0.1108452	-4.2168904	1.5271445

H	-1.2591776	-1.1455196	-1.5951621
H	2.6604753	-1.8008348	-4.5147355
H	0.3336700	-1.4032711	-3.7237011
H	3.9743509	-2.4199655	-0.4577775
H	4.4690695	-2.2740710	-2.8873121
H	2.0804584	-2.2154465	1.4418239
H	-2.8867110	-5.3861678	0.3899468
H	-1.1289679	-5.8939629	2.0658610
H	0.8859529	-4.4553683	2.2519950
P	-0.0211856	-0.2636874	0.5496381
C	-0.7721352	-0.5240652	2.3434895
C	-1.1721812	0.8534922	2.8956920
C	-2.0178210	-1.4204108	2.3879430
C	0.2572910	-1.1421953	3.3068660
H	-2.1246805	1.1764868	2.4764788
H	-0.4268455	1.6238286	2.6821164
H	-1.2870437	0.7736533	3.9844794
H	-1.8067726	-2.4546963	2.1159056
H	-2.3942411	-1.4088158	3.4203622
H	-2.8050388	-1.0323115	1.7476029
H	-0.2134022	-1.1947462	4.2970329
H	0.5338870	-2.1556362	3.0204912
H	1.1573315	-0.5397022	3.4106632

TS4• : radical Fp•-induced **A** release from RP**A**
53
Energy = -2723.528740815

C	2.4531381	1.6879294	-0.6591710
C	3.6504560	1.1335304	-0.0835908
C	2.1100650	0.9039350	-1.7906910
H	1.9160201	2.5563614	-0.3040561
C	4.0050297	-0.0116587	-0.8375245
H	4.1728108	1.5117776	0.7841001
C	3.0281668	-0.1733351	-1.8860142
H	1.2377646	1.0488033	-2.4105945
H	4.8487350	-0.6618627	-0.6521014
H	3.0196641	-0.9582956	-2.6291217
Fe	2.1003718	-0.3033793	0.0407712
C	1.7361988	0.0426278	1.7128422
O	1.5803862	0.2533788	2.8450149
C	2.5112076	-1.9524593	0.4444222
O	2.9176273	-2.9964929	0.7590237
C	0.0451061	3.9174576	0.9583161
C	-0.2877138	2.6546603	1.4661151
C	-0.8369861	1.6986242	0.6181953
C	-1.0937998	2.0035789	-0.7420281
C	-0.7256353	3.2562680	-1.2487985
C	-0.1599261	4.2079451	-0.3957125
C	-1.1882431	0.2647289	0.9397564
C	-1.7593035	0.9208450	-1.4748739
C	-2.9039713	0.3556034	-0.7580113
C	-2.6345780	0.0466926	0.5974430
C	-3.6246476	-0.4756685	1.4201414
H	-3.4041498	-0.7175207	2.4572536
C	-4.8980751	-0.7273442	0.8896828
C	-5.1627798	-0.4529868	-0.4566309
C	-4.1751325	0.0906253	-1.2823692
H	-0.9182663	-0.0484173	1.9486297
H	0.4777092	4.6672482	1.6147763
H	-0.1006038	2.4191240	2.5098046
H	-0.9009848	3.4936063	-2.2954026
H	0.1164506	5.1837272	-0.7862955
H	-1.8032332	0.9806251	-2.5603796
H	-5.6744821	-1.1499677	1.5211670
H	-6.1484377	-0.6614533	-0.8646133
H	-4.3905923	0.3060374	-2.3261415
P	-0.1869211	-0.6394793	-0.4603543
C	-0.7388339	-2.4631219	-0.4462229
C	-0.6721905	-3.0566093	0.9694641
C	-2.1653270	-2.6519763	-1.0033964
C	0.1994968	-3.2032833	-1.4198773
H	0.3253684	-2.9737398	1.4074486
H	-1.3840568	-2.5550175	1.6329123
H	-0.9394114	-4.1218622	0.9336113
H	-2.2816787	-2.1665877	-1.9766620
H	-2.3305424	-3.7295895	-1.1388373
H	-2.9355199	-2.2726182	-0.3318015
H	-0.0849961	-4.2628340	-1.4525138
H	0.0993340	-2.7949865	-2.4320486
H	1.2496097	-3.1426431	-1.1348823

TS5 : FLP-like styrene addition to **4** Fp_2PR
60
Energy = -4177.890319237

C	2.7828902	0.2286792	-3.4671951
C	2.2851701	1.5317088	-3.2225738
C	2.9967654	-0.4204001	-2.1983380
H	2.9587310	-0.2118943	-4.4387770
C	2.1865240	1.7004157	-1.8016173
H	2.0106959	2.2613925	-3.9710411
C	2.6497106	0.5059056	-1.1833331
H	3.3737268	-1.4224555	-2.0521141
H	1.8670199	2.5995448	-1.2967124
H	2.6979987	0.3177772	-0.1220497
Fe	0.9396134	0.0932777	-2.4364710
C	0.5438822	-1.1793887	-3.5649292
O	0.3931154	-1.9358344	-4.4358733
C	-0.5588142	0.9490470	-2.7175308
O	-1.5267852	1.5387985	-2.9762230
P	-0.1263808	-0.8103241	-0.4821389
C	-1.2817117	-2.2478932	-1.0612162
C	-0.4046174	-3.4299030	-1.5423321
C	-2.1335013	-2.7652967	0.1113964
C	-2.2398655	-1.8516797	-2.1977634
H	0.4682579	-3.1092123	-2.1145010
H	-0.0543298	-4.0353263	-0.7018352
H	-1.0092544	-4.0830599	-2.1836689
H	-2.9065875	-2.0499923	0.4021165
H	-2.6395042	-3.6880994	-0.2029788
H	-1.5223681	-2.9976861	0.9878818

H	-2.9178224	-2.6951986	-2.3907611
H	-2.8498123	-0.9857776	-1.9454812
H	-1.7171668	-1.6360391	-3.1292140
C	-0.5009468	2.5544351	0.1684395
C	-1.7155539	2.8205119	0.8775123
C	0.4009762	1.9305076	1.0796477
H	-0.3132346	2.8037821	-0.8633112
C	-1.5676905	2.3559617	2.2127106
H	-2.6022963	3.2770385	0.4595498
C	-0.2511401	1.8023751	2.3349764
H	1.4035518	1.5961259	0.8745503
H	-2.3183036	2.3954523	2.9893316
H	0.1722064	1.3484686	3.2201914
Fe	-1.3452404	0.7473572	0.8594969
C	-2.8378565	0.5641719	-0.0277984
O	-3.8921752	0.5607883	-0.5164876
C	-1.7674881	-0.4637762	2.0709626
O	-2.1873934	-1.0900610	2.9529410
C	1.2381284	-2.0367453	0.9407919
C	0.8994054	-1.9125960	2.2907966
H	2.1721362	-1.6077187	0.5891069
H	0.9996703	-2.9731790	0.4502311
H	0.0999477	-2.5397350	2.6774431
C	1.4441045	-0.9495946	3.2037543
C	0.8937251	-0.8068483	4.5062264
C	2.5297219	-0.0921594	2.8805555
C	1.3653624	0.1481072	5.3986285
H	0.0646453	-1.4496669	4.7925024
C	2.9970512	0.8631020	3.7759028
H	3.0205949	-0.1912917	1.9162025
C	2.4167438	1.0044065	5.0423075
H	0.9062407	0.2347447	6.3806610
H	3.8254355	1.5053442	3.4859610
H	2.7801802	1.7553085	5.7374509

TS6$^•$: PC_2 ring-contraction of radical **3co$^•$**
45
Energy = -2493.525515259

P	-1.0002538	0.3044234	0.0950034
C	-2.6371503	0.4973545	1.0585704
C	-3.8432378	0.3362888	0.1165583
C	-2.7595314	-0.5237893	2.1991259
C	-2.6554813	1.9354844	1.6063485
H	-3.8132396	1.0632190	-0.7012680
H	-3.9091616	-0.6710987	-0.3069723
H	-4.7619173	0.5116318	0.6917459
H	-1.9691866	-0.3993875	2.9430990
H	-3.7248663	-0.3935494	2.7075444
H	-2.7156588	-1.5507998	1.8214287
H	-3.5888736	2.1071847	2.1581901
H	-1.8232054	2.1287734	2.2865862
H	-2.6029245	2.6605449	0.7870171
C	2.1501153	1.7566597	0.6926760
C	2.8442759	1.1027463	1.7748656
C	2.0041906	0.8165898	-0.3598920
H	1.8248000	2.7872772	0.6788614
C	3.0842595	-0.2397472	1.3980047
H	3.1153833	1.5570339	2.7178858
C	2.5354460	-0.4264131	0.0795834
H	1.5019803	0.9884960	-1.3009096
H	3.5685695	-0.9970566	1.9982505
H	2.5602497	-1.3462915	-0.4874685
Fe	0.9938189	0.1245262	1.4366935
C	0.3118411	0.8585847	2.8701426
O	-0.0209424	1.3328139	3.8805163
C	0.4854084	-1.4911421	1.8688115
O	0.1894383	-2.5774429	2.1594244
C	-1.2704222	-1.3419679	-0.7528168
C	-1.5676828	-0.5320220	-1.9575694
H	-2.1010748	-1.9299664	-0.3598383
H	-0.3669175	-1.9474471	-0.8074081
H	-2.5868411	-0.1857634	-2.0892245
C	-0.6525456	-0.2742097	-3.0340813
C	0.6360974	-0.8574586	-3.1072678
C	-1.0330993	0.5967814	-4.0868111
C	1.4941407	-0.5755081	-4.1649467
H	0.9582560	-1.5461667	-2.3323578
C	-0.1737295	0.8731094	-5.1410450
H	-2.0187632	1.0558015	-4.0548850
C	1.1017992	0.2929013	-5.1889967
H	2.4769319	-1.0393075	-4.1948170
H	-0.4936158	1.5457541	-5.9327931
H	1.7764676	0.5125890	-6.0112803

TS7c$^•$: PC_2 ring-closing of radical FpPRSty$^•$
45
Energy = -2493.508273826

Fe	0.9072088	-0.0176197	1.2376392
C	2.6274786	1.5849585	1.6461767
H	2.7419311	2.0103582	2.6346096
C	3.2425579	0.3926799	1.1732267
H	3.9036979	-0.2537678	1.7337074
C	2.8179222	0.1986798	-0.1851406
H	3.1185019	-0.6155630	-0.8310157
C	1.9796590	1.2953617	-0.5540579
H	1.4794795	1.4119074	-1.5049967
C	1.8393842	2.1403705	0.5769873
H	1.2460709	3.0427452	0.6320707
C	0.7432387	-1.7395671	1.1778250
O	0.6424394	-2.8987711	1.1535797
C	0.3569710	0.0429459	2.8820446
O	0.0860617	0.0515210	4.0144257
P	-1.1139677	0.3311672	0.1826492
C	-2.6309935	0.6107039	1.2894470
C	-3.8310662	0.8755093	0.3575260
H	-3.6200981	1.6953241	-0.3372802
H	-4.1017783	-0.0114851	-0.2244502
H	-4.7033515	1.1522837	0.9642294
H	-2.1442669	2.7318371	1.4375632
C	-2.3626181	1.8886643	2.1022598

H -1.5205349 1.7689118 2.7883124
H -3.2501357 2.1408486 2.6971034
C -2.9545629 -0.5674473 2.2180393
H -3.1386446 -1.4868428 1.6525009
H -2.1447852 -0.7592316 2.9257079
H -3.8627884 -0.3457068 2.7953225
C -1.6801552 -1.2158817 -0.7706889
H -0.9406668 -2.0157554 -0.7488982
H -2.6244973 -1.5944491 -0.3750047
C -1.8219884 -0.5634431 -2.0904837
H -2.7584156 -0.0545294 -2.3063692
C -0.7726504 -0.4438403 -3.0451243
C 0.5011271 -1.0479293 -2.8620350
H 0.6969449 -1.6250884 -1.9638921
C 1.5050752 -0.9014690 -3.8100640
H 2.4714688 -1.3710367 -3.6452580
C 1.2837831 -0.1561718 -4.9747993
H 2.0731346 -0.0442062 -5.7121883
C 0.0326650 0.4440243 -5.1792388
H -0.1486944 1.0243197 -6.0800917
C -0.9744771 0.3058362 -4.2361502
H -1.9403559 0.7791087 -4.3971406

TS7• : FpPR• radical addtion to styrene
45
Energy = -2493.506420804
Fe 0.7768197 0.0776461 1.7996580
C 2.7071950 -0.3241896 2.5884409
H 2.9554160 -0.2747962 3.6390453
C 2.2253663 -1.4764646 1.8857182
H 2.0501641 -2.4534672 2.3143863
C 2.0148636 -1.1145437 0.5258460
H 1.6708042 -1.7813152 -0.2484809
C 2.3404904 0.2650308 0.3781679
H 2.2641422 0.8490865 -0.5279389
C 2.7755608 0.7464199 1.6551415
H 3.0839482 1.7594493 1.8743356
C -0.1793272 -0.6639505 3.0584513
O -0.7099281 -1.1483674 3.9720832
C 0.2498151 1.7094545 2.1581879
O -0.0192095 2.8075025 2.4209382
P -0.8593464 0.2105244 0.1506689
C -2.6396919 0.0606152 0.8266368
C -3.5573378 0.3541978 -0.3806582
H -3.2946131 1.3033859 -0.8598084
H -3.4961000 -0.4370461 -1.1325016
H -4.5991454 0.4162642 -0.0379807
H -2.6319920 2.1473131 1.4972094
C -2.8816945 1.1537883 1.8836033
H -2.2974896 0.9783797 2.7915599
H -3.9439970 1.1550813 2.1648732
C -3.0225479 -1.3046852 1.4187817
H -2.8827828 -2.1187257 0.7021047
H -2.4434367 -1.5399605 2.3124776
H -4.0846127 -1.2909391 1.7018218

C -1.0059431 -1.7099436 -1.4516686
H 0.0058166 -2.0073746 -1.2052975
H -1.7846572 -2.2307745 -0.9066565
C -1.2992887 -1.1754183 -2.6892745
H -2.3449294 -1.1020290 -2.9831851
C -0.3540045 -0.5791033 -3.6028219
C 1.0269125 -0.4739593 -3.3171656
H 1.4111253 -0.8645840 -2.3808522
C 1.9026618 0.1209331 -4.2178269
H 2.9597535 0.1904813 -3.9744981
C 1.4323379 0.6321290 -5.4331674
H 2.1195133 1.0962266 -6.1346555
C 0.0687499 0.5419884 -5.7336553
H -0.3080071 0.9384316 -6.6727363
C -0.8094695 -0.0504962 -4.8325434
H -1.8693234 -0.1132713 -5.0690528

TS8c⁻ : PC_2 ring-closing of anion $FpPRSty^-$
45
Energy = -2493.612561309
Fe 0.7573697 0.1606117 1.9047259
C 1.4925523 -0.8177250 3.6336976
H 1.7056923 -0.3209927 4.5703955
C 0.2290938 -1.3799764 3.2433271
H -0.6696098 -1.3869929 3.8449711
C 0.3653304 -1.9435207 1.9381534
H -0.4036563 -2.4573691 1.3832658
C 1.6942939 -1.6913502 1.5006301
H 2.1169019 -1.9717994 0.5457317
C 2.3938850 -1.0014916 2.5557434
H 3.4222199 -0.6681605 2.5177897
C -0.1987814 1.4515217 2.5500423
O -0.7708709 2.3441569 3.0659243
C 1.6098687 1.2230178 0.8284663
O 2.2245552 1.9336855 0.1273893
P -0.8785014 0.1298927 -0.3996187
C -2.6974420 0.0746833 0.2331956
C -3.6770811 -0.4554107 -0.8256824
H -3.6343095 0.1387764 -1.7429620
H -3.4786118 -1.5018842 -1.0812536
H -4.6999311 -0.4009562 -0.4223130
H -2.9965394 2.1446913 -0.3792038
C -3.0705574 1.5381715 0.5306257
H -2.4159550 1.9761563 1.2880152
H -4.1052324 1.5938568 0.8990903
C -2.8598184 -0.7708792 1.5033496
H -2.6054044 -1.8204726 1.3154444
H -2.2168794 -0.4010788 2.3050295
H -3.9066498 -0.7380403 1.8426737
C -0.6899729 -1.5743216 -1.1032236
H 0.3292754 -1.9655054 -1.0276849
H -1.3981955 -2.3067017 -0.7068509
C -1.0656626 -1.0121700 -2.4277824
H -2.0761349 -1.1674335 -2.7891661
C -0.1071634 -0.4896682 -3.3445450

C	1.2557163	-0.2628890	-2.9890245
H	1.5823660	-0.4657143	-1.9728099
C	2.1662390	0.2615798	-3.8997452
H	3.1933587	0.4320901	-3.5815093
C	1.7837931	0.5806496	-5.2095136
H	2.5015820	0.9869667	-5.9169919
C	0.4454025	0.3685417	-5.5822188
H	0.1205727	0.6103387	-6.5929522
C	-0.4730349	-0.1467801	-4.6779315
H	-1.5059830	-0.3000297	-4.9883618

TS8⁻ : anion FpPR⁻ addtion to styene
45
Energy = -2493.602710775

Fe	0.8138441	0.0592122	1.7955677
C	2.6652365	-0.4912402	2.6480832
H	2.8719872	-0.4777116	3.7095696
C	2.1563475	-1.6125491	1.9010703
H	1.9041573	-2.5820725	2.3082642
C	2.0183752	-1.2137678	0.5496709
H	1.6469873	-1.8303351	-0.2546277
C	2.4281934	0.1516386	0.4434798
H	2.4157102	0.7511403	-0.4561942
C	2.8383943	0.5921204	1.7464652
H	3.1991933	1.5799652	1.9982410
C	-0.1452118	-0.3942645	3.1655149
O	-0.6917200	-0.6991367	4.1572554
C	0.1562739	1.6915621	1.6772323
O	-0.0702073	2.8417365	1.7893977
P	-0.8720659	0.2258827	0.1207173
C	-2.6352599	0.0420393	0.8709377
C	-3.6073026	0.2456880	-0.3102416
H	-3.4136499	1.1966769	-0.8182526
H	-3.5165835	-0.5545240	-1.0497346
H	-4.6431476	0.2532714	0.0614805
H	-2.7624445	2.1350411	1.4757704
C	-2.9252065	1.1410997	1.9045472
H	-2.2911433	1.0448888	2.7906882
H	-3.9742607	1.0739911	2.2341897
C	-2.9138162	-1.3312080	1.5012848
H	-2.7374415	-2.1414473	0.7872557
H	-2.2787965	-1.5073447	2.3716438
H	-3.9640796	-1.3926064	1.8275002
C	-1.0310724	-1.5821837	-1.4682472
H	-0.0230560	-1.9282338	-1.2639863
H	-1.7906877	-2.1406834	-0.9291455
C	-1.3421118	-1.0905699	-2.7383423
H	-2.3907376	-1.0097141	-3.0228386
C	-0.3969472	-0.4941239	-3.6303452
C	0.9729945	-0.3074203	-3.2866661
H	1.3110907	-0.5977906	-2.2969713
C	1.8776633	0.2592768	-4.1770180
H	2.9150830	0.3866017	-3.8718649
C	1.4731644	0.6786593	-5.4519328
H	2.1838895	1.1213150	-6.1444164
C	0.1235778	0.5235496	-5.8078597
H	-0.2178689	0.8516146	-6.7880661
C	-0.7859683	-0.0408294	-4.9228410
H	-1.8287952	-0.1482899	-5.2185246

S4 References

References

(S1) Pangborn, A. B.; Giardello, M. A.; Grubbs, R. H.; Rosen, R. K.; Timmers, F. J. Safe and Convenient Procedure for Solvent Purification. *Organometallics* **1996**, *15*, 1518–1520.

(S2) Williams, D. B. G.; Lawton, M. Drying of Organic Solvents: Quantitative Evaluation of the Efficiency of Several Desiccants. *J. Org. Chem.* **2010**, *75*, 8351–8354.

(S3) Velian, A.; Cummins, C. C. Facile synthesis of Dibenzo-7λ^3-phosphanorbornadiene derivatives using magnesium anthracene. *J. Am. Chem. Soc.* **2012**, *134*, 13978–13981.

(S4) Plotkin, J. S.; Shore, S. G. Convenient preparation and isolation of pure potassium cyclopentadienyldicarbonylferrate, $K[(\eta^5\text{-}C_5H_5)Fe(CO)_2]$. *Inorg. Chem.* **1981**, *20*, 284–285.

(S5) Kolomeitsev, A. A.; Seifert, F. U.; Röschenthaler, G.-V. Simple preparation of difluorophosphoranes using anhydrous zinc and tetramethylammonium fluorides. *J. Fluor. Chem.* **1995**, *71*, 47–49.

(S6) Bailey, P. J.; Coxall, R. A.; Dick, C. M.; Fabre, S.; Henderson, L. C.; Herber, C.; Liddle, S. T.; Loroño-González, D.; Parkin, A.; Parsons, S. The first structural characterisation of a group 2 metal alkylperoxide complex: comments on the cleavage of dioxygen by magnesium alkyl complexes. *Chem. Eur. J.* **2003**, *9*, 4820–4828.

(S7) Iwasaki, T.; Shimizu, R.; Imanishi, R.; Kuniyasu, H.; Kambe, N. Cross-coupling Reaction of Alkyl Halides with Alkyl Grignard Reagents Catalyzed by Cp-Iron Complexes in the Presence of 1,3-Butadiene. *Chem. Lett.* **2018**, *47*, 763–766.

(S8) Jiang, X.; Chen, L.; Wang, X.; Long, L.; Xiao, Z.; Liu, X. Photoinduced Carbon Monoxide Release from Half-Sandwich Iron (II) Carbonyl Complexes by Visible Irradiation: Kinetic Analysis and Mechanistic Investigation. *Chem. Eur. J.* **2015**, *21*, 13065–13072.

(S9) Reger, D.-L.; Coleman, C. Preparation and reactions of the (dicarbonyl)(η^5-cyclopentadienyl)(tetrahydrofuran) iron cation: A convenient route to (dicarbonyl)(η^5-cyclopentadiemyl)(η^2-olefin) iron cations and related complexes. *J. Organomet. Chem.* **1977**, *131*, 153–162.

(S10) Geeson, M. B.; Transue, W. J.; Cummins, C. C. Organoiron-and Fluoride-Catalyzed Phosphinidene Transfer to Styrenic Olefins in a Stereoselective Synthesis of Unprotected Phosphiranes. *J. Am. Chem. Soc.* **2019**, *141*, 13336–13340.

(S11) Dub, P. A.; Batrice, R. J.; Gordon, J. C.; Scott, B. L.; Minko, Y.; Schmidt, J. G.; Williams, R. F. Engineering catalysts for selective ester hydrogenation. *Org. Process Res. Dev.* **2020**, *24*, 415–442.

(S12) Sheldrick, G. M. SHELXT–Integrated space-group and crystal-structure determination. *Acta Cryst. A* **2015**, *71*, 3–8.

(S13) Müller, P. Practical suggestions for better crystal structures. *Crystallogr. Rev.* **2009**, *15*, 57–83.

(S14) TURBOMOLE V7.4 2019, a development of University of Karlsruhe and Forschungszentrum Karlsruhe GmbH, 1989-2007, TURBOMOLE GmbH, since

2007; available from
http://www.turbomole.com.

(S15) Tao, J.; Perdew, J. P.; Staroverov, V. N.; Scuseria, G. E. Climbing the density functional ladder: Nonempirical meta–generalized gradient approximation designed for molecules and solids. *Phys. Rev. Lett.* **2003**, *91*, 146401.

(S16) Grimme, S.; Antony, J.; Ehrlich, S.; Krieg, H. A consistent and accurate ab initio parametrization of density functional dispersion correction (DFT-D) for the 94 elements H-Pu. *J. Chem. Phys.* **2010**, *132*, 154104.

(S17) Grimme, S.; Ehrlich, S.; Goerigk, L. Effect of the damping function in dispersion corrected density functional theory. *J. Comput. Chem.* **2011**, *32*, 1456–1465.

(S18) Weigend, F.; Ahlrichs, R. Balanced basis sets of split valence, triple zeta valence and quadruple zeta valence quality for H to Rn: Design and assessment of accuracy. *Phys. Chem. Chem. Phys.* **2005**, *7*, 3297–3305.

(S19) Klamt, A.; Schüürmann, G. COSMO: a new approach to dielectric screening in solvents with explicit expressions for the screening energy and its gradient. *J. Chem. Soc., Perkin Trans. 2* **1993**, 799–805.

(S20) Weigend, F. Accurate Coulomb-fitting basis sets for H to Rn. *Phys. Chem. Chem. Phys.* **2006**, *8*, 1057–1065.

(S21) Grimme, S. Supramolecular binding thermodynamics by dispersion-corrected density functional theory. *Chem. Eur. J.* **2012**, *18*, 9955–9964.

(S22) Eckert, F.; Klamt, A. Fast solvent screening via quantum chemistry: COSMO-RS approach. *AIChE J.* **2002**, *48*, 369–385.

(S23) Eckert, F.; Klamt, A. COSMOtherm, Version C3.0, Release 16.01, COSMOlogic GmbH & Co., Leverkusen, Germany, **2015**.

(S24) Zhao, Y.; Truhlar, D. G. Design of density functionals that are broadly accurate for thermochemistry, thermochemical kinetics, and nonbonded interactions. *J. Phys. Chem. A* **2005**, *109*, 5656–5667.

(S25) Qu, Z.-W.; Zhu, H.; Grimme, S. Acylation Reactions of Dibenzo-7-phosphanorbornadiene: DFT Mechanistic Insights. *ChemistryOpen* **2019**, *8*, 807–810.

(S26) Zhu, H.; Qu, Z.-W.; Grimme, S. Borane-Catalyzed Hydrogenation of Tertiary Amides Activated by Oxalyl Chloride: DFT Mechanistic Insights. *Eur. J. Org. Chem.* **2019**, *2019*, 4609–4612.

(S27) Zhu, H.; Qu, Z.-W.; Grimme, S. Reduction of phosphine oxide by using chlorination reagents and dihydrogen: DFT Mechanistic insights. *Chem. Eur. J.* **2019**, *25*, 4670–4672.

(S28) Qu, Z.-W.; Zhu, H.; Katsyuba, S. A.; Mamedova, V. L.; Mamedov, V. A.; Grimme, S. Acid-Catalyzed Rearrangements of 3-Aryloxirane-2-Carboxamides: Novel DFT Mechanistic Insights. *ChemistryOpen* **2020**, *9*, 743–747.

(S29) Qu, Z.-W.; Zhu, H.; Grimme, S. Frustrated Lewis pair catalyzed reduction of carbon dioxide using hydroboranes: new DFT mechanistic insights. *ChemCatChem* **2020**, *12*, 3656–3660.

(S30) Qu, Z.-W.; Zhu, H.; Grimme, S. Mechanistic Insights for Iodane Mediated Aromatic Halogenation Reactions. *ChemCatChem* **2020**, *12*, 6186–6190.

(S31) Qu, Z.-W.; Zhu, H.; Grimme, S. Mechanistic Insights for Aniline-Catalyzed Halogenation Reactions. *ChemCatChem* **2020**, *12*, 5369–5373.

(S32) Qu, Z.-W.; Zhu, H.; Grimme, S. LiAlH4-catalyzed Imine Hydrogenation with Dihydrogen: New DFT Mechanistic Insights. *ChemCatChem* **2021**, *13*, 3401–3404.

(S33) Qu, Z.-W.; Zhu, H.; Grimme, S. Mechanistic Insights for Dimethyl Sulfoxide Catalyzed Aromatic Chlorination Reactions. *ChemCatChem* **2021**, *13*, 207–211.

(S34) Qu, Z.-W.; Zhu, H.; Grimme, S. Mechanistic insights for nitromethane activation into reactive nitrogenating reagents. *ChemCatChem* **2021**, *13*, 2132–2137.

(S35) Qu, Z.-W.; Zhu, H.; Zhukova, N. A.; Katsyuba, S. A.; Mamedov, V. A.; Grimme, S. Mechanistic Insights for Acid-catalyzed Rearrangement of Quinoxalin-2-one with Diamine and Enamine. *ChemCatChem* **2021**, *13*, 1503–1508.

(S36) Goerigk, L.; Hansen, A.; Bauer, C.; Ehrlich, S.; Najibi, A.; Grimme, S. A look at the density functional theory zoo with the advanced GMTKN55 database for general main group thermochemistry, kinetics and noncovalent interactions. *Phys. Chem. Chem. Phys.* **2017**, *19*, 32184–32215.

(S37) Schreckenbach, G.; Ziegler, T. Calculation of NMR shielding tensors using gauge-including atomic orbitals and modern density functional theory. *J. Phys. Chem.* **1995**, *99*, 606–611.
